# Unmodificated stepless regulation of CRISPR/Cas12a multi-performance

**DOI:** 10.1093/nar/gkad748

**Published:** 2023-09-27

**Authors:** Rong Zhao, Wang Luo, You Wu, Li Zhang, Xin Liu, Junjie Li, Yujun Yang, Li Wang, Luojia Wang, Xiaole Han, Zhongzhong Wang, Jianhong Zhang, Ke Lv, Tingmei Chen, Guoming Xie

**Affiliations:** Key Laboratory of Clinical Laboratory Diagnostics (Chinese Ministry of Education), College of Laboratory Medicine, Chongqing Medical Laboratory Microfluidics and SPRi Engineering Research Center, Chongqing Medical University, Chongqing 400016, PR China; Key Laboratory of Clinical Laboratory Diagnostics (Chinese Ministry of Education), College of Laboratory Medicine, Chongqing Medical Laboratory Microfluidics and SPRi Engineering Research Center, Chongqing Medical University, Chongqing 400016, PR China; Key Laboratory of Clinical Laboratory Diagnostics (Chinese Ministry of Education), College of Laboratory Medicine, Chongqing Medical Laboratory Microfluidics and SPRi Engineering Research Center, Chongqing Medical University, Chongqing 400016, PR China; Key Laboratory of Clinical Laboratory Diagnostics (Chinese Ministry of Education), College of Laboratory Medicine, Chongqing Medical Laboratory Microfluidics and SPRi Engineering Research Center, Chongqing Medical University, Chongqing 400016, PR China; Key Laboratory of Clinical Laboratory Diagnostics (Chinese Ministry of Education), College of Laboratory Medicine, Chongqing Medical Laboratory Microfluidics and SPRi Engineering Research Center, Chongqing Medical University, Chongqing 400016, PR China; Key Laboratory of Clinical Laboratory Diagnostics (Chinese Ministry of Education), College of Laboratory Medicine, Chongqing Medical Laboratory Microfluidics and SPRi Engineering Research Center, Chongqing Medical University, Chongqing 400016, PR China; Key Laboratory of Clinical Laboratory Diagnostics (Chinese Ministry of Education), College of Laboratory Medicine, Chongqing Medical Laboratory Microfluidics and SPRi Engineering Research Center, Chongqing Medical University, Chongqing 400016, PR China; The Center for Clinical Molecular Medical Detection, The First Affiliated Hospital of Chongqing Medical University, Chongqing 400016, PR China; Key Laboratory of Clinical Laboratory Diagnostics (Chinese Ministry of Education), College of Laboratory Medicine, Chongqing Medical Laboratory Microfluidics and SPRi Engineering Research Center, Chongqing Medical University, Chongqing 400016, PR China; Key Laboratory of Clinical Laboratory Diagnostics (Chinese Ministry of Education), College of Laboratory Medicine, Chongqing Medical Laboratory Microfluidics and SPRi Engineering Research Center, Chongqing Medical University, Chongqing 400016, PR China; Key Laboratory of Clinical Laboratory Diagnostics (Chinese Ministry of Education), College of Laboratory Medicine, Chongqing Medical Laboratory Microfluidics and SPRi Engineering Research Center, Chongqing Medical University, Chongqing 400016, PR China; Key Laboratory of Clinical Laboratory Diagnostics (Chinese Ministry of Education), College of Laboratory Medicine, Chongqing Medical Laboratory Microfluidics and SPRi Engineering Research Center, Chongqing Medical University, Chongqing 400016, PR China; Department of Neurosurgery, The First Affiliated Hospital of Chongqing Medical University, Chongqing 400016, PR China; Key Laboratory of Clinical Laboratory Diagnostics (Chinese Ministry of Education), College of Laboratory Medicine, Chongqing Medical Laboratory Microfluidics and SPRi Engineering Research Center, Chongqing Medical University, Chongqing 400016, PR China; Key Laboratory of Clinical Laboratory Diagnostics (Chinese Ministry of Education), College of Laboratory Medicine, Chongqing Medical Laboratory Microfluidics and SPRi Engineering Research Center, Chongqing Medical University, Chongqing 400016, PR China

## Abstract

As CRISPR technology is promoted to more fine-divided molecular biology applications, its inherent performance finds it increasingly difficult to cope with diverse needs in these different fields, and how to more accurately control the performance has become a key issue to develop CRISPR technology to a new stage. Herein, we propose a CRISPR/Cas12a regulation strategy based on the powerful programmability of nucleic acid nanotechnology. Unlike previous difficult and rigid regulation of core components Cas nuclease and crRNA, only a simple switch of different external RNA accessories is required to change the reaction kinetics or thermodynamics, thereby finely and almost steplessly regulating multi-performance of CRISPR/Cas12a including activity, speed, specificity, compatibility, programmability and sensitivity. In particular, the significantly improved specificity is expected to mark advance the accuracy of molecular detection and the safety of gene editing. In addition, this strategy was applied to regulate the delayed activation of Cas12a, overcoming the compatibility problem of the one-pot assay without any physical separation or external stimulation, and demonstrating great potential for fine-grained control of CRISPR. This simple but powerful CRISPR regulation strategy without any component modification has pioneering flexibility and versatility, and will unlock the potential for deeper applications of CRISPR technology in many finely divided fields.

## Graphical Abstract

**Graphical Abstract:**
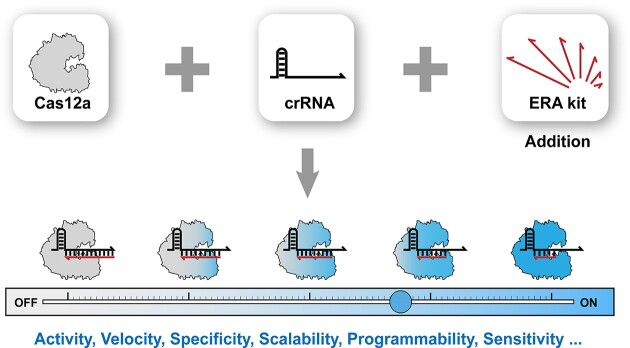

## INTRODUCTION

The revolutionary technology of clustered regularly interspaced short palindromic repeats (CRISPR) has transformed the fields of molecular biology including gene editing, intracellular imaging, transcriptional regulation, gene therapy, molecular diagnostics, molecular biochemical circuits and more. However, as the range of applications continues to expand, the inherent performance of CRISPR/Cas alone is no longer sufficient to meet diverse needs of an increasing number of finely divided applications. In a new development stage, how to regulate the performance of CRISPR/Cas to better meet different needs is gradually becoming the focus of research. CRISPR RNA (crRNA), which is responsible for recognition, and Cas nuclease, which is responsible for cleavage, are the core components of CRISPR/Cas. Much work has been done to modify these two components to enhance or attenuate the performance of CRISPR/Cas. To reduce the off-target rate of gene editing or to improve the accuracy of nucleic acid recognition, more specific Cas subspecies have been identified from different strains (1,2) or developed through protein engineering (3–5), while other work has targeted crRNA for truncation (6), embedding in DNA (7,8), insertion of secondary structures (9,10) and modification of chemical bonds (11) to improve specificity. To improve the efficiency of gene editing and the sensitivity of nucleic acid recognition, Casπ (Cas12l) (12) and Cas13 variants (13) with high trans-cleavage activity were modified, and other efforts were made to increase the sensitivity by crRNA extension (14), tandem (15,16) and cascade (enzymatic release of crRNA) (17) or R-loop restoration (18). To improve the controllability and safety of molecular reaction networks, gene editing or cell imaging, light-guided protein-tagged Cas12a (19) has been developed, and other work has been done to set up blockers (20–22), chemical modifications (23,24), secondary structures (G-quadruplex, etc.) (25,26) and gating devices for crRNA. To improve the compatibility between the nucleic acid amplification system and the CRISPR system, suboptimal PAM sequences of Cas12a were screened (27), a PC-linker modified protective oligo was used to programmatically activate Cas12a (28) and glycerol additives (29) were used to separate the recombinase polymerase amplification (RPA) from the CRISPR/Cas12a system.

**Scheme 1.**
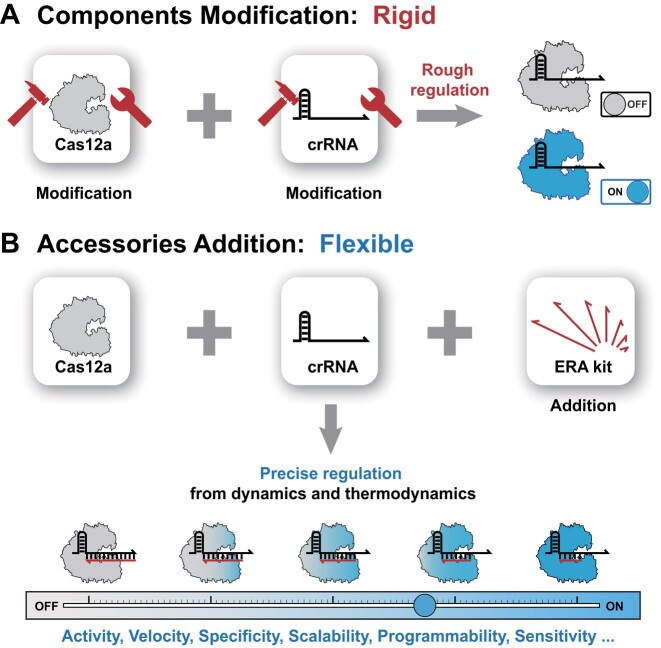
**(A)** Modification of the recognition component crRNA or the execution component Cas nuclease is difficult and rigid, and allows achievement of only rough regulation of some CRISPR/Cas performances. **(B)** Adding the RNA complementary strands as accessories to the original components is simple and flexible, and allows precise control of many CRISPR/Cas performances by selecting different ERAs to alter the dynamics and thermodynamics of activator binding. ‘ON/OFF’ indicates that Cas12a is in ‘activatable/inactivatable’ state.

These efforts effectively broadened the scope of CRISPR/Cas applications, but as research deepened and demand grew, emerging shortcomings hampered its next stage of development. First, the development of new Cas nucleases or modifications to them struggled to keep pace with the rapid growth of finely divided application areas. Although it is relatively easy to modify crRNAs by various methods, when faced with different sequence environments, constant trial and error is required to find the best crRNA design. In addition, chemical modification methods are increasingly diverse (30–32), and some of these may pose biohazards. More importantly, the ‘one-touch’ activation and ultra-high cutting efficiency of CRISPR/Cas also makes the tunable range very narrow, and most means are only capable of coarse performance ON/OFF control or limited stepped regulation (Scheme 1A). It follows that the development of a simple and flexible generalized strategy to finely control the multiple properties of CRISPR may be able to better meet the increasingly diverse needs for CRISPR/Cas performance in different fields.

Toehold-mediated strand displacement reaction (TMSD) serves as an important basis for dynamic nucleic acid nanotechnology. In this reaction, the invading strand binds to a short complementary single-stranded domain (toehold) on a double-stranded substrate, triggering subsequent branch migration and eventual replacement of the substrate's protector to be the thermodynamically most stable state (33). TMSD follows precise Watson–Crick base pairing and has not only strict specificity but also a very wide range of tunability (34). A variety of tuning methods have been developed to alter the rate, yield and specificity of TMSD (35–40), and these methods are universal and very tolerant of systems with different sequence compositions. In previous studies, we found that treatment of activators into toehold-containing duplexes was more specific (41), suggesting that further binding of TMSD to CRISPR/Cas may be possible. Here, we designed an external RNA accessory (ERA) toolkit for Cas12a, which does not activate Cas12a but binds to part of the spacer region of crRNA to form a different toehold-containing double-stranded structure, allowing the simple pairwise binding of the activator to crRNA to be converted into a conditional response determined by TMSD (ERA acts as ‘protector’). The attachment of ERAs with different structures, lengths and mismatches can alter the kinetics and thermodynamics of the ERA–crRNA complex reaction with the activator, allowing precise control of the degree of activation of the Cas nuclease, which further allows control of a number of properties, including activity, speed, specificity, programmability, scalability, sensitivity etc. (Scheme 1B). In this work, we meticulously investigated the fine-tuning methods for each performance of CRISPR/Cas, and explored the compatibility and heterogeneity of DNA dynamic nanotechnology with the CRISPR/Cas system. Interestingly, we also achieved spatially continuous but temporally isolated CRISPR activation control by ERA, thus overcoming the mutual exclusion problem of CRISPR/Cas signal output module and amplification module in an isothermal one-pot assay, improving the sensitivity and signal-to-noise ratio of the isothermal one-pot assay and demonstrating the great potential of fine control of CRISPR performance.

Compared to the modifications of Cas nuclease or crRNA components, ERA has unprecedented flexibility as an add-on that can be added at will to reasonably meet different needs, allowing crRNA and Cas-identical CRISPR systems to exhibit completely different properties. In addition, ERA regulation is solely based on kinetics and thermodynamics and is extremely inclusive, with the potential to be interoperable with other developed component modification strategies, making it a powerful complement to existing regulatory approaches. And this work is not limited to Cas12a, even in other RNA-targeting Cas nucleases, such as Cas13, can also achieve similar results by adding the corresponding external DNA accessory toolkits (EDA). More importantly, ERA can serve as a bridge to combine CRISPR technology with dynamic DNA nanotechnology, linking CRISPR systems that can only achieve simple ON/OFF control with a broader and deeper network of molecular responses to achieve more and more comprehensive functions.

## METHODS AND MATERIALS

### Materials

Oligonucleotides were purchased from Beijing Genomics Institution (Beijing, China), Tsingke Biotechnology Co. Ltd (Beijing,China), and Sangon Biotech. Co. Ltd (Shanghai, China). Lba Cas12a (Cpf1, 20 μM) and rCutSmart buffer (50 mM potassium acetate, 20 mM Tris-acetate, 10 mM magnesium acetate, 100 μg/ml bovine serum albumin (BSA), pH 7.9)) were ordered from MAGIGEN (Guangzhou, China) and New England Biolabs (Beijing, China). Large Fragment Bst DNA polymerase, 10× Bst Reaction Bufffer (200 mM Tris-HCl, 100 mM KCl, 100 mM (NH_4_)_2_SO_4_, 20 mM MgSO_4_, 1% Triton X-100) and 100 mM MgSO_4_ were bought from New England Biolabs (Beijing, China). dATP (100 mM), dTTP (100 mM) and dCTP (100 mM) were bought from Sangon Biotech. Co. Ltd (Shanghai, China). Dithiothreitol (DTT) was ordered from Sangon Biotech. Co. Ltd (Shanghai, China). *N*,*N*,*N′*,*N*′-Tetramethylethylenediamine (TEMED) and 30% acrylamide/bis solution were provided by Sigma-Aldrich (St. Louis, MO, USA). DNA loading buffer (6×) and Gel Red nucleic acid dye were ordered from TaKaRa Biotech (Dalian, China). All chemical reagents were of analytical grade, and RNase-free water was used throughout this study.

### Instruments

Time-based fluorescence data were acquired using a Rotor-Gene 6000 instrument (Corbett Research, Mortlake, Australia). The temperature was set to 25°C, and gain was set to default. All fluorescent signals were monitored under the yellow channel (530 nm/555 nm). For one-pot method assays with PER, the temperature was set to 37°C. Assembly of oligonucleotides was achieved by annealing in a PCR instrument (CFX96, Bio-Rad, USA), the procedure was 95°C for 5 min and then decreased from 95 to 12°C at 0.1°C/s. Gel images were obtained on an electrophoresis apparatus (DYY-6C, LIUYI, China) and imaging system (Bio-Rad Laboratories, USA).

## METHODS

### Design and calculation

The sequence designs (Supplementary Table S1, Schemes S3–S21) were supported by NUPACK and SnapGene. The thermodynamic parameters were calculated by NUPACK. Discrimination factor formula: DF = ([*F*_PM_] – background)/([*F*_MM_] – background).

### Preparation of crRNA-Cas12a complexes

Cas12a and crRNA were mixed at a 1:1 ratio (2 μM:2 μM) and preincubated at 25°C for 1 h to promote the ribonucleoprotein complex (RNP) formation.

### Preparation of ERA–crRNA–Cas12a complexes.

crRNA (2 μM) was annealed with various ERA (ERA:crRNA = 2:1) to from ERA–crRNA complexes. Subsequently, Cas12a (2 μM) was added and incubated at 25°C for 1 h.

### CRISPR/Cas reaction system

The crRNA–Cas12a or ERA–crRNA–Cas12a complexes were diluted to 20 nM in a solution containing 1 × Cutsmart Buffer, 250 nM Cas-reporter and 1 mM DTT, and the final volume was 20 μl. The added activators (single-stranded, double-stranded, perfectly matched or mismatched) concentration were 40 nM.

### PER procedure

The reaction was carried out in a solution (20 μl) containing varied concentration of target (MiRNA-21), 1 × ThermoPol buffer, 10 mM MgSO_4_ buffer, 10 nM protector/hairpin-template double-stranded probes, 200 nM primers, 100 μM dNTPs and 0.2 U Bst Large Fragment DNA polymerase at 37°C for different times, followed by heating at 80°C for 20 min to terminate the reaction.

### Two-step assay

After the PER procedure, 80 nM crRNA–Cas12a or ERA–crRNA–Cas12a complexes, 500 nM Cas-reporter, 1 × rCutSmart buffer, 1 mM DTT was added into the reaction system, the final volume was 20 μl and the fluorescence results were measured at 37°C.

### One-pot assay

The PER system and the previous CRISPR/Cas system were mixed together and measured at 37°C.

### Fluorescence versus cleaved reporter concentration calibration

Background-subtracted fluorescence signals were obtained by subtracting the signal of a buffer-only sample from the signal obtained from titrated quantities of fully cleaved Cas-reporters. Reporters were pre-cleaved by subjecting them to the trans-cleavage reaction of the activated crRNA–Cas12a complex. For this, 20 nM activated Cas enzyme (activated using ssDNA target) was mixed with varying reporter concentrations of 31.25 nM, 62.5 nM, 125 nM, 250 nM, 500 nM, 1 μM and 2 μM with a final volume of 20 μl. The trans-cleavage reaction was performed at 37°C for ∼10 h (42). At the end of this pre-cleavage step, we verified that the fluorescence signal of each reaction was constant in time. We performed a linear fit of the background-subtracted, steady-state (fully cleaved) fluorescence signal (*F*_cl_) to the cleaved reporter concentration (*c*_cl_) using ORIGIN software. The relationship between the background-subtracted fluorescence (*F*_ucl_) and uncleaved reporter concentration (*c*_ucl_) were also analyzed. As mentioned previously, background subtraction here refers to subtracting the signal obtained from a buffer-only sample from the signal obtained from titrated quantities of uncleaved reporters. We also performed a linear fit of the background-subtracted, uncleaved fluorescence signal *F*_ucl_ to the uncleaved reporter concentration *c*_ucl_ using ORIGIN software.

### Michaelis-Menten kinetics calculation

We hypothesize that the background-subtracted fluorescence as a function of time *F(t)* measured during trans-cleavage experiment is the sum of fluorescence from cleaved reporters *F*_cl_*(t)* and uncleaved reporters *F*_ucl_*(t)*, as the quenching of a fluorophore in an intact reporter is imperfect. Thus, we write


(1) \begin{eqnarray*}F\left( t \right) = {F}_{\mathrm{cl}}\left( t \right)\ + {F}_{\mathrm{ucl}}\left( t \right)\end{eqnarray*}

Displacing the calibration curve equations from Supplementary Figure S11 in Equation (1), we obtain


(2) \begin{eqnarray*}F\left( t \right) = 0.14466\ {c}_{\mathrm{cl}}\left( t \right)\ + 0.00007\ {c}_{\mathrm{ucl}}\left( t \right)\end{eqnarray*}

From mass conservation, ${c}_{\mathrm{cl}}( t ) + \ {c}_{\mathrm{ucl}}( t ) = \ {c}_0$ where ${c}_0$ is the initial concentration of uncleaved reporters. Thus, Equation (2) can be rewritten as


(3) \begin{eqnarray*}F\left( t \right) &=& 0.14466{\mathrm{\ }}{c}_{\mathrm{cl}}\left( t \right){\mathrm{\ }} + 0.00007\ {c}_{\mathrm{ucl}}\left( t \right){\mathrm{\ }} \nonumber\\ &=& 0.14459{\mathrm{\ }}{c}_{\mathrm{cl}}\left( t \right){\mathrm{\ }} + 0.00007\ {c}_0\end{eqnarray*}

The reaction velocities $d{c}_{cl}/dt$ in nM/s is obtained by differentiating Equation (3) with respect to time as


(4) \begin{eqnarray*}\frac{{d{\mathrm{\ }}{c}_{\mathrm{cl}}}}{{dt}} = \frac{1}{{0.14459}} \times \frac{{dF}}{{dt}}{\mathrm{\ }}\end{eqnarray*}

Last, the cleaved reporter concentration ${c}_{\mathrm{cl}}$ (in nM) versus time is estimated using Equation (3) as


(5) \begin{eqnarray*}{c}_{\mathrm{cl}}\left( t \right)\left( {{\mathrm{in\ nM}}} \right) = \frac{{F\left( t \right) - 0.00007{c}_0}}{{0.14446}} = \frac{{F\left( t \right) - 0.00875}}{{0.14446}}{\mathrm{\ }}\end{eqnarray*}

### Michaelis–Menten Kinetics Measurements and Data Analysis

The regular Michaelis–Menten equation can eventually be written in the following form


(6) \begin{eqnarray*}\nu = \frac{{d\left[ P \right]}}{{dt}} = {k}_{\mathrm{cat}}{E}_0\frac{{\left[ S \right]}}{{{K}_{\mathrm{M}} + \left[ S \right]}}\ \end{eqnarray*}

Furthermore, in most applications of CRISPR-diagnostics, the concentration of the substrate is significantly smaller than (or, at most on the same order of) the Michaelis–Menten constant of the enzyme, $[ {\mathrm{S}} ] \ll {K}_{\mathrm{M}}$. Typically, ${{\mathrm{S}}}_0\sim{\mathrm{O}}({100{\mathrm{\ nM}}})$, while *K*_M_ ∼ O(100 to 1 μM). Thus, we can use the $[{\mathrm{S}}] \ll {K}_{\mathrm{M}}$ approximation to further derive an expression for the evolution of the product (reporter cleavage) versus time, over (long) time scales on the order of the time to complete the reaction. Specifically, we can rewrite Equation (6) as


(7) \begin{eqnarray*}\frac{{d\left[ P \right]}}{{dt}} \approx \frac{{{k}_{\mathrm{cat}}{E}_0}}{{{K}_{\mathrm{M}}}}\left( {{S}_0 - \left[ P \right]} \right)\end{eqnarray*}

Trans-cleavage reactions were initiated by the addition of Cas-reporter at concentration of 125 nM. We maintained the concentration of target-activated crRNA constant at 1 nM throughout the 20-μl reaction volume, except when the toehold was too short resulting in the too-slow recovery of Cas12a *trans*-cleavage activity, so that changes in 1 nM Cas12a activity were difficult to characterize by changes in fluorescence signal and we increased the concentration of activated Cas12a to 5 nM.

Reactions were carried out at 25°C and fluorescence readouts were obtained every 20 s. The first 600 s of data at each reporter concentration was fitted using linear regression to obtain initial reaction velocities in units of AU/s. A calibration curve was used to convert reaction velocities from AU/s to nM/s. The measured reaction velocities versus reporter (substrate) concentration data were fitted to the Michaelis–Menten equation (Equation (6)) using ORIGIN software to obtain *k*_cat_ and *K*_M_.

### Statistics and reproducibility

All experiments were repeated at least three times and, unless specified otherwise, statistical analysis was performed using ORIGIN software and quantitative data are typically derived from averaging three individual experiments. The unpaired two-tailed *t*-test was performed to compare fluorescence signal of two cohorts. A *P* value <0.05 was considered to be statistically significant. When quantitative data are shown, they are typically derived from averaging three individual experiments.

## RESULTS AND DISCUSSION

### Multidimensional control of activation velocity and cleavage activity

Binding of the invasion strand to the substrate toehold is the initiation step of TMSD and the key rate-limiting factor for the reaction kinetics (43). In DNA displacement reactions (25°C), where for the average toehold sequence, the rates saturated for a 6 nt toehold is 10^5^ times greater than that for a 1 nt toehold (44). A similar range of relative rates was observed in RNA displacement reactions (45). There are relatively few studies on the replacement of double-stranded RNA substrates by DNA invasion strands (46). To explore whether the TMSD of crRNA, ERA and DNA activator in CRISPR system have similar toehold rate-limiting patterns, we designed different lengths of ERA to bind to the spacer region of crRNA to form double-stranded RNA substrates with different toehold lengths. Considering the DNA-based TMSD reactions almost do not have directionality, (47) the RNA-based reactions do have (45), coupled with the directional crRNA and Cas12a assembly (48,49), here we further designed two sets of ERAs with toeholds near the PI domains (5′ toehold) or Nuc domains (3′ toehold) of the Cas12a protein for comparison to explore the relevant directionality patterns.

**Figure 1.**
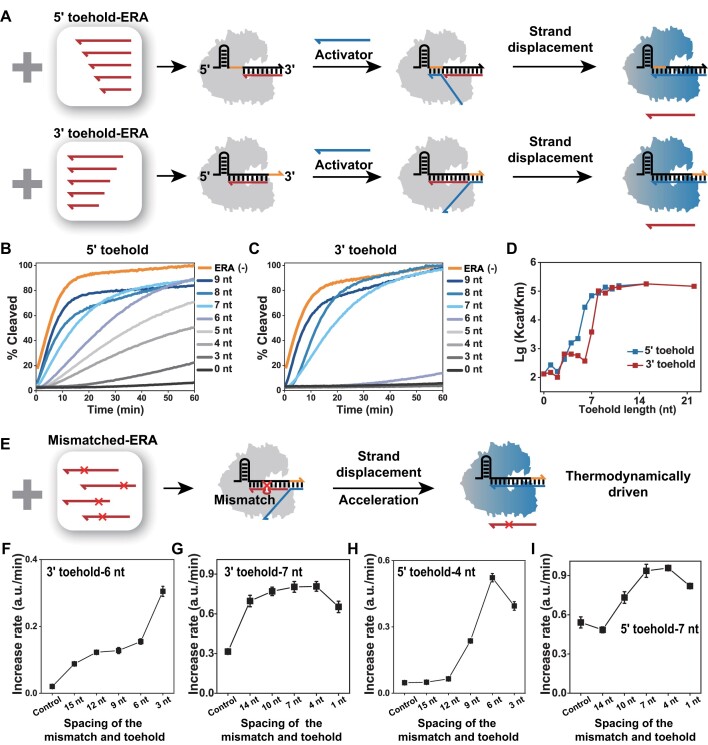
**(A)** ERAs toolkit bind to crRNAs to form double-stranded RNA substrates with toeholds of different directions and lengths, and activators activate Cas12a via TMSD. Activation efficiency of ERA–crRNA–Cas12a with different lengths of 5′ toehold **(B)** or 3′ toehold **(C)**. **(D)** Relationship between toehold length and direction on the ERA–crRNA–Cas12a catalytic efficiency (*k*_cat_/*K*_M_). **(E)** Mismatched ERAs toolkit bind to crRNA to form substable substrates, and eliminating the unstable state can accelerate ERA-controlled Cas12a activation. Activation efficiency of ERA–crRNA–Cas12a (different ERA mismatch sites) with 3′ toehold-6 nt **(F)** or −7 nt **(G)**, 5′ toehold-4 nt **(H)** or −7 nt **(I)**. ERA (−) means no ERA is added. Orange represents the toehold in the crRNA. Error bars represented the standard deviation calculated from three independent experiments.

As shown in Figure 1A, the input activators first bind to the 5′ or 3′ toehold, triggering a base-by-base branch migration that displaces the ERA and eventually activate Cas12a by binding completely to the crRNA (the Gibbs free energy changes (ΔG) of different ERA–crRNA complexes are summarized in Supplementary Table S2). The result shows that the length of 5′ toehold has a very regular effect on the *trans*-cleavage rate, which could approach saturation (>80%) at toehold >6 nt, and the cleavage rate corresponding to individual base changes forms a uniform gradient change (Figure 1B). Supplementary Figure S1 shows the fluorescence cleavage rate at 20 min, and Supplementary Figure S2 shows the results of activator at low concentrations (1 nM and 100 pM). However, Cas12a is barely activated when 3′ toehold <7 nt, and only when toehold >7 nt does it reflect a gradient change in cleavage rate (Figure 1C, Supplementary Figure S3). In Figure 1D, we further calculate and compare the relationship between the catalytic efficiency of Cas12a (enzyme turnover/Michaelis–Menten constant, *k*_cat_/*K*_M_) and the length and orientation of toehold from the perspective of enzyme activity. The pattern of regulation of Cas12a activity by ERA at 5′ toehold is consistent with that of TMSD, but probably due to the higher stability of the RNA–RNA base pair, DNA displacement of RNA substrate requires toehold up to 9 nt to have a near-saturated reaction rate. In the case of 3′ toehold, the rate again reaches saturation at length greater than 9 nt, but up to that point is considerably slower than the 5′ toehold, as detailed in Supplementary Table S3 for the enzyme kinetic parameters. This 5′ directional advantage is very similar to that of RNA-based TMSD, and they may share the same principle: The invading strand at the 5′ end gains additional stabilization from cross-stacking interaction with the substrate strand, hence the probability for the invading strand to fall off from the 5′ toehold is lower and the displacement rate is increased. In Supplementary Figures S4 and S5, we find that the addition ratio of ERA can also be used to fine-tune the activity of Cas12a. Similar conclusions can be obtained in a series of experiments using a different set of sequences (Supplementary Figures S6–S10, Supplementary Tables S4–S5). It follows that it is entirely possible for the behavior of ERA-controlled Cas12a to be predicted by highly general, regulated and mature TMSD.

Double-stranded activation of CRISPR/Cas has a more prominent position in biological applications such as gene editing, unlike the simple single-stranded activation mode, which requires additional steps of PAM recognition, seed domain binding and R-loop formation (50,51). Nevertheless, it is interesting to note that ERA still has a strong regulation of CRISPR/Cas12a in the double-stranded activation mode. As shown in Supplementary Figures S12–S15, in different sets of crRNA sequences, the efficiency of double-strand activation can be gradient-regulated by the length and direction of toehold, which is due to the TMSD of the deconvoluted target strand (TS) and ERA–crRNA. Although the 3′ toehold is far from the PAM sequence, opposite to the starting point of unstranding of the double-stranded activator and theoretically TS is difficult to react with crRNA, surprisingly, the double-stranded activation of the 3′ toehold is very regular, even faster than the 5′ toehold (Supplementary Figures S12, S14). This may be attributed to the conformational change of Cas12a, which weakens the stability of the double-stranded activator and mediates the proximal binding of PAM, while TMSD mediates the distal binding of PAM.

When a base mismatch is introduced in the double-stranded substrate of TMSD, the structure is in a substable state with a higher thermodynamic potential, providing an additional thermodynamic drive to promote mismatched protector replacement by the invading strand (52). Thermodynamically, the elimination of mismatches by TMSD leads to a decrease in enthalpy and a negative Δ*G*, which increases the reaction yield (Supplementary Tables S6–S8). In addition, the kinetics and thermodynamics are coupled near toehold, and thermodynamic driving also accelerates the kinetics of branch migration (elimination of mismatches near toehold increases the reaction rate by about two orders of magnitude) (52). Accordingly, as shown in Figure 1E, we designed different positionally mismatched ERAs for introducing mismatches to ERA–crRNA complexes to accelerate branch migration after toehold binding.

Experiments show that the activator can barely activate Cas12a when the length of the 3′ toehold is 6 nt (less than 7 nt), but when there is a mismatch on the ERA, the activation is significantly accelerated (Figure 1F); even the already fast 3′ toehold-7 nt can be further accelerated (Figure 1G). This is also true in the opposite direction, where both the otherwise slower 5′ toehold-4 nt and the faster 5′ toehold-7 nt are accelerated by thermodynamic drive (Figure 1H and I). More importantly, by setting mismatch sites at different distances from toehold on ERA, we found that early mismatch elimination (ERA closer to toehold) had a more pronounced accelerating effect on the TMSD rate, with the highest reaction rate when the mismatch was eliminated early rather than immediately. This pattern is also independent of toehold orientation and is highly consistent with conventional TMSD. The corresponding fluorescence kinetic curves are shown in Supplementary Figures S16–S19. Flexible control of Cas12a activity was also achieved when dsDNA was used as the activator (Supplementary Figures S20–S23).

Toehold length and orientation, ERA mismatch position and addition ratio can act as controllers of different dimensions, complementing each other to achieve extremely fine, nearly stepless regulation of Cas12a activity. Moreover, this strategy is grounded in the kinetic and thermodynamic mechanisms of TMSD without any crRNA modification at all, which is highly predictable and versatile, greatly simplifying many unnecessary steps of crRNA sequence design and optimization, and is expected to meet diverse needs of widely used scenarios.

### Kinetics-driven control of activation specificity

**Figure 2.**
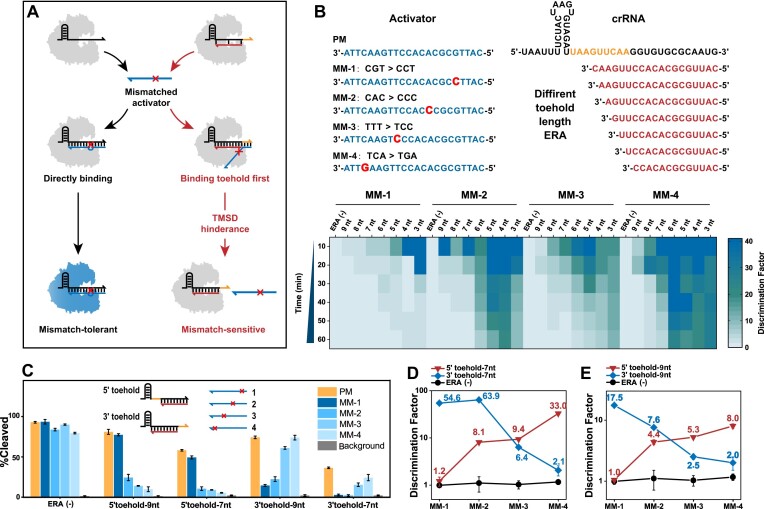
**(A)** Discrimination of single-base-mismatched activators by uncontrolled or ERA-controlled Cas12a. **(B)** DF of ERA-controlled Cas12a with different 5'-toehold lengths in the recognition of different mismatched activators. **(C)** Activation efficiency of ERA-controlled Cas12a by different mismatched activators when toehold direction is 5' or 3' and length is 7 nt or 9 nt. **(D, E)** Relationship between discriminantion factors and mismatch site, toehold length and direction. Orange represents the toehold in the crRNA. ERA (-) means no ERA is added. Error bars represented the standard deviation calculated from three independent experiments.

CRISPR/Cas is typically very tolerant of activator recognition, and similar sequences of activators can easily misactivate Cas nucleases (53,54). This is the main reason why CRISPR/Cas is off-target in gene editing and generates false positive signals in molecular assays. Tedious crRNA modification and optimization is required to improve even a little specificity (many crRNA design and simulation data packages have been developed to reduce the cost of trial and error) (55–57). Fortunately, single-base discrimination is another feature of TMSD (58,59), that promises to greatly improve the single-base specificity of CRISPR/Cas with the introduced ERA toolbox without modifying crRNA at all. As shown in Figure 2A, the direct binding of crRNA to the activator is so rapid that mismatches can cause only a very small loss of kinetics and is virtually indistinguishable from correct activation. By contrast, for ERA-controlled Cas12a, where TMSD is a prerequisite for activation, mismatches can inhibit incorrect activation by significantly suppressing the kinetics of TMSD, reflecting a distinct specificity.

To explore the ability of ERA to modulate specificity, we designed four different positionally mismatched activators (MM-1, MM-2, MM-3, MM-4). As shown in Figure 2B, among 5′ toeholds, MM-1 is the farthest from the toehold and had the lowest discrimination factor (DF) (median DF = 1.9 at 20 min); MM-4 is the closest to the toehold and had the highest DF (median DF = 31.6 at 20 min); and specificity was negatively correlated with toehold length. Considering that the activation efficiency is positively correlated with toehold length, MM-4 with mismatch sites the closest to the 5′ toehold may have the best discrimination effect. The real-time fluorescence kinetic curves are shown in Supplementary Figures S24–S26 and the polyacrylamide gel electrophoresis (PAGE) is given in Supplementary Figure S27. In Figure 2C, we set the toehold length to be either 7 or 9 nt with high activation efficiency to further compare the effect of toehold orientation on specificity. Among 3′ toeholds, MM-1 is the closest to the toehold, while MM-4 is the farthest from the toehold, because as the direction of the toehold changes, the distance of the mismatch site relative to the toehold changes accordingly. The results show that for the same mismatch, the closer to toehold, the more pronounced the inhibition of ERA-controlled Cas12a activation. As shown in Figure 2D and E, the DF of MM-1 is 1.2 (7 nt) and 1.0 (9 nt) at 5′ toehold, whereas it is 54.6 (7 nt) and 17.5 (9 nt) at 3′ toehold. However, the DF of MM-4 is 33.0 (7 nt) and 8.0 (9 nt) at 5′ toehold, whereas it is 2.1 (7 nt) and 2.0 (9 nt) at 3′ toehold. The same is true for the double-stranded activation mode, where ERA-controlled Cas12a increases the discrimination capacity by a maximum of 14.1-fold (DF 14.1:1.0) (Supplementary Figures S28–S31). The same results were obtained for the other set of sequences, with a more significant increase in specificity (up to 108.1-fold for single-strand activation and up to 169.1-fold for double-strand activation) (Supplementary Figures S32–S41). All these results show that the specificity discrimination law of TMSD is also applicable to ERA-controlled Cas12a, and we can flexibly adjust the detection specificity by changing the length and position of ERA, and there is almost no sequence dependence, which can be extended to any target.

Tolerance to mismatches can also be decreased by reducing the binding affinity of crRNAs and activators, and thus some crRNAs with secondary structures have been developed to improve the specificity of gene editing and sensing assays (9,10). As shown in Supplementary Scheme S1 and Supplementary Figure S42, the introduction of hairpin structure has led to a stepped regulation of Cas nuclease activity. However, unlike the nearly stepless regulation of the ERA toolbox, this strategy requires the design of corresponding crRNAs specifically for different regulatory gears, and ERA-controlled Cas12a also has significantly higher single-base discrimination specificity (DF enhancement up to 9.4-fold) (Supplementary Figure S43).

In summary, this external accessory is designed to be more mathematically and chemically predictable, with flexible and simple regulation methods and a wide range of control, making CRISPR/Cas safer for gene editing and therapeutics, and more accurate for molecular recognition and biosensing.

### Scalability based on DNA nanotechnology

**Figure 3.**
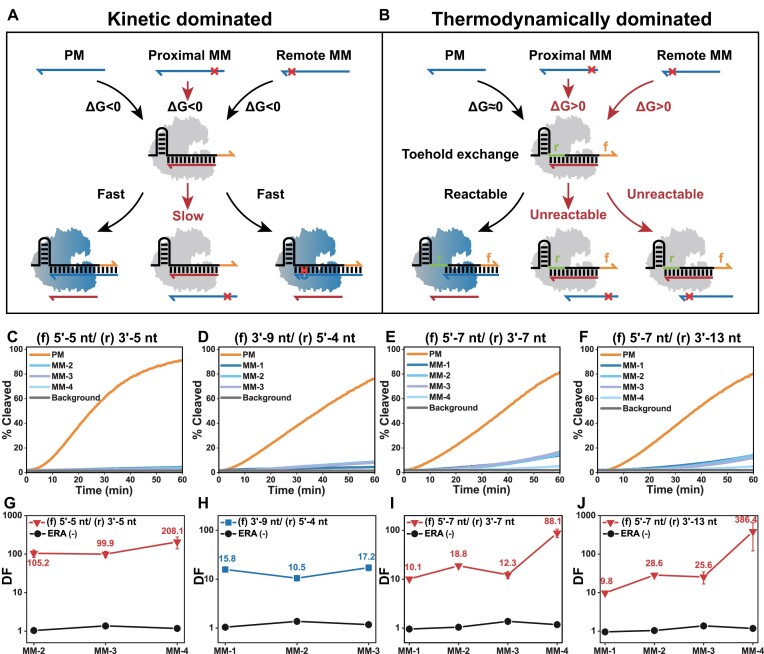
**(A)** When specificity is dominated by kinetics/TMSD, ERA-controlled Cas12a can hardly discriminate mismatches far from the toehold. **(B)** When specificity is dominated by thermodynamics/TE, ERA-controlled Cas12a discrimination is independent of mismatch position. The fluorescence kinetics **(C, D, E, F)** and discrimination factors **(G, H, I, J)** of TE-based ERA-controlled Cas12a identifying different mismatched activators. Orange represents the forward toehold exposed before TE reaction, and green represents the reverse toehold exposed after TE reaction. ERA (−) means no ERA is added. Error bars represented the standard deviation calculated from three independent experiments.

The single-base specificity of TMSD arises primarily from the apparent inhibition of mismatch reaction kinetics. Once the mismatch site is far away from the toehold that acts as a controller of TMSD kinetics, the reaction rate is virtually unaffected. Thus, while using TMSD as a switch to activate Cas12a greatly improves the single-base specificity of the CRISPR system, it has a narrow range of mismatch sensitivity. As shown in Figure 3A, toehold-proximal mismatch has a strong inhibitory effect on the TMSD reaction rate between activator with crRNA, which is almost absent when distal mismatches exist. (The toehold-proximal mismatched activator is discriminative even with Δ*G* < 0 just shows that the discrimination at this point is based on kinetics and has nothing to do with thermodynamics). Toehold exchange (TE) displacement allows essentially constant hydrogen bond formation and breakage before and after the reaction by introducing reverse toehold, so the net enthalpy change (Δ*H*) is small and can be fine-tuned to positive, negative or even zero by independently controlling forward (f) and reverse (r) toehold (34,60–62). Once ΔH is tuned close to zero, small thermodynamic differences caused by single base mismatch at any site will significantly alter the reaction yield. TE is an extension of TMSD to nucleic acid nanotechnology by introducing a reverse toehold controller to achieve more efficient fine-grained regulation of thermodynamics (34,61). Whether the same extension can be used for ERA-controlled CRISPR/Cas? We next designed the corresponding activator and ERA. In Figure 3B, the thermodynamics of the reaction is controlled by reverse toehold (green sequence labeled ‘r’), which causes Δ*G* > 0 with mismatched substrates, resulting in no reaction, regardless of whether it is proximal or distal.

Different from TMSD, the TE invasion strand is not fully complementary to the substrate strand due to a deletion at one end to produce the reverse toehold. But this deletion may inhibit CRISPR/Cas activation when applied to the activator. In Supplementary Figures S44–S45, we found that a certain activation efficiency was maintained with a deletion of up to 5 nt at either the 3′ end or the 5′ end of the activator when the crRNA length was constant (22 nt spacer), suggesting that ERA-controlled Cas12a is TE displacement feasible when the reverse toehold <5 nt. In Figure 3C and G, the discriminative power of all three mismatch sites increased significantly when we tried to exchange 5 nt 5′ toehold (f) for 5nt 3′ toehold (r) (compared with the DF of TMSD, the DF of MM-2 increased from 50.0 to 105.2, the DF of MM-3 increased from 29.8 to 99.9, and the DF of MM-4 increased from 31.6 to 208.1). Next, after reversing the direction and setting the 5′ toehold (r) to 4 nt (less intrusive on activation efficiency), we explored whether lengthening 3′ toehold (f) could improve activation efficiency (Supplementary Figure S46). In Figure 3D, H, we exchanged 9 nt 3′ toehold (f) for 4 nt 5′ toehold (r) to retain the activation efficiency. The DF of MM-1, which is the farthest from the forward 3′ toehold, still obtained 15.8 times the TMSD result (DF 15.8:1.0). In Supplementary Figure S47, we extend the end of the cRNA (35 nt spacer) and find that the deletion length allowed at the 5′ end of the activator increased to 19 nt, providing more room for reverse toehold regulation. As shown in Figure 3E, I, the 5′ toehold (f) of 7 nt was exchanged for the 3′ toehold (r) of 7 nt, and the DF of the farthest MM-1 was 8.4 times that of TMSD (DF 10.1:1.2). In Figure 3F, J, the 3′ toehold (r) is further extended to 13 nt, and the DF of MM-1 is also 8.1 times that of TMSD (DF 9.8:1.2). The corresponding heat map of DF versus time is shown in Supplementary Figures S48–S51. In Supplementary Figures S52–S53, the ERA toolkit is equally effective for the G-U mismatches (wobble), which is most difficult to identify.

The TE displacement takes Cas12a to a new level of mismatch discrimination from a thermodynamic control perspective, removes the high dependence on mismatch location and proves that ERA-controlled CRISPR/Cas is extremely compatible with highly programmable nucleic acid nanotechnology. The ERA strategy is highly scalable and evolutionary. It is foreseeable that using the same regularities, this work can be extended along the lines of DNA dynamic nanotechnology to achieve compatibility with a variety of traditional TMSD modulation tools, more and richer nucleic acid reaction networks can be tightly coupled to CRISPR/Cas via ERA for more sophisticated, complex and diverse functionality.

### Spatio-temporal continuity control in isothermal one-pot assay

**Figure 4.**
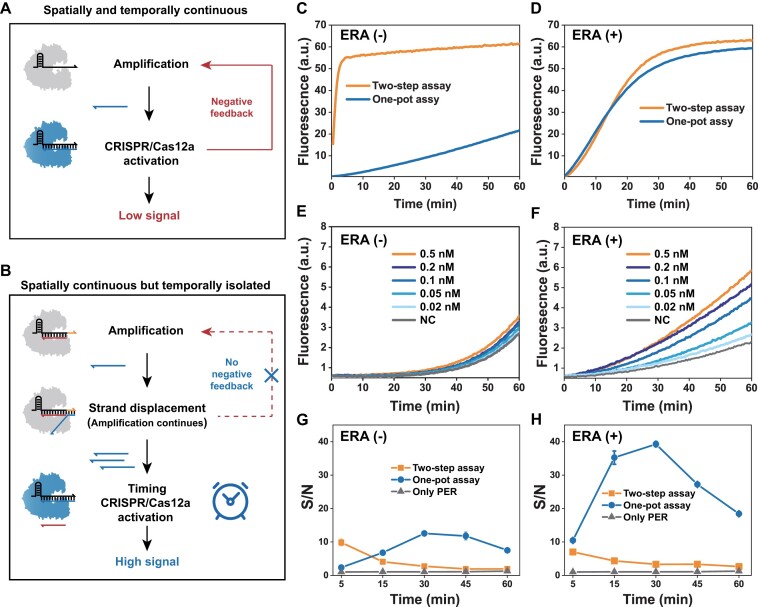
**(A)** In one-pot assay, the CRISPR/Cas system and the amplification system are spatially and temporally continuous, so the negative feedback effect leads to low detection efficiency. **(B)** The timing-activated ERA-controlled Cas12a system and the amplification system are spatially continuous but temporally isolated, independent of negative feedback effects to achieve high detection efficiency. The signal output efficiency of PER and CRISPR’s one-pot or two-step assay without **(C)** or with **(D)** ERA support. The sensitivity of one-step assays without **(E)** or with **(F)** ERA support. The signal-to-noise ratio of the one-pot or two-step assay without **(G)** or with **(H)** ERA support. Orange represents the toehold in the crRNA. ERA (−) means no ERA is added. Error bars represented the standard deviation calculated from three independent experiments.

Global epidemics have profoundly changed the landscape of nucleic acid detection, during which isothermal nucleic acid amplification (INA) followed by CRISPR/Cas detection strategies have shown great potential in molecular diagnostic applications. Systems based on this strategy, such as SHERLOCK (63), HOLMESv2 (64), DETECTR (65) and CRISDA (66) have been applied for highly sensitive viral and bacterial identification, as well as for rapid DNA methylation detection and SNP typing. However, separate nucleic acid pre-amplification procedure and multiple manual steps complicate the detection process, and transfer of amplification products may cause problems such as cross-contamination. Some subsequent studies have combined INA and CRISPR for a more portable and biosafe one-pot assay (67–69). But, unfortunately, the two programs, CRISPR/Cas and INA, are naturally incompatible in a one-pot cooker. Cas12a, which possesses strong trans-cleavage capability, cleaves not only Cas-reporters but also primers and templates. This incompatibility leads to inefficient detection and remains a challenge in clinical applications. As in Figure 4A, amplification and activation are continuous in space and time. In the early stage of amplification, the yield of activator is low but still sufficient to activate some of the Cas12a to disrupt primers and templates, providing negative feedback to the amplification program and ultimately achieving only a low signal output. Several studies have integrated INA and CRISPR into a compact, closed system in an attempt to spatially isolate these two incompatible programs. Although progress has been made, these approaches still require additional manipulation steps or complex microfluidic designs (70–73). Zhou *et al.* temporarily inactivated Cas12a by introducing PC-linker-modified protective oligos and then used light to activate the CRISPR program after the INA program was completed in the same tube (28). This spatially continuous but temporally isolated strategy is more convenient and controllable and may be a better solution, but it still requires tedious chemical modifications and external stimulation.

Here, we further explored whether the programmatic activation of CRISPR/Cas could be controlled by ERA without any modification or external stimulation based on the programmability of nucleic acid nanotechnology, thus automatically isolating the INA and CRISPR programs in the temporal dimension. As shown in Figure 4B, toehold can act as a controller for delayed activation of ERA-controlled Cas12a, allowing the indiscriminate trans-cleavage step to be misaligned with the INA program, buying time for the INA to amplify the activation strand and improving the efficiency of eventual activation of Cas12a. Taking primer exchange reaction (PER) as an example (schematic in Supplementary Scheme S2, conditions optimized in Supplementary Figure S54, sensitivity characterization in Supplementary Figure S55) (74,75). Without ERA, the PER system is disrupted by the *trans*-cleavage activity of Cas12a, so the signal of the one-pot assay is significantly lower than that of the conventional two-step assay (Figure 4C), and a target below 0.5 nM is already difficult to identify (Figure 4E, Supplementary Figure S56); with ERA, the *trans*-cleavage of Cas12a was delayed and misaligned with PER amplification program, and thus the signal of the one-step assay was significantly improved (Figure 4D) and the resolution for low concentration targets was also improved by nearly 10-fold (Figure 4F, Supplementary Figure S57), comparable to the conventional two-step assay (Supplementary Figure S58). In addition, the leakage of two-step assay always increased with time compared with the one-pot assay (Supplementary Figures S59, S60), probably because a certain degree of *trans*-cleavage suppressed the signal leakage of PER at the steps of template blocking, polymerase displacement or transient contact. As shown in Figure 4G, H, we find that the ERA-assisted one-pot assay could delay Cas12a activation and strive for amplification duration, while maintaining a certain degree of *trans*-cleavage activity to suppress PER leakage, and finally gain a better signal-to-noise ratio. Importantly, the ultimate goal of the ERA is not to improve the one-pot method, but rather to use it as an example to prove the great potential for such automated, finely programmable control of CRISPR/Cas. The amplification efficiency of PER is the main limiting factor of LOD, if further parameter optimization is required, we agree with the replacement by other INA methods to increase sensitivity (68,76,77) and these optimization methods are fully compatible with our ERA toolkit.

## CONCLUSION

For gene editing, the high efficiency of CRISPR is useful, but the uncontrollably high cutting efficiency also raises concerns about the safety of this technology. For the construction of nucleic acid-based molecular reaction networks, CRISPR destroys the entire system immediately on activation, so the systems constructed have been designed with CRISPR as the last step, with some limitations. For gene therapy or delivery as drugs, slow and sustained releasing Cas nuclease is necessary. If the performance of Cas nuclease can be more freely regulated, these challenges will be solved more direct and easy.

Here, we present a simple yet powerful strategy for controlling multi-performance of CRISPR/Cas12a to meet the more diverse and nuanced needs of rapidly evolving and finely divided field of molecular biology. Eliminating the need for modifications to Cas proteins or crRNA components, the strategy leverages the powerful programmability of nucleic acid nanotechnology to enable fine-grained and predictable control of multiple CRISPR/Cas12a properties based on a customizable ERA toolbox. Through systematic study of its thermodynamic and kinetic principles, we found that unlike the previous crude ON/OFF control, the toehold length, direction, position, ratio and mismatched position of ERA can act as multidimensional controllers of Cas12a performance, complementing each other to achieve almost stepless regulation of single- and double-stranded activation. And we confirmed that this is an integrated control that can act on multiple properties such as activity, specificity, speed, compatibility, programmability, sensitivity etc. Especially, the great increase in specificity is likely to significantly improve the safety issues of gene editing. This strategy is not only more versatile, cost-effective and biosafe, but also has the potential to be compatible with existing Cas engineering or crRNA modification strategies for more diverse combinatorial control. Even for CRISPR families targeting RNA such as Cas13, the appropriate external DNA accessory (EDA) toolkit can be added to achieve similar results. CRISPR technology has led one pace of revolution in molecular biomedicine, and we hope that ERA can deepen its integration with nucleic acid nanotechnology to drive this revolution into a ‘new era’.

## Supplementary Material

gkad748_Supplemental_File

## Data Availability

All data supporting the findings of this study are available within the article and its supplementary information or will be made available from the authors upon request.
