# Supplementary material for: Unmodificated stepless regulation of CRISPR/Cas12a multi-performance
Source: Nucleic Acids Res. 2023 Sep 27;51(19):10795–807. doi: 10.1093/nar/gkad748 (PMC10602922; doi:10.1093/nar/gkad748)
Supplement: gkad748_Supplemental_File [file gkad748_supplemental_file.pdf]

Supporting information

Unmodificated Stepless Regulation of CRISPR/Cas12a Multi-Performance

Rong Zhao[1,†], Wang Luo[1,†], You Wu[1], Li Zhang[1], Xin Liu[1], Junjie Li[1], Yujun Yang[1], Li Wang[2], Luojia Wang[1], Xiaole Han[1], Zhongzhong Wang[1], Jianhong Zhang[1], Ke Lv[3,*], Tingmei Chen[1,*], Guoming Xie[1,*]

[1] Key Laboratory of Clinical Laboratory Diagnostics (Chinese Ministry of Education), College of Laboratory Medicine, Chongqing Medical Laboratory Microfluidics and SPRi Engineering Research Center, Chongqing Medical University, Chongqing, 400016, PR China

[2] The Center for Clinical Molecular Medical Detection, The First Affiliated Hospital of Chongqing Medical University, Chongqing 400016, PR Chinaa

[3] Department of Neurosurgery, The First Affiliated Hospital of Chongqing Medical University Chongqing, 400016, PR China

[*] To whom correspondence should be addressed. Tel: +86 23 68485240; Fax: +86 2368485239; Email: guomingxie@cqmu.edu.cn

Correspondence may also be addressed to Tingmei Chen. Email: tingmeichen@cqmu.edu.cn

Correspondence may also be addressed to Ke Lv. Email: luke@hospital.cqmu.edu.cn

[†] R.Z and W.L contributed equally to this work, they wish it to be regarded as Joint First Authors.

Table of Contents

Supplementary Tables 5

Table S1 | Customized nucleic acids sequence 5

Table S2 | Gibbs free energy changes of different ERA-crRNA complexes 10

Table S3 | Enzyme kinetic parameters of Cas12a under ERA control 11

Table S4 | Gibbs free energy changes of different ERA-crRNA-β complexes 12

Table S5 | Enzyme kinetic parameters of Cas12a under different ERA-β control 13

Table S6 | Gibbs free energy changes of different ERA(mismatched)-crRNA complexes 14

Table S7 | Gibbs free energy changes when both ERA and activator are regarded as RNA 15

Table S8 | Gibbs free energy changes when both ERA and activator are regarded as DNA 16

Supplementary Schemes 17

Scheme S1 | Introduction of different hairpin structures in crRNA 17

Scheme S2 | Schematic diagram of PER 18

Scheme S3 | The sequence in Figure 1A-1D 19

Scheme S4 | The sequence in Figure S11-S12 20

Scheme S5 | The sequence in Figure S11-S12 21

Scheme S6 | The sequence in Figure 1E-1I 22

Scheme S7 | The sequence in Figure S19-S22..23

Scheme S8 | The sequence in Figure 2C-2E and Figure S8 24

Scheme S9 | The sequence in Figure S36 25

Scheme S10 | The sequence in Figure S40 26

Scheme S11 | The sequence in Figure 3 27

Scheme S12 | The sequence in Figure 3 28

Scheme S13 | The sequence in Figure 3 29

Scheme S14 | The sequence in Figure S28-S29 30

Scheme S15 | The sequence in Figure S28-S29 31

Scheme S16 | The sequence in Figure S28-S29 32

Scheme S17 | The sequence in Scheme S1 and Figure S42-43 33

Scheme S18 | The sequence in Figure S44-S45 34

Scheme S19 | The sequence in Figure S44-S45 35

Scheme S20 | The sequence in Figure S44-S45 36

Scheme S21 | The sequence in Figure S52 37

Supplementary Figures 38

Multidimensional control of activation velocity and cleavage activity 38

Figure S1 | Effect of 5' toehold length (ssDNA-activated) 38

Figure S2 | Effect of 5' toehold length at low target concentrations (ssDNA-activated) 39

Figure S3 | Effect of 3' toehold length (ssDNA-activated) 40

Figure S4 | Effect of addition ratio of 5' toehold-4 nt ERA on ssDNA-PM-activated Cas12a 41

Figure S5 | Effect of addition ratio of 5' toehold-4 nt ERA on ssDNA-MM-activated Cas12a ... 42

Figure S6 | Effect of 5' toehold-β length (ssDNA-activated) 43

Figure S7 | Effect of 3' toehold-β length (ssDNA-activated) 44

Figure S8 | k_{cat}/K_M at different toehold-β lengths and directions (ssDNA-activated) 45

Figure S9 | Effect of addition ratio of 5' toehold-7 nt ERA-β on ssDNA-PM-activated Cas12a .46

Figure S10 | Effect of addition ratio of 5' toehold-7 nt ERA-β on ssDNA-MM-activated Cas12a

.... 47

Figure S11 | Fluorescence versus cleaved reporter concentration calibration 48

Figure S12 | Effect of 5' toehold length (dsDNA-activated) 49

Figure S13 | Effect of 3' toehold length (dsDNA-activated) 50

Figure S14 | Effect of 5' toehold-β length (dsDNA-activated) 51

Figure S15 | Effect of 3' toehold-β length (dsDNA-activated) 52

Figure S16 | Effect of ERA (3' toehold-6 nt) mismatch sites (ssDNA-activated) 53

Figure S17 | Effect of ERA (3' toehold-7 nt) mismatch sites (ssDNA-activated) 54

Figure S18 | Effect of ERA (5' toehold-4 nt) mismatch sites (ssDNA-activated) 55

Figure S19 | Effect of ERA (5' toehold-7 nt) mismatch sites (ssDNA-activated) 56

Figure S20 | Effect of ERA (3' toehold-6 nt) mismatch sites (dsDNA-activated) 57

Figure S21 | Effect of ERA (3' toehold-7 nt) mismatch sites (dsDNA-activated) 58

Figure S22 | Effect of ERA (5' toehold-4 nt) mismatch sites (dsDNA-activated) 59

Figure S23 | Effect of ERA (5' toehold-7 nt) mismatch sites (dsDNA-activated) 60

Kinetics-driven control of activation specificity 61

Figure S24 | Specificity of ssDNA-activated Cas12a without ERA 61

Figure S25 | Effect of 5' toehold length (ssDNA-activated) 62

Figure S26 | Effect of 3' toehold length (ssDNA-activated) 63

Figure S27 | PAGE characterization of TMSD between ssDNA-activator and ERA/crRNA 64

Figure S28 | Specificity of dsDNA-activated Cas12a without ERA 65

Figure S29 | Effect of toehold (7 nt) direction (dsDNA-activated) 66
Figure S30 | Effect of toehold (9 nt) direction (dsDNA-activated) 67
Figure S31 | Effect of toehold length and direction (dsDNA-activated) 68
Figure S32 | Specificity of ssDNA-activated Cas12a without ERA-β 69
Figure S33 | Effect of 5' toehold-β length (ssDNA-activated) 70
Figure S34 | DF-time heat map of ERA-β-Cas12a on ssDNA activators 71
Figure S35 | Effect of 3' toehold-β length (ssDNA-activated) 72
Figure S36 | Effect of toehold-β length and direction (dsDNA-activated) 73
Figure S37 | Effect of toehold-β length and direction (ssDNA-activated) 74
Figure S38 | Specificity of dsDNA-activated Cas12a without ERA-β 75
Figure S39 | Effect of toehold-β (7 nt) direction (dsDNA-activated) 76
Figure S40 | Effect of toehold-β (9 nt) direction (dsDNA-activated) 77
Figure S41 | Effect of toehold-β length and direction (dsDNA-activated) 78
Figure S42 | Effect of the toehold length on hs-crRNA-Cas12a activity 79
Figure S43 | Comparison of the specifity of hairpin-controlled with ERA-controlled Cas12a ... 80
Scalability based on DNA nanotechnology..81
Figure S44 | Effect of 3' end deletion of ssDNA activator on cleavage rate (spacer=22 nt) 81
Figure S45 | Effect of 5' end deletion of ssDNA activator on cleavage rate (spacer=22 nt) 82
Figure S46 | Screening for optimal 3' toehold (r) length 83
Figure S47 | Effect of activator -δ 3' end deletion on cleavage rate (spacer=35 nt) 84
Figure S48 | DF-time heat map of (f)5'-5 nt/(r) 5'-5 nt TE-based ERA-Cas12a (spacer=22 nt) .. 85
Figure S49 | DF-time heat map of (f)3'-4 nt/(r)5'-9 nt TE-based ERA-Cas12a (spacer=22 nt) ... 86
Figure S50 | DF-time heat map of (f)5'-7 nt/(r)3'-7 nt TE-based ERA-Cas12a (spacer=29 nt) ... 87
Figure S51 | DF-time heat map of (f)5'-7 nt/(r)3'-13 nt TE-based ERA-Cas12a (spacer=35 nt) . 88
Figure S52 | Discrimination of G-U mismatches by ERA-Cas12a 89
Figure S53 | Discrimination of GU mismatches by ERA-Cas12a-β 90
Spatio-temporal continuity control in isothermal one-pot assay 91
Figure S54 | Optimized experimental conditions for PER reaction 91
Figure S55 | Sensitivity of PER alone 92
Figure S56 | Sensitivity of one-pot assay without ERAsupport 93
Figure S57 | Sensitivity of one-pot assay with ERA support 94
Figure S58 | Sensitivity of traditonal two-step assay 95
Figure S59 | Leakage-time comparison of one-pot and two-step assay without ERA support 96
Figure S60 | Leakage-time comparison of one-pot and two-step assay with ERA support 97

Supplementary Tables

Table S1. Customized nucleic acids sequence.

Name	Sequence (5'→3')
crRNA	UAAUUUCUACUAAGUGUAGAUUAAGUUCAAGGUGUGCGCAAUG
5' toehold-15 nt	AUUCAAG
5' toehold-11 nt	AUUCAAGUUCC
5' toehold-10 nt	AUUCAAGUUCCA
5' toehold-9 nt	AUUCAAGUUCCAC
5' toehold-8 nt	AUUCAAGUUCCACA
5' toehold-7 nt	AUUCAAGUUCCACAC
5' toehold-6 nt	AUUCAAGUUCCACACG
5' toehold-5 nt<br>(f) 5' toehold-5 nt/(r) 3' toehold-5 nt	AUUCAAGUUCCACACGC
5' toehold-4 nt	AUUCAAGUUCCACACGCG
5' toehold-3 nt	AUUCAAGUUCCACACGCGU
5' toehold-2 nt	AUUCAAGUUCCACACGCGUU
5' toehold-1 nt	AUUCAAGUUCCACACGCGUUA
3' toehold-15 nt	GAACUUA
3' toehold-11 nt	CCUUGAACUUA
3' toehold-10 nt	ACCUUGAACUUA
3' toehold-9 nt<br>(f)3' toehold-9 nt/(r)5' toehold-4 nt	CACCUUGAACUUA
3' toehold-8nt	ACACCUUGAACUUA
3' toehold-7nt<br>(f) 3' toehold-7 nt/(r) 5' toehold-4 nt	CACACCUUGAACUUA
3' toehold-6 nt	GCACACCUUGAACUUA
3' toehold-5 nt	CGCACACCUUGAACUUA
3' toehold-4 nt<br>(f) 3' toehold-4 nt/(r) 5' toehold-4 nt	GCGCACACCUUGAACUUA
3' toehold-3 nt	UGCGCACACCUUGAACUUA
3' toehold-2 nt	UUGCGCACACCUUGAACUUA
3' toehold-1 nt	AUUGCGCACACCUUGAACUUA
toehold-0 nt	CAUUGCGCACACCUUGAACUUA
3' toehold-6 nt/3 nt	GCCCACCUUGAACUUA
3' toehold-6 nt/6 nt	GCACAGCUUGAACUUA
3' toehold-6 nt/9 nt	GCACACCUCGAACUUA
3' toehold-6 nt/12 nt	GCACACCUUGACCUUA
3' toehold-6 nt/15 nt	GCACACCUUGAACUCA

3' toehold-7 nt/1 nt	GACACCUUGAACUUA
3' toehold-7 nt/4 nt	CACCCCUUGAACUUA
3' toehold-7 nt/7 nt	CACACCCUGAACUUA
3' toehold-7 nt/10 nt	CACACCUUGCACUUA
3' toehold-7 nt/14 nt	CACACCUUGAACUCA
5' toehold-6 nt/3 nt	CAUUGCGCACACCUCGAA
5' toehold-6 nt/6 nt	CAUUGCGCACAGCUUGAA
5' toehold-6 nt/9 nt	CAUUGCGCCCACCUUGAA
5' toehold-6 nt/12 nt	CAUUGGGCACACCUUGAA
5' toehold-6 nt/15 nt	CACUGCGCACACCUUGAA
5'toehold-7 nt/1 nt	CAUUGCGCACACCUC
5' toehold-7 nt/4 nt	CAUUGCGCACAGCUU
5' toehold-7 nt/7 nt	CAUUGCGCCCACCUU
5' toehold-7 nt/10 nt	CAUUGGGCACACCUU
5' toehold-7 nt/14 nt	CCUUGCGCACACCUU
PM	CATTGCGCACACCTTGAACTTA
MM-1	CATTCCGCACACCTTGAACTTA
MM-2	CATTGCGCCCACCTTGAACTTA
MM-3	CATTGCGCACACCCTGAACTTA
MM-4	CATTGCGCACACCTTGAAGTTA
MM-G	CATTGCGCACGCCTTGAACTTA
ds-TS-PM	CGATCATTGCGCACACCTTGAACTTATAAACGAT
ds-NTS-PM	ATCGTTTATAAGTTCAAGGTGTGCGCAATGATCG
ds-TS-MM-1	CGATCATTCCGCACACCTTGAACTTATAAACGAT
ds-NTS-MM-1	ATCGTTTATAAGTTCAAGGTGTGCGGAATGATCG
ds-TS-MM-2	CGATCATTGCGCCCACCTTGAACTTATAAACGAT
ds-NTS-MM-2	ATCGTTTATAAGTTCAAGGTGGGCGCAATGATCG
ds-TS-MM-3	CGATCATTGCGCACACCCTGAACTTATAAACGAT
ds-NTS-MM-3	ATCGTTTATAAGTTCAGGGTGTGCGCAATGATCG
ds-TS-MM-4	CGATCATTGCGCACACCTTGAAGTTATAAACGAT
ds-NTS-MM-4	ATCGTTTATAACTTCAAGGTGTGCGCAATGATCG
ds-TS-MM-G	CGATCATTGCGCACGCCTTGAACTTATAAACGAT
ds-NTS-MM-G	ATCGTTTATAAGTTCAAGGCGTGCGCAATGATCG
crRNA-β	UAAUUUCUACUAAGUGUAGAUUGCGAGUAACGUCCA CUAUCUC
5' toehold-15 nt-β	GAGAUAG
5' toehold-11 nt-β	GAGAUAGUGGA
5' toehold-10 nt-β	GAGAUAGUGGAC
5' toehold-9 nt-β	GAGAUAGUGGACG
5' toehold-8 nt-β	GAGAUAGUGGACGU
5' toehold-7 nt-β	GAGAUAGUGGACGUU
5' toehold-6 nt-β	GAGAUAGUGGACGUUA
5' toehold-5 nt-β	GAGAUAGUGGACGUUAC
5' toehold-4 nt-β	GAGAUAGUGGACGUUACU

5' toehold-3 nt-β	GAGAUAGUGGACGUUACUC
5' toehold-2 nt-β	GAGAUAGUGGACGUUACUCG
5' toehold-1 nt-β	GAGAUAGUGGACGUUACUCGC
3' toehold-15 nt-β	ACUCGCA
3' toehold-11 nt-β	CGUUACUCGCA
3' toehold-10 nt-β	ACGUUACUCGCA
3' toehold-9 nt-β	GACGUUACUCGCA
3' toehold-8 nt-β	GGACGUUACUCGCA
3' toehold-7 nt-β	UGGACGUUACUCGCA
3' toehold-6 nt-β	GUGGACGUUACUCGCA
3' toehold-5 nt-β	AGUGGACGUUACUCGCA
3' toehold-4 nt-β	UAGUGGACGUUACUCGCA
3' toehold-3 nt-β	AUAGUGGACGUUACUCGCA
3' toehold-2 nt-β	GAUAGUGGACGUUACUCGCA
3' toehold-1 nt-β	AGAUAGUGGACGUUACUCGCA
toehold-0 nt-β	GAGAUAGUGGACGUUACUCGCA
PM-β	GAGATAGTGGACGTTACTCGCA
MM-1-β	GAGAAAGTGGACGTTACTCGCA
MM-2-β	GAGATAGTAGACGTTACTCGCA
MM-3-β	GAGATAGTGGACGATACTCGCA
MM-4-β	GAGATAGTGGACGTTACTAGCA
MM-G-β	GAGATAGTGGGCGTTACTCGCA
ds-TS-PM-β	CGATGAGATAGTGGACGTTACTCGCATAAACGAT
ds-NTS-PM-β	ATCGTTTATGCGAGTAACGTCCACTATCTCATCG
ds-TS-MM-1-β	CGATGAGAAAGTGGACGTTACTCGCATAAACGAT
ds-NTS-MM-1-β	ATCGTTTATGCGAGTAACGTCCACTTTCTCATCG
ds-TS-MM-2-β	CGATGAGATAGTAGACGTTACTCGCATAAACGAT
ds-NTS-MM-2-β	ATCGTTTATGCGAGTAACGTCTACTATCTCATCG
ds-TS-MM-3-β	CGATGAGATAGTGGACGATACTCGCATAAACGAT
ds-NTS-MM-3-β	ATCGTTTATGCGAGTATCGTCCACTATCTCATCG
ds-TS-MM-4-β	CGATGAGATAGTGGACGTTACTAGCATAAACGAT
ds-NTS-MM-4-β	ATCGTTTATGCTAGTAACGTCCACTATCTCATCG
ds-TS-MM-G-β	CGATGAGATAGTGGGCGTTACTCGCATAAACGAT
ds-NTS-MM-G-β	ATCGTTTATGCGAGTAACGCCCACTATCTCATCG
3' del-2 nt	CATTGCGCACACCTTGAACT
3' del-4 nt<br>3' del-PM	CATTGCGCACACCTTGAA
3' del-MM-1	CATTCCGCACACCTTGAA
3' del-MM-2	CATTGCGCCCACCTTGAA
3' del-MM-3	CATTGCGCACACCCTGAA
3' del-4 nt	CATTGCGCACACCTTGAA
3' del-5 nt	CATTGCGCACACCTTGA
3' del-6 nt	CATTGCGCACACCTTG
3' del-8 nt	CATTGCGCACACCT

3' del-10 nt	CATTGCGCACAC
3' del-12 nt	CATTGCGCAC
3' del-14 nt	CATTGCGC
5' del-2 nt	TTGCGCACACCTTGAACTTA
5' del-4 nt	GCGCACACCTTGAACTTA
5' del-5 nt<br>5' del-PM	CGCACACCTTGAACTTA
5' del-MM-2	CGCCCACCTTGAACTTA
5' del-MM-3	CGCACACCCTGAACTTA
5' del-MM-4	CGCACACCTTGAAGTTA
5' del-6 nt	GCACACCTTGAACTTA
5' del-8 nt	ACACCTTGAACTTA
5' del-10 nt	ACCTTGAACTTA
5' del-12 nt	CTTGAACTTA
5' del-14 nt	TGAACTTA
crRNA-γ	UAAUUUCUACUAAGUGUAGAUUAAGUUCAAGGUGU<br>G CGCAAUGUUAUUAU
crRNA-δ	UAAUUUCUACUAAGUGUAGAUUAAGUUCAAGGUGU<br>GCGCAAUGUUAUUAUUAUUAU
(f) 5' toehold-7 nt/(r) 3' toehold-7 nt	AUAAUAACAUUGCGCACACCUU
(f) 5' toehold-7 nt/(r) 3' toehold-13 nt	AUAAUAAUAAUAACAUUGCGCACACCUU
PM-δ	ATAATAATAATAACATTGCGCACACCTTGAACTTA
5' del-2 nt-δ	AATAATAATAACATTGCGCACACCTTGAACTTA
5' del-6 nt-δ	ATAATAACATTGCGCACACCTTGAACTTA
5' del-10 nt-δ	TAACATTGCGCACACCTTGAACTTA
5' del-12 nt-δ	ACATTGCGCACACCTTGAACTTA
5' del-14 nt-δ	ATTGCGCACACCTTGAACTTA
5' del-16 nt-δ	TGCGCACACCTTGAACTTA
5' del-18 nt-δ	CGCACACCTTGAACTTA
5' del -19 nt-δ	GCACACCTTGAACTTA
5' del-20 nt-δ	CACACCTTGAACTTA
hs-crRNA-toehold/9 nt	UAAUUUCUACUAAGUGUAGAUUAAGUUCAAGGUGU<br>GCGCAAUGAACACAUUGCGCACACC
hs-crRNA-toehold/7 nt	UAAUUUCUACUAAGUGUAGAUUAAGUUCAAGGUGU<br>GCGCAAUGAACACAUUGCGCACACCUU
hs-crRNA-toehold/5 nt	UAAUUUCUACUAAGUGUAGAUUAAGUUCAAGGUGU<br>GCGCAAUGAACACAUUGCGCACACCUUGA
hs-crRNA-toehold/3 nt	UAAUUUCUACUAAGUGUAGAUUAAGUUCAAGGUGU<br>GCGCAAUGAACACAUUGCGCACACCUUGAAC
hs-crRNA-toehold/0 nt	UAAUUUCUACUAAGUGUAGAUUAAGUUCAAGGUGU<br>GCGCAAUGAACACAUUGCGCACACCUUGAACUUA
hairpin-template	CTCTCTTATTGGGCCTTTTGGCCCAATAAGAGAGTTGA<br>ATTTAGTAGCTTATCA-***Inverted dT***

protector	TCAACATCAGTCTGATAAGCTACTAAATTCAA-***Inverted dT***
MiRNA-21	UAGCUUAUCAGACUGAUGUUGA
primer	TACTAAATTC
probe-F	***HEX***-ACTAAATTCAACTC
probe-Q	AATAAGAGAGTTGAATTTAGT-***BHQ1***
crRNA-ε	UAAUUUCUACUAAGUGUAGAUAAUAAGAGAGUUGAAUUUAGUA
5' toehold-9 nt-ε	UACUAAAUUCAAC
5' toehold-7 nt-ε	UACUAAAUUCAACUC
5' toehold-5 nt-ε	UACUAAAUUCAACUCUC
5' toehold-3 nt-ε	UACUAAAUUCAACUCUCUU
Cas-reporter	***HEX***-TTATT-***BHQ1***

Note. There are five sets of nucleic acid sequences that match the corresponding CRISPR/Cas12a system. For simplicity, the α tag of the first set of sequences appearing in the text is omitted, and the other four sets of sequences are labeled with the Greek letters β, γ, δ, and ε at the end of the sequence name, respectively. The mutant bases are labeled by underlining and the chemical modifications are labeled in bold italic font.

Table S2. Gibbs free energy changes of different ERA-crRNA complexes.

	ΔG (kcal/mol)	
Toehold length	5' toehold	3' toehold
15 nt	-20.27	-17.99
11 nt	-30.70	-27.04
10 nt	-33.84	-29.16
9 nt	-37.57	-32.01
8 nt	-39.40	-34.14
7 nt	-40.24	-36.83
6 nt	-44.74	-41.95
5 nt	-44.84	-43.39
4 nt	-46.59	-48.53
3 nt	-49.78	-48.17
2 nt	-51.61	-50.33
1 nt	-52.65	-51.93
without toehold	-53.92	-53.92

The shorter the toehold, i.e. the longer the complementary region of the ERA, the more negative the Gibbs free energy and the more difficult it is for the ERA to be replaced.

Table S3. Enzyme kinetic parameters of Cas12a under different ERA control.

Toehold direction and length	k_{cat} (1/s)	K_M (M)	k_{cat}/K_M (1/Ms)
without ERA	920	6.23×10^{-7}	14.7642×10^{8}
5' toehold-15nt	1060	5.93×10^{-7}	17.8898×10^{8}
5' toehold-11nt	918	5.97×10^{-7}	15.3758×10^{8}
5' toehold-10nt	716	6.03×10^{-7}	11.8696×10^{8}
5' toehold-9nt	825.5	6.00×10^{-7}	13.7598×10^{8}
5' toehold-8nt	524.5	6.09×10^{-7}	8.6095×10^{8}
5' toehold-7nt	424.5	6.11×10^{-7}	6.9379×10^{8}
5' toehold-6nt	171	6.19×10^{-7}	2.7617×10^{8}
5' toehold-5nt	14	6.23×10^{-7}	0.2245×10^{8}
5' toehold-4nt	9.8	6.24×10^{-7}	0.1577×10^{8}
5' toehold-3nt	2.6	6.25×10^{-7}	0.0422×10^{8}
5' toehold-2nt	1	6.25×10^{-7}	0.0161×10^{8}
5' toehold-1nt	1.7	6.25×10^{-7}	0.0272×10^{8}
3' toehold-15nt	1063	5.92×10^{-7}	17.9507×10^{8}
3' toehold-11nt	814	6.00×10^{-7}	13.5582×10^{8}
3' toehold-10nt	803	6.01×10^{-7}	13.3675×10^{8}
3' toehold-9nt	525	6.09×10^{-7}	8.6153×10^{8}
3' toehold-8nt	615	6.06×10^{-7}	10.1468×10^{8}
3' toehold-7nt	23.6	6.21×10^{-7}	0.3801×10^{8}
3' toehold-6nt	2.32	6.25×10^{-7}	0.0372×10^{8}
3' toehold-5nt	3.56	6.24×10^{-7}	0.057×10^{8}
3' toehold-4nt	4.06	6.24×10^{-7}	0.065×10^{8}
3' toehold-3nt	4.05	6.23×10^{-7}	0.065×10^{8}
3' toehold-2nt	0.63	6.25×10^{-7}	0.0102×10^{8}
3' toehold-1nt	0.93	6.25×10^{-7}	0.0149×10^{8}
without toehold	0.8	6.25×10^{-7}	0.0132×10^{8}

Independent of direction, the longer the toehold, the greater the k_{cat}/K_M and the higher the activity of Cas12a.

Table S4. Gibbs free energy changes of different ERA-crRNA-β complexes.

	ΔG (kcal/mol)	
Toehold length	5' toehold	3' toehold
15 nt	-21.57	-22.38
11 nt	-30.54	-30.77
10 nt	-33.30	-32.35
9 nt	-36.22	-36.28
8 nt	-37.97	-40.93
7 nt	-39.01	-41.50
6 nt	-40.61	-45.40
5 nt	-43.80	-46.77
4 nt	-45.71	-48.13
3 nt	-49.01	-49.20
2 nt	-51.67	-53.58
1 nt	-55.44	-54.41
without toehold	-57.30	-57.30

As with the previous set of sequences, the shorter the toehold, the more negative the Gibbs free energy and the more difficult the ERA-β is to be replaced.

Table S5. Enzyme kinetic parameters of Cas12a under different ERA-β control.

Toehold direction and length	k_{cat} (1/s)	K_M (M)	k_{cat}/K_M (1/Ms)
without ERA	266.5	6.17×10^{-7}	4.316×10^{8}
5' toehold-15 nt-β	125.5	6.21×10^{-7}	2.0203×10^{8}
5' toehold-11 nt-β	98	6.21×10^{-7}	1.5784×10^{8}
5' toehold-10 nt-β	129.5	6.21×10^{-7}	2.0864×10^{8}
5' toehold-9 nt-β	148.5	6.21×10^{-7}	2.3908×10^{8}
5' toehold-8 nt-β	99	6.22×10^{-7}	1.5928×10^{8}
5' toehold-7 nt-β	80.5	6.22×10^{-7}	1.2935×10^{8}
5' toehold-6 nt-β	73	6.23×10^{-7}	1.1713×10^{8}
5' toehold-5 nt-β	33.4	6.2×10^{-7}	0.5387×10^{8}
5' toehold-4 nt-β	23.8	6.22×10^{-7}	0.3823×10^{8}
5' toehold-3 nt-β	7.1	6.24×10^{-7}	0.1133×10^{8}
5' toehold-2 nt-β	3.29	6.24×10^{-7}	0.0527×10^{8}
5' toehold-1 nt-β	2.54	6.25×10^{-7}	0.0407×10^{8}
3' toehold-15 nt-β	161.5	6.21×10^{-7}	2.602×10^{8}
3' toehold-11 nt-β	52.5	6.22×10^{-7}	0.8437×10^{8}
3' toehold-10 nt-β	56.5	6.24×10^{-7}	0.9058×10^{8}
3' toehold-9 nt-β	35.4	6.24×10^{-7}	0.5666×10^{8}
3' toehold-8 nt-β	5.03	6.24×10^{-7}	0.0805×10^{8}
3' toehold-7 nt-β	5.24	6.24×10^{-7}	0.084×10^{8}
3' toehold-6 nt-β	4.04	6.24×10^{-7}	0.0647×10^{8}
3' toehold-5 nt-β	5.93	6.24×10^{-7}	0.095×10^{8}
3' toehold-4 nt-β	0.83	6.25×10^{-7}	0.0133×10^{8}
3' toehold-3 nt-β	2.73	6.24×10^{-7}	0.0437×10^{8}
3' toehold-2 nt-β	1.50	6.24×10^{-7}	0.024×10^{8}
3' toehold-1 nt-β	6.02	6.24×10^{-7}	0.0965×10^{8}
without toehold	0.78	6.25×10^{-7}	0.0063×10^{8}

As with the previous set of sequences, the longer the toehold, the larger the k_{cat}/K_M and the higher the activity of Cas12a, regardless of direction.

Table S6. Gibbs free energy changes of different ERA-crRNA complexes.

	Toehold direction and length			
ERA type	3' toheold-6 nt	3' toheold-7 nt	5' toheold-4 nt	5' toheold-7 nt
Farthest mismatch	-40.33	-35.2	-42.24	-37.02
Far mismatch	-37.33	-31.97	-42.86	-36.51
Middle mismatch	-37.46	-32.33	-41.45	-35.09
Close mismatch	-37.57	-31.68	-42.21	-35.86
Closest mismatch	-36.8	-34.89	-42.1	-38.61
Match	-41.95	-36.83	-46.59	-40.24

All eliminated mismatches have a large negative contribution to the ΔG of the reaction. Our hypothesis is that the slower acceleration caused by late mismatch elimination relative to early mismatch elimination is due to a hidden thermodynamic drive rather than the difference in free energy changes.

Table S7. Gibbs free energy changes before and after the reaction when all complexes are regarded as RNA. (Although the activator-crRNA complex is actually DNA-RNA, NUPACK cannot yet be calculated, so it is approximated as RNA-RNA. And the approximation to DNA-DNA results in Table S8.)

	ΔΔG=ΔG (crRNA-activator) - ΔG(crRNA-ERA)			
ERA type	3' toheold-6 nt	3' toheold-7 nt	5' toheold-4 nt	5' toheold-7 nt
Farthest mismatch	-13.59	-18.72	-11.68	-16.9
Far mismatch	-16.57	-21.95	-11.06	-17.41
Middle mismatch	-16.46	-21.59	-12.47	-18.83
Close mismatch	-16.35	-22.24	-11.71	-18.06
Closest mismatch	-17.12	-19.03	-11.82	-15.31
Match	-11.97	-17.09	-7.33	-13

The binding of activator to Cas12a and the conformational change of Cas12a is a two-step reactions: the first step is only related to the difference between crRNA-ERA and crRNA-activator, while the second step is only related to the conformational change of the protein itself (with or without ERA, at this point the ternary complex is Cas12a-crRNA-activator). Thus taking only the first step into account, ΔΔG is more negative in the presence of mismatch on the ERA, suggesting that it would be easier for the activator to displace the mismatched ERA, which is consistent with the thermodynamic law of strand displacement.

Table S8. Gibbs free energy changes before and after the reaction when all complexes are regarded as DNA.

	ΔΔG=ΔG (crRNA-activator) - ΔG(crRNA-ERA)			
ERA type	3' toheold-6 nt	3' toheold-7 nt	5' toheold-4 nt	5' toheold-7 nt
Farthest mismatch	-12.97	-14.4	-8.62	-10.44
Far mismatch	-15.15	-16.1	-8.79	-11.88
Middle mismatch	-15.75	-16.34	-10.5	-14.8
Close mismatch	-12.61	-16.96	-7.58	-13.09
Closest mismatch	-15.15	-13.7	-10.72	-11.71
Match	-10.39	-11.82	-5.36	-9.66

The results are the same as in Table S7, and both DNA and RNA complexes fit the pattern.

Supplementary Schemes

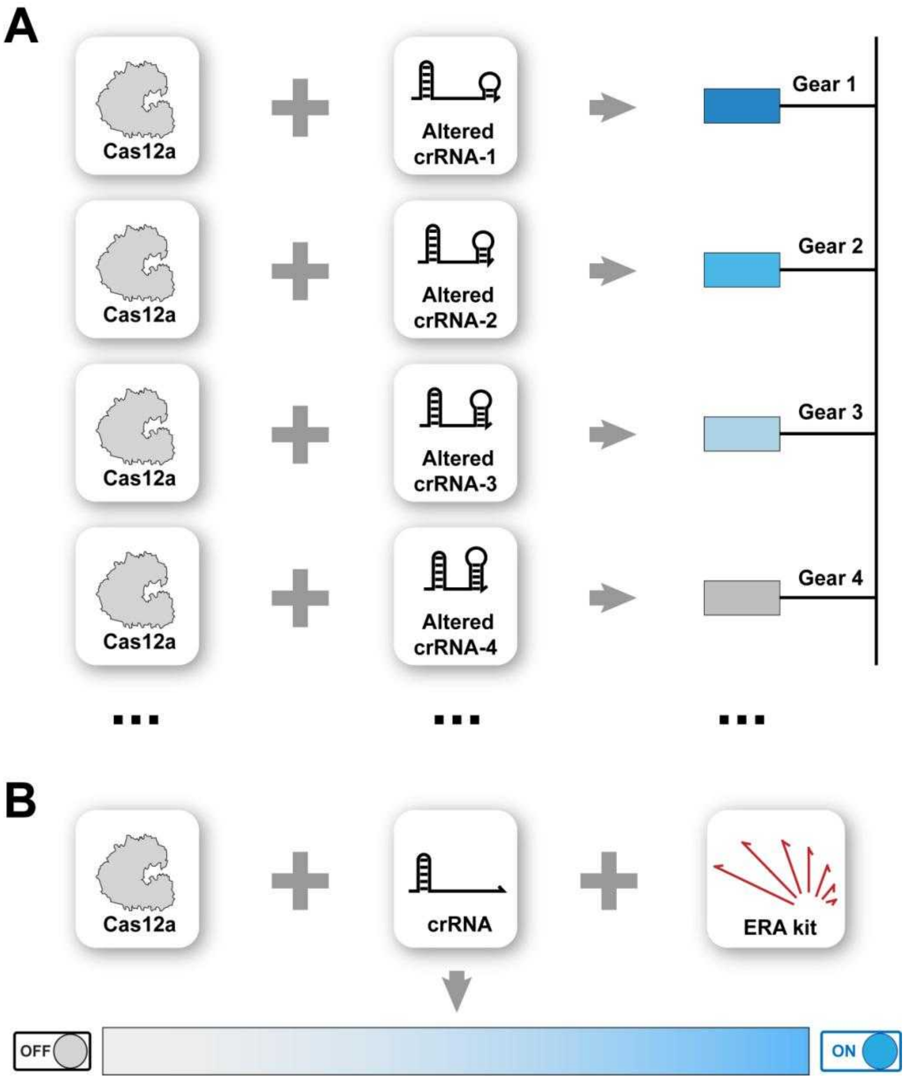

Scheme S1. (A) Introduction of different hairpin structures in crRNA can only regulate Cas12a activity in a graded manner. (B) ERR-controlled stepless regulation does not require changes to the original system.

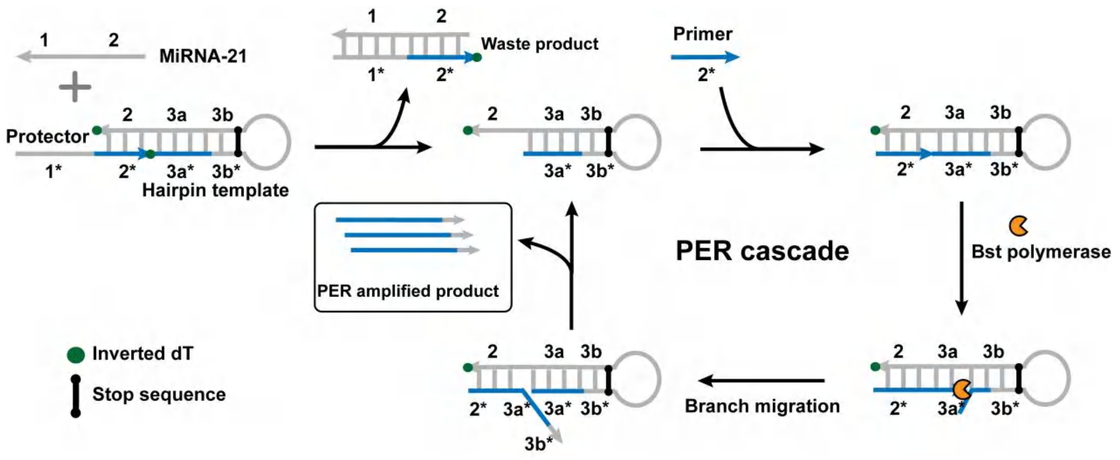

Scheme S2. Schematic diagram of the primer exchange reaction (PER). After the target (MiRNA-21) hybridizes with protector to form waste, the primer binding region on the hairpin template is exposed, and the primer binds to it and undergoes a PER cycle mediated by Bst polymerase to produce a large amount of amplification products. The blue part is the activation sequence of Cas12a, and the gray part is other sequences.

AAG
U U
UCUAC GUAGA

5'-UAAUU UUAAGUUCAAGGUGUGCGCAAUG-3'	crRNA
3'-GCGUUAC-5'	5' toehold-15nt
3'-ACACGCGUUAC-5'	5' toehold-11nt
3'-CACACGCGUUAC-5'	5' toehold-10nt
3'-CCACACGCGUUAC-5'	5' toehold-9nt
3'-UCCACACGCGUUAC-5'	5' toehold-8nt
3'-UUCCACACGCGUUAC-5'	5' toehold-7nt
3'-GUUCCACACGCGUUAC-5'	5' toehold-6nt
3'-AGUUCCACACGCGUUAC-5'	5' toehold-5nt
3'-AAGUUCCACACGCGUUAC-5'	5' toehold-4nt
3'-CAAGUUCCACACGCGUUAC-5'	5' toehold-3nt
3'-UCAAGUUCCACACGCGUUAC-5'	5' toehold-2nt
3'-UUCAAGUUCCACACGCGUUAC-5'	5' toehold-1nt
3'-AUUCAAGUUCCACACGCGUUAC-5'	5' toehold-0nt
3'-ATTCAAGTTCCACACGCGTTAC-5'	PM

AAG
U U
UCUAC GUAGA

5'-UAAUU UUAAGUUCAAGGUGUGCGCAAUG-3'	crRNA
3'-AUUCAAG-5'	3' toehold-15nt
3'-AUUCAAGUUCC-5'	3' toehold-11nt
3'-AUUCAAGUUCCA-5'	3' toehold-10nt
3'-AUUCAAGUUCCAC-5'	3' toehold-9nt
3'-AUUCAAGUUCCACA-5'	3' toehold-8nt
3'-AUUCAAGUUCCACAC-5'	3' toehold-7nt
3'-AUUCAAGUUCCACACG-5'	3' toehold-6nt
3'-AUUCAAGUUCCACACGC-5'	3' toehold-5nt
3'-AUUCAAGUUCCACACGCG-5'	3' toehold-4nt
3'-AUUCAAGUUCCACACGCGU-5'	3' toehold-3nt
3'-AUUCAAGUUCCACACGCGUU-5'	3' toehold-2nt
3'-AUUCAAGUUCCACACGCGUUA-5'	3' toehold-1nt
3'-AUUCAAGUUCCACACGCGUUAC-5'	3' toehold-0nt
3'-ATTCAAGTTCCACACGCGTTAC-5'	PM

Scheme S3. The sequence in Figure 1A-1D. Black is the crRNA sequence, red is the ERA sequence, and blue is the ssDNA-activator (PM).

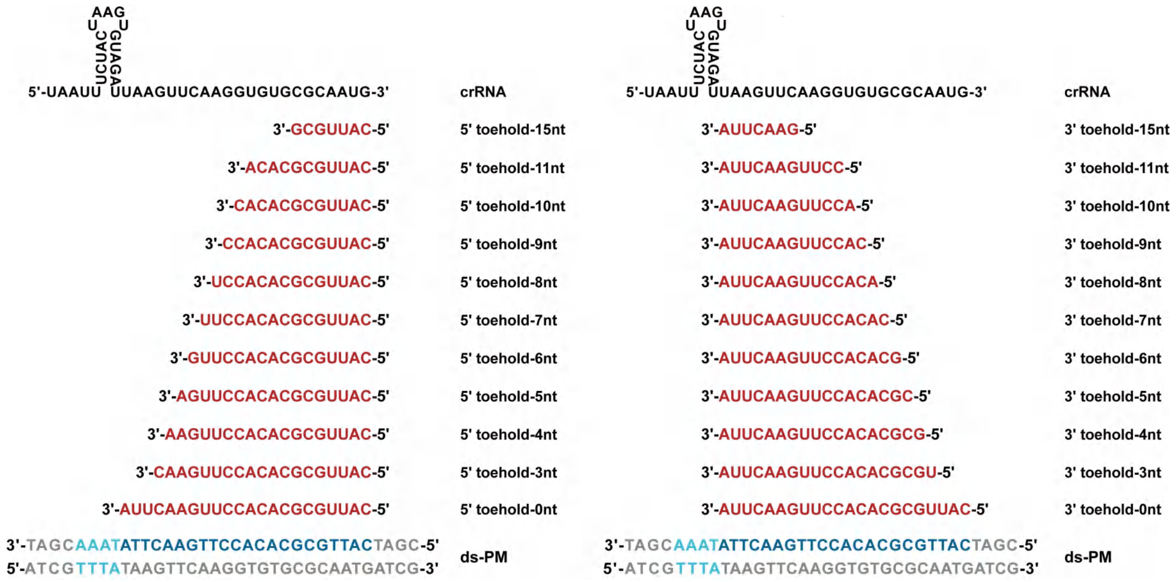

Scheme S4. The sequence in Figure S11-S12. black is the crRNA sequence, red is the ERA sequence. Blue part is ssDNA-activator, sky blue is the PAM sequence, and gray is the other sequences in the dsDNA-activator (PM).

AAG<br>U U<br>UCUAC GUAGA<br>5'-UAAUU UUGCGAGUAACGUCCACUAUCUC-3'	crRNA-β	AAG<br>U U<br>UCUAC GUAGA<br>5'-UAAUU UUGCGAGUAACGUCCACUAUCUC-3'	crRNA-β
3'-GAUAGAG-5'	5' toehold-15nt-β	3'-ACGCUCA-5'	3' toehold-15nt-β
3'-AGGUGAUAGAG-5'	5' toehold-11nt-β	3'-ACGCUCAUUGC-5'	3' toehold-11nt-β
3'-CAGGUGAUAGAG-5'	5' toehold-10nt-β	3'-ACGCUCAUUGCA-5'	3' toehold-10nt-β
3'-GCAGGUGAUAGAG-5'	5' toehold-9nt-β	3'-ACGCUCAUUGCAG-5'	3' toehold-9nt-β
3'-UGCAGGUGAUAGAG-5'	5' toehold-8nt-β	3'-ACGCUCAUUGCAGG-5'	3' toehold-8nt-β
3'-UUGCAGGUGAUAGAG-5'	5' toehold-7nt-β	3'-ACGCUCAUUGCAGGU-5'	3' toehold-7nt-β
3'-AUUGCAGGUGAUAGAG-5'	5' toehold-6nt-β	3'-ACGCUCAUUGCAGGUG-5'	3' toehold-6nt-β
3'-CAUUGCAGGUGAUAGAG-5'	5' toehold-5nt-β	3'-ACGCUCAUUGCAGGUGA-5'	3' toehold-5nt-β
3'-UCAUUGCAGGUGAUAGAG-5'	5' toehold-4nt-β	3'-ACGCUCAUUGCAGGUGAU-5'	3' toehold-4nt-β
3'-CUCAUUGCAGGUGAUAGAG-5'	5' toehold-3nt-β	3'-ACGCUCAUUGCAGGUGAUA-5'	3' toehold-3nt-β
3'-GCUCAUUGCAGGUGAUAGAG-5'	5' toehold-2nt-β	3'-ACGCUCAUUGCAGGUGAUAG-5'	3' toehold-2nt-β
3'-CGCUCAUUGCAGGUGAUAGAG-5'	5' toehold-1nt-β	3'-ACGCUCAUUGCAGGUGAUAGA-5'	3' toehold-1nt-β
3'-ACGCUCAUUGCAGGUGAUAGAG-5'	5' toehold-0nt-β	3'-ACGCUCAUUGCAGGUGAUAGAG-5'	3' toehold-0nt-β
3'-ACGCTCATTGCAGGTGATAGAG-5'	PM-β	3'-ACGCTCATTGCAGGTGATAGAG-5'	PM-β

Scheme S5. The sequence in Figure S13-S14. Black is the crRNA-β sequence, red is the ERA-β sequence, and blue is the ssDNA-activator (PM)-β.

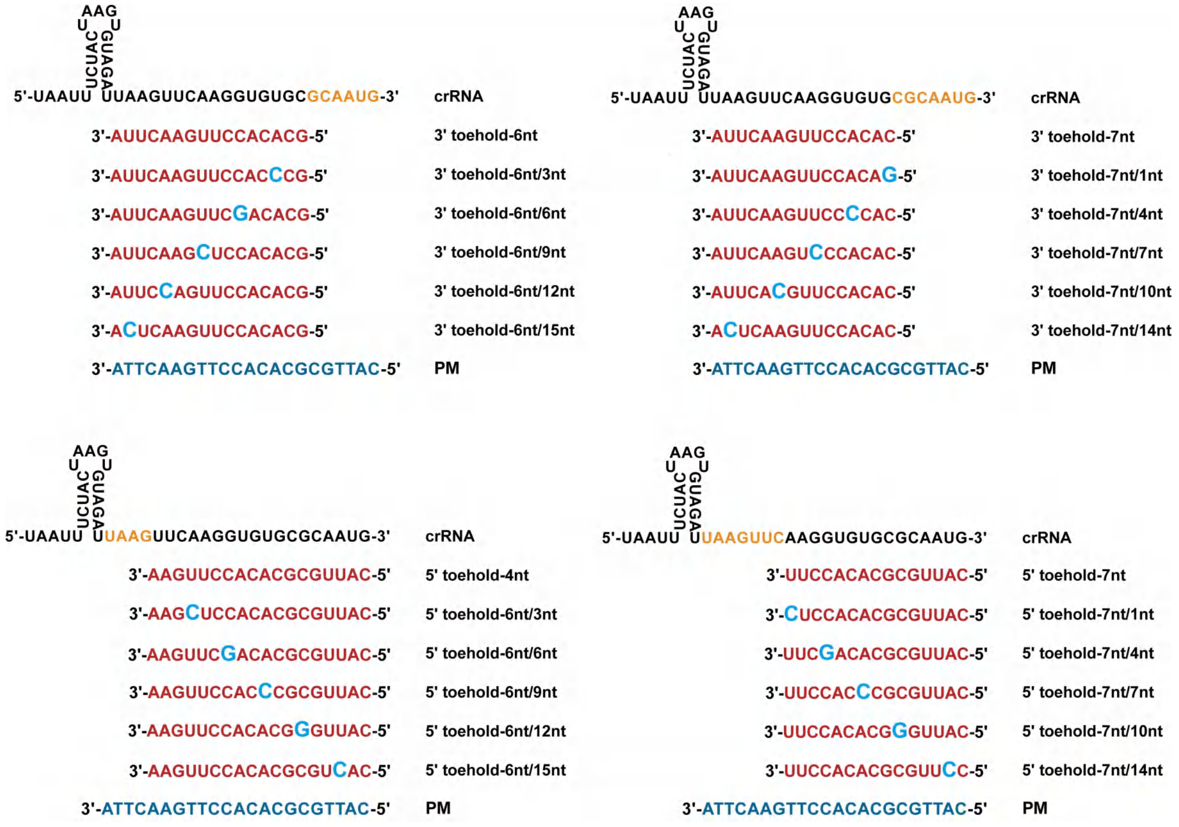

Scheme S6. The sequence in Figure 1E-1I. Black is the crRNA sequence, red is the ERA sequence, and bold blue is the mismatch base in ERA. Blue is the ssDNA-activator (PM).

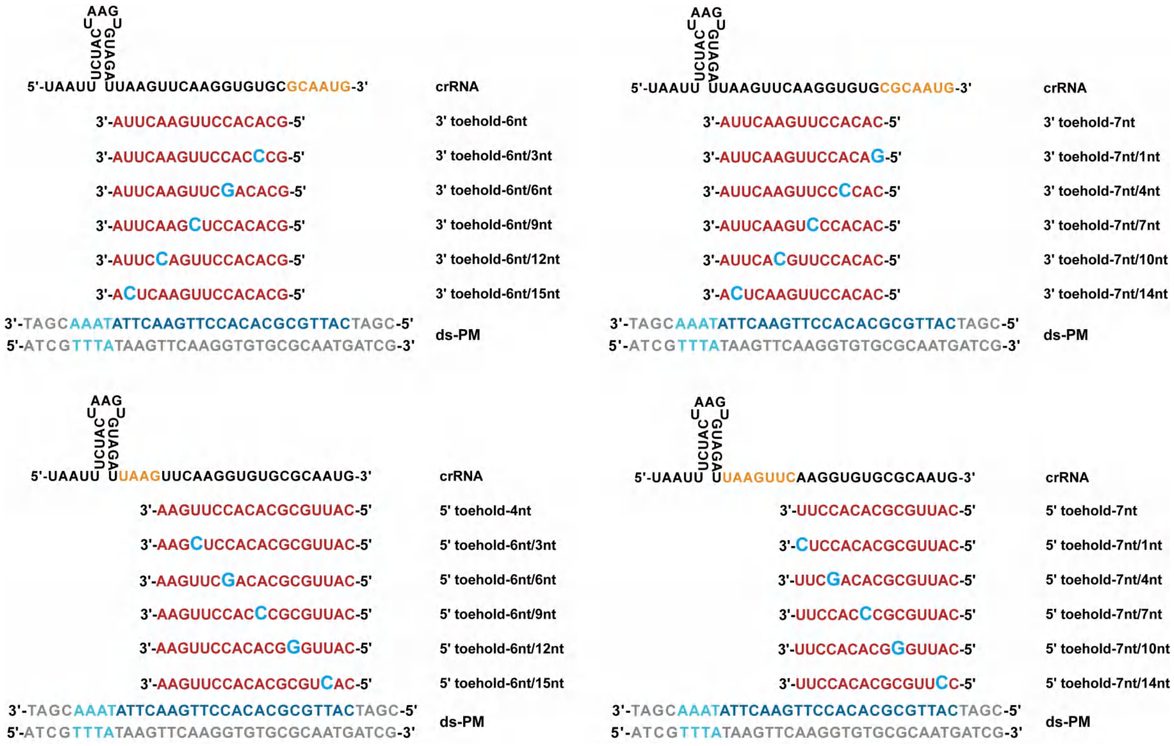

Scheme S7. The sequence in Figure S19-S22. Black is the crRNA sequence, red is the ERA sequence, and bold blue is the mismatch base in ERA. Blue part is ssDNA-activator, sky blue is the PAM sequence, and gray is the other sequences in the dsDNA-activator (PM).

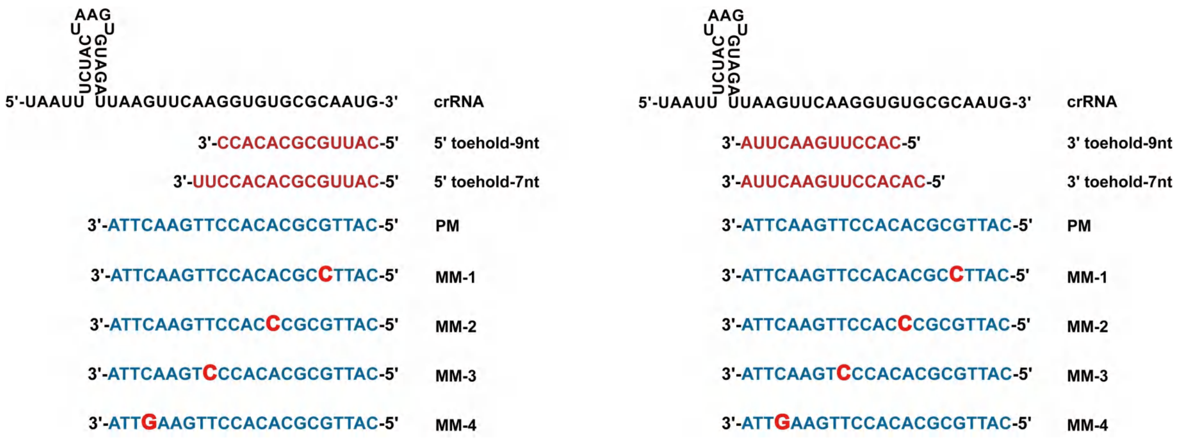

Scheme S8. The sequence in Figure 2C-2E and Figure S8. The sequence in Figure 1E-1I. Black is the crRNA sequence, red is the ERA sequence, blue is thessDNA-activator (PM or MM), and bold red is the mismatch base in activator.

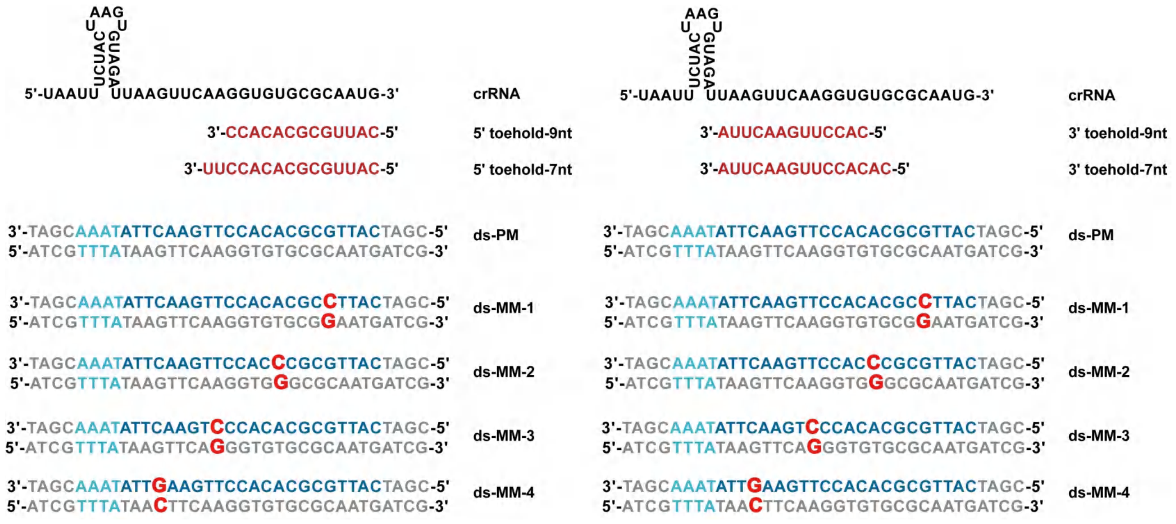

Scheme S9. The sequence in Figure S36. Black is the crRNA sequence, red is the ERA sequence. Blue part is ssDNA-activator, and bold blue is the mismatch base in dsDNA-activator (PM or MM). Sky blue is the PAM sequence, and gray is other sequences in the activator.

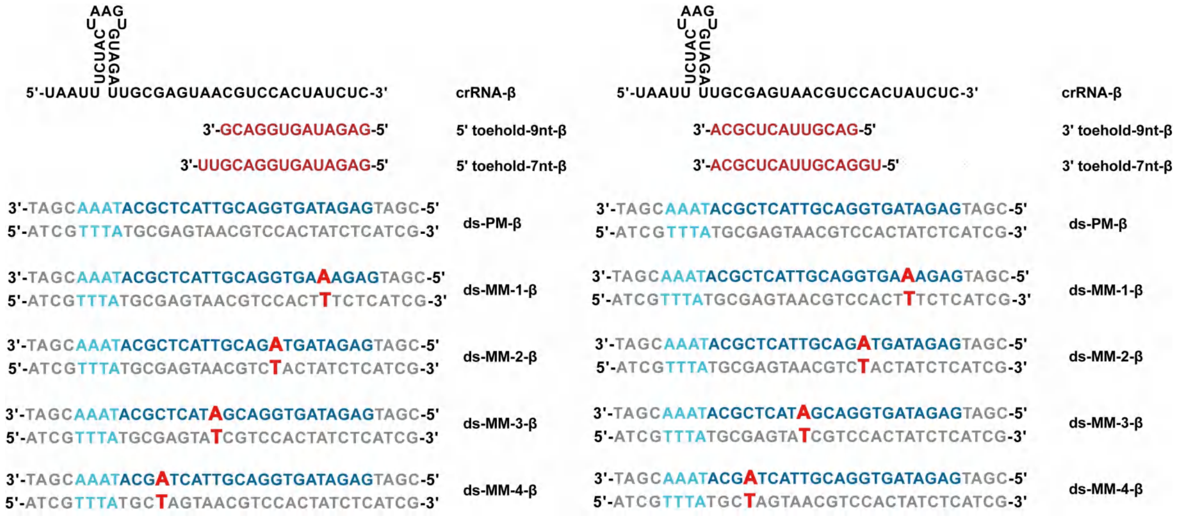

Scheme S10. The sequence in Figure S40. Black is the crRNA-β sequence, red is the ERA-β sequence. The blue part is ssDNA-activator-β and bold blue is the mismatch base in dsDNA-activator (PM or MM). Sky blue is the PAM sequence, and gray is other sequences in the activator.

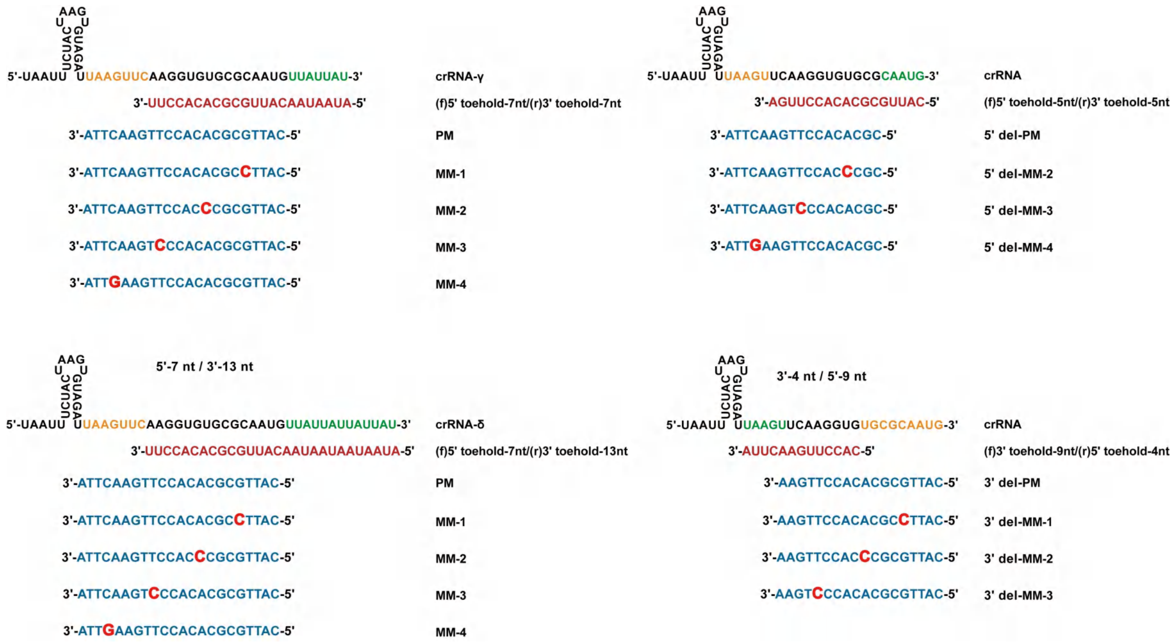

Scheme S11. The sequence in Figure 3. Black is the crRNA sequence, the orange and green parts are the forward toehold (f) and backward toheold (r) sequences in the TE reaction system. Red is the corresponding ERA sequence. Blue is the ssDNA-activator, the red bold letters are the mismatch bases in activator.

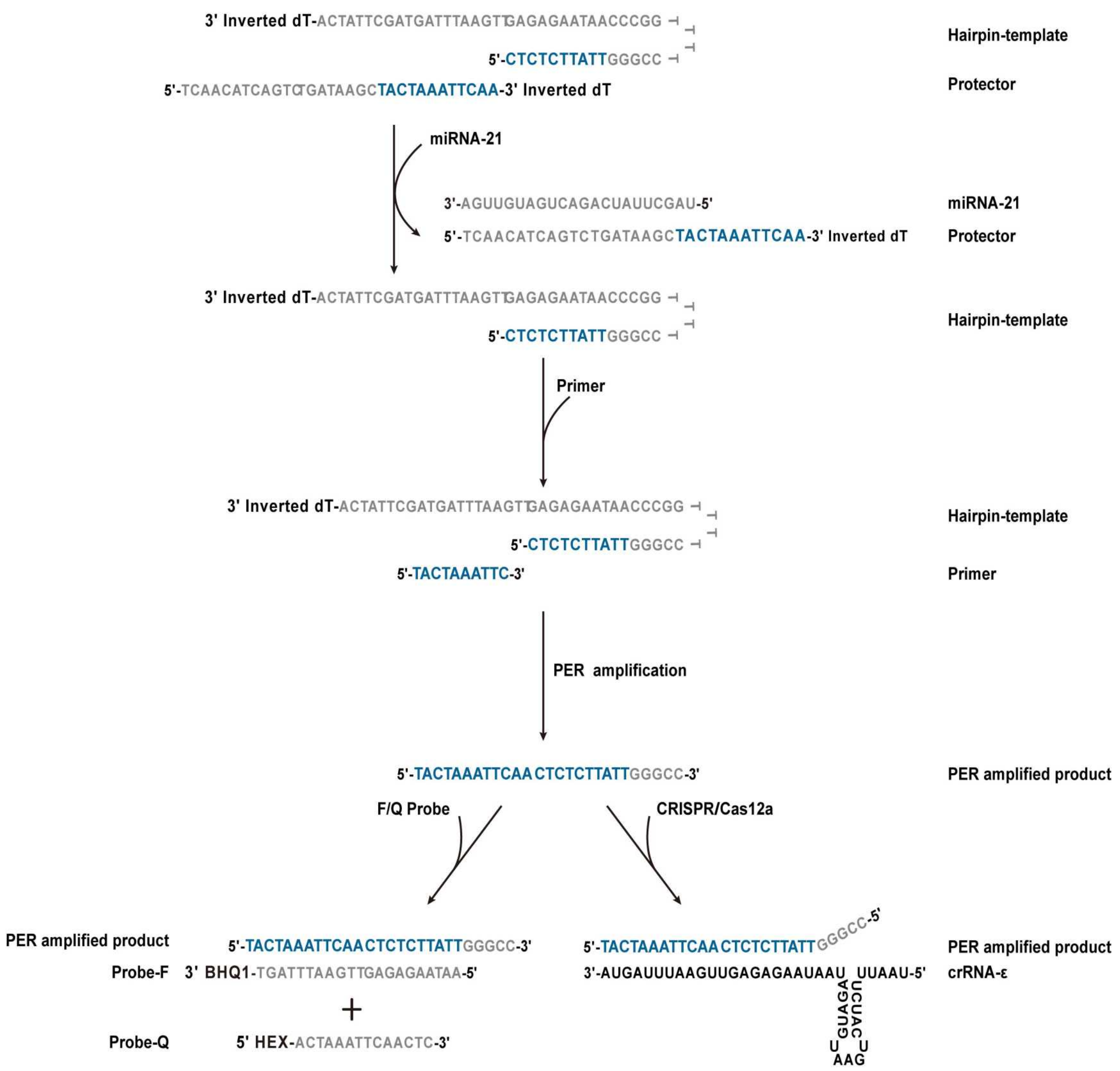

Scheme S12. The sequence in Scheme S3. Blue is the ssDNA-activator part of Cas12a, black is the crRNA-ε sequence, and gray is other sequences.

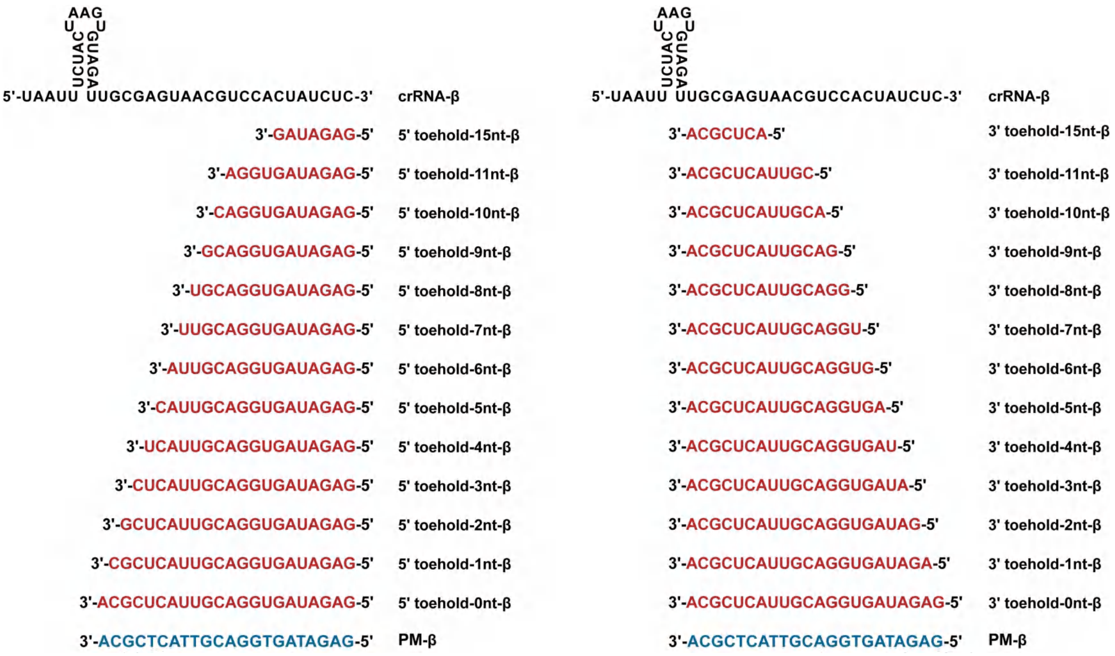

Scheme S13. The sequence in Figure 2C-2E and Figure S8. The crRNA-β sequence is in black and the ERA-β sequence is in red. Blue is the ssDNA-β activator.

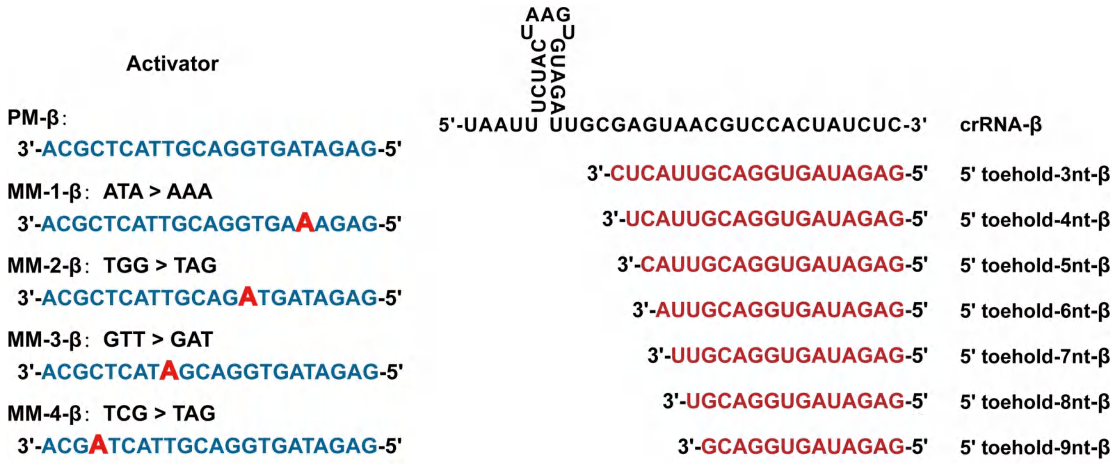

Scheme S14. The sequence in Figure S28-S29. Black is the crRNA-β sequence, red is the ERA-β sequence. Blue is the ssDNA-activator-β (PM or MM), and bold red is the mismatch base in activator.

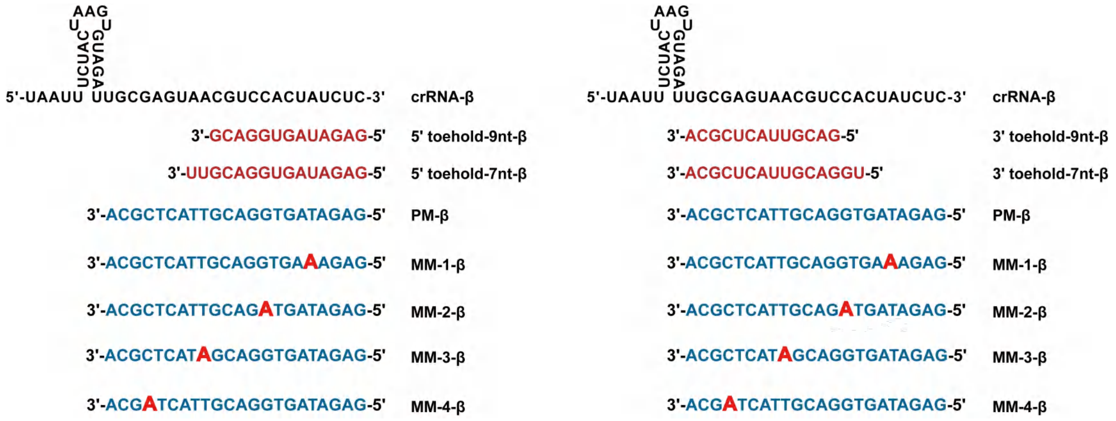

Scheme S15. The sequence in Figure S30-S32. Black is the crRNA-β sequence, red is the ERA-β sequence. Blue is the ssDNA-activator-β (PM or MM), and bold red is the base in activator.

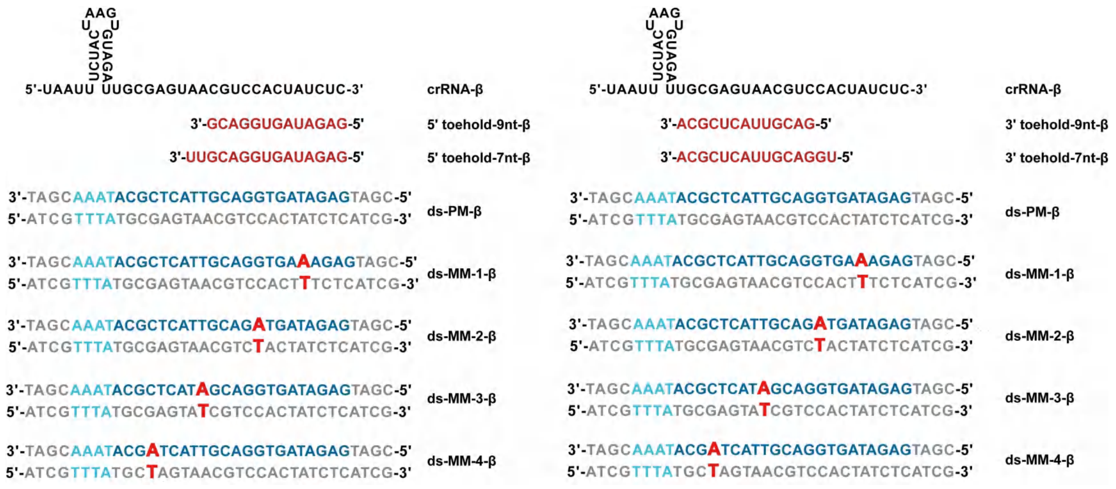

Scheme S16. The sequence in Figure S38-S41. Black is the crRNA-β sequence, red is the ERA-β sequence. The blue part is ssDNA-activator-β and bold red is the mismatch base in dsDNA-activator (PM or MM). Sky blue is the PAM sequence, and gray is other sequences in the activator.

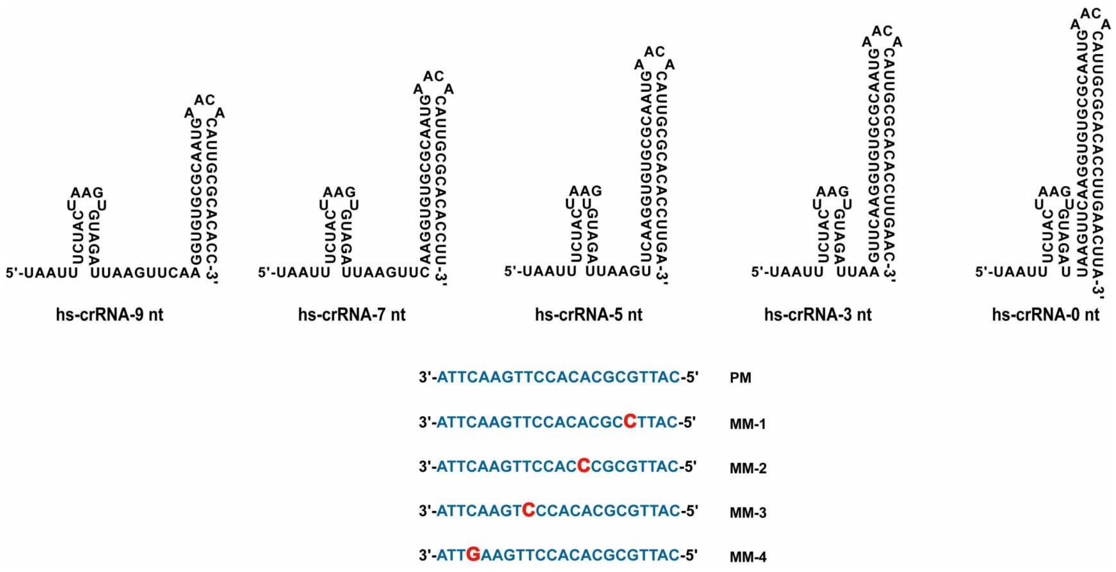

Scheme S17. The sequence in Scheme S1 and Figure S42-43. Black is the hs-crRNA sequence, blue is ssDNA-activator and bold red is the mismatch base in activator (PM or MM) .

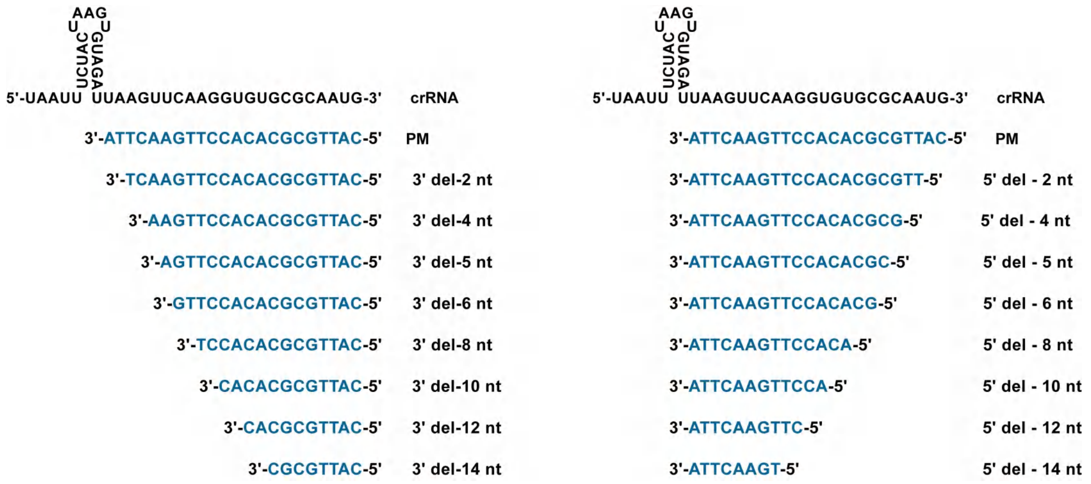

Scheme S18. The sequence in Figure S44-S45. Black is the crRNA-β sequence. Blue part is the ssDNA-activator, one end of which is deleted with bases of different lengths.

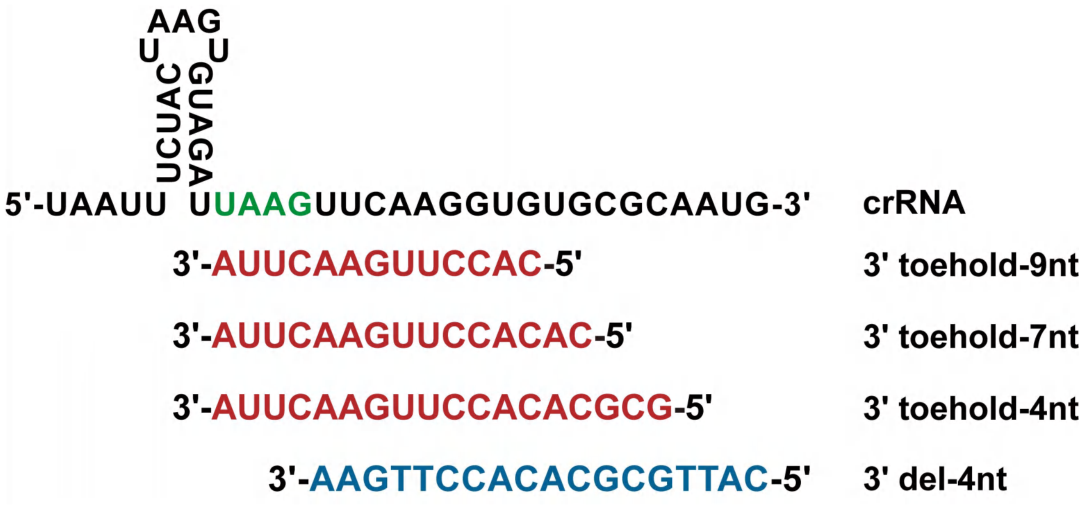

Scheme S19. The sequence in Figure S46. Black is the crRNA sequence, and green part is the reverse (r) toehold. Red is corresponding ERA sequence, blue part is ssDNA-activator.

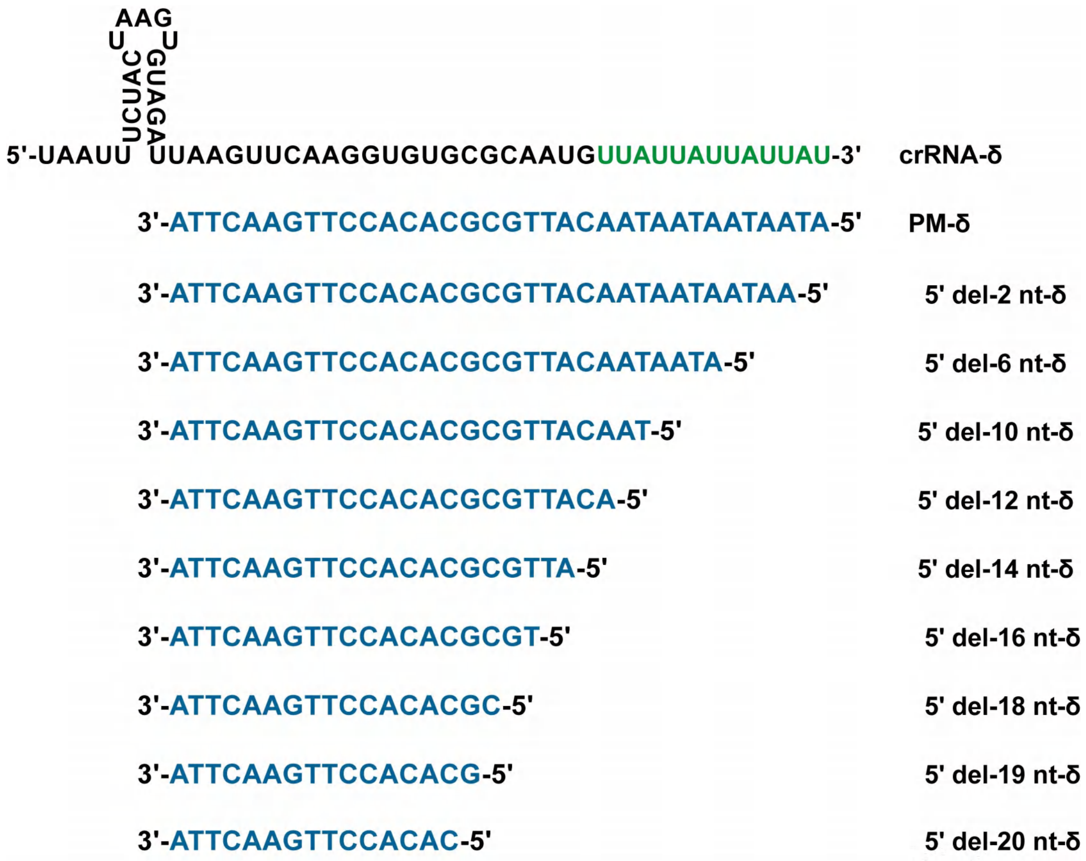

Scheme S20. The sequence in Figure S47. Black is the crRNA-δ sequence, green part is the extension of crRNA. Blue is the ssDNA-activator (PM-δ) with different length bases deleted at its 5' ends.

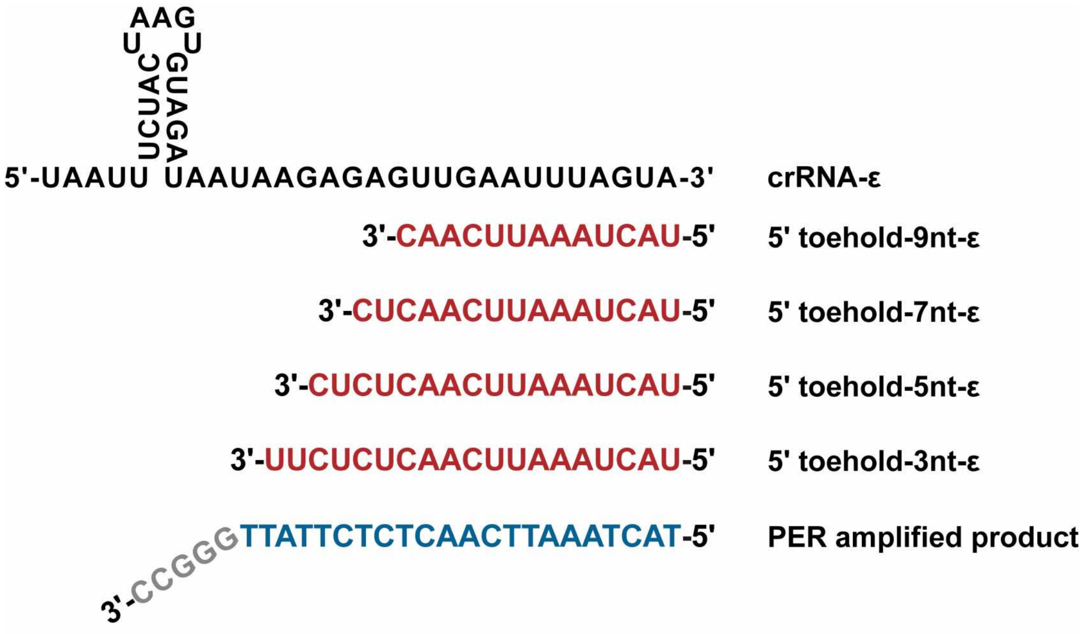

Scheme S21. The sequence in Figure S52. Black is the crRNA-ε sequence, red is the ERA-ε sequence. Blue part is ssDNA-activator and gray part is other sequences in PER aplification product.

Supplementary Figures

Multidimensional control of activation velocity and cleavage activity

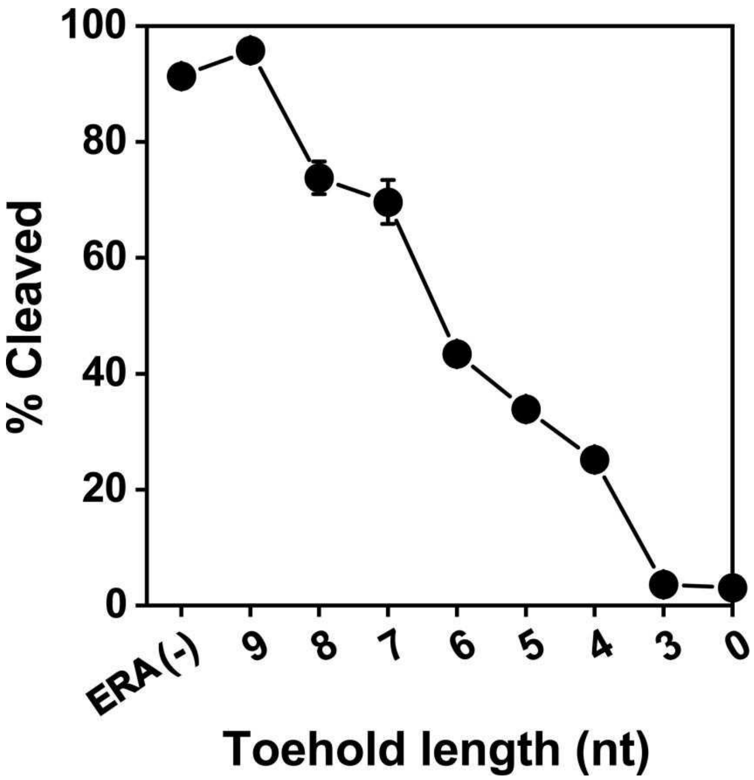

Figure S1. Fluorescence cleavage rate at 20 min of ssDNA-activated ERA-Cas12a with different length of 5' toehold. ERA (-) means no ERA is added. Error bars represented the standard deviation calculated from three independent experiments.

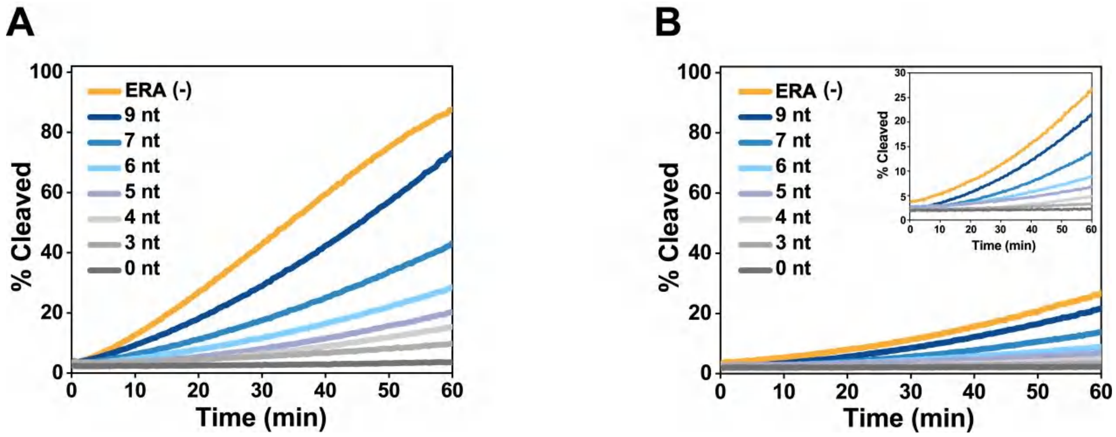

Figure S2. Fluorescence cleavage rate at 20 min of ssDNA-activated ERA-Cas12a with different length of 5' toehold at low target concentrations of 1N (A) or 100 pM (B). ERA (-) means no ERA is added.

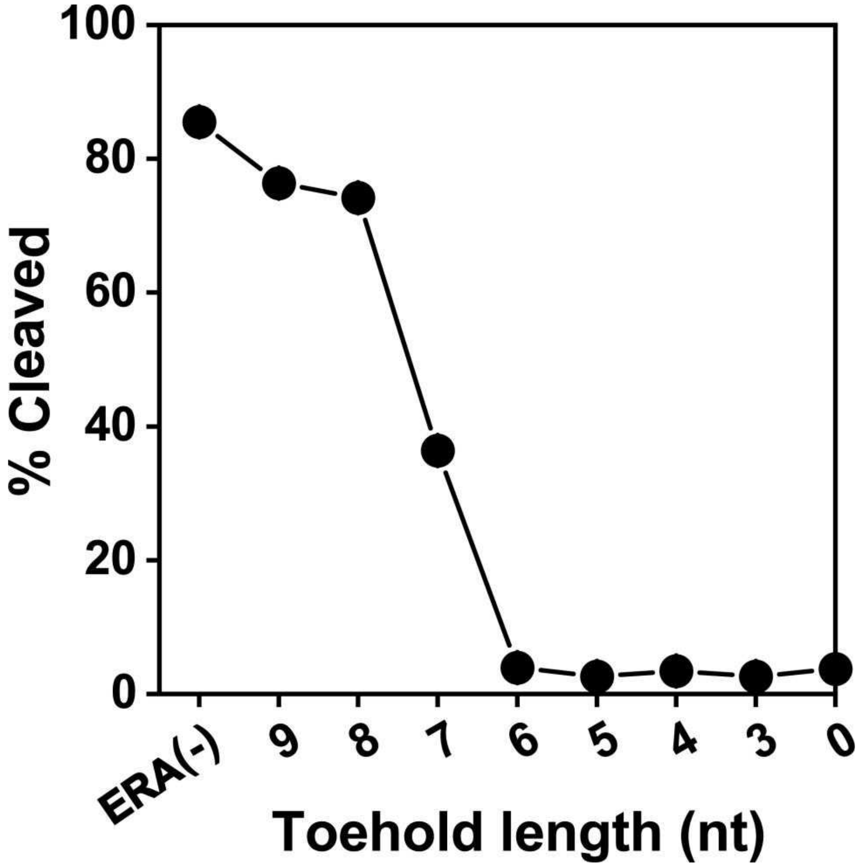

Figure S3. Fluorescence cleavage rate at 20 min of ssDNA-activated ERA-Cas12a with different length of 3' toehold. ERA (-) means no ERA is added. Error bars represented the standard deviation calculated from three independent experiments.

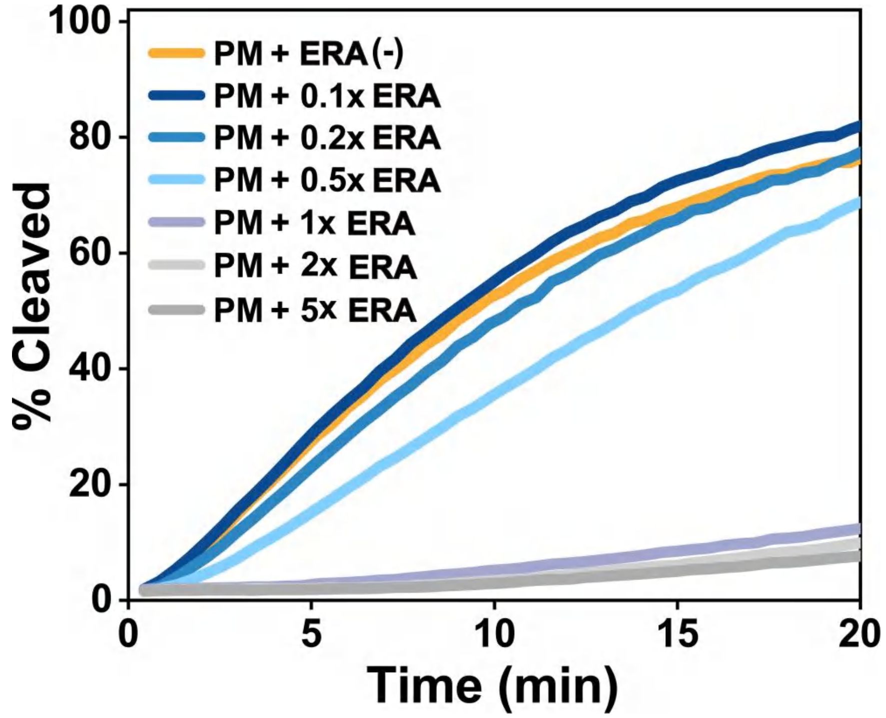

Figure S4. Cleavage efficiency of ssDNA-PM-activated Cas12a with different addition ratio of 5' toehold-4 nt ERA. ERA (-) means no ERA is added.

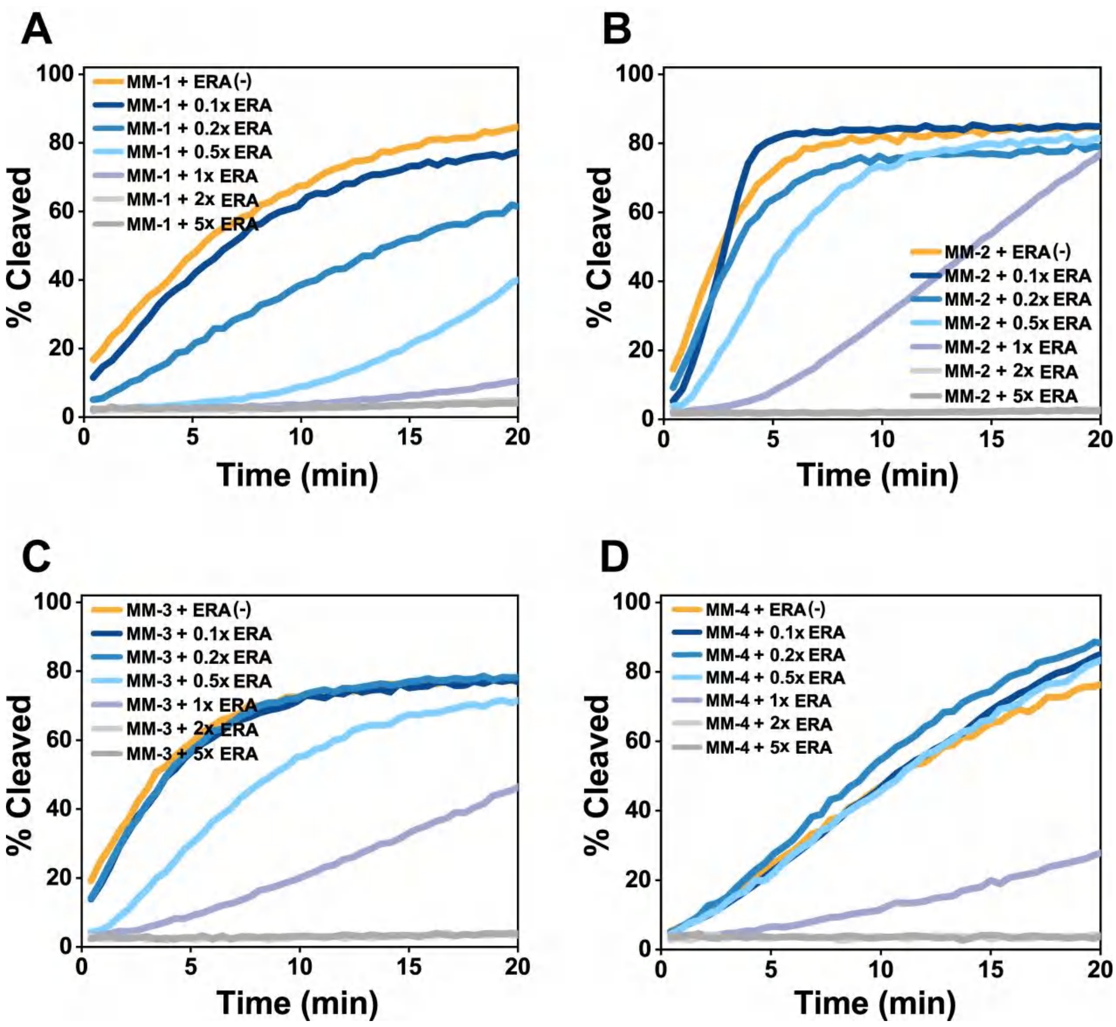

Figure S5. Cleavage efficiency of Cas12a activated by ssDNA-MM-1 (A), MM-2 (B), MM-3 (C), and MM-4 (D) at different addition ratios of 5' toehold-4nt ERA. ERA (-) means no ERA is added.

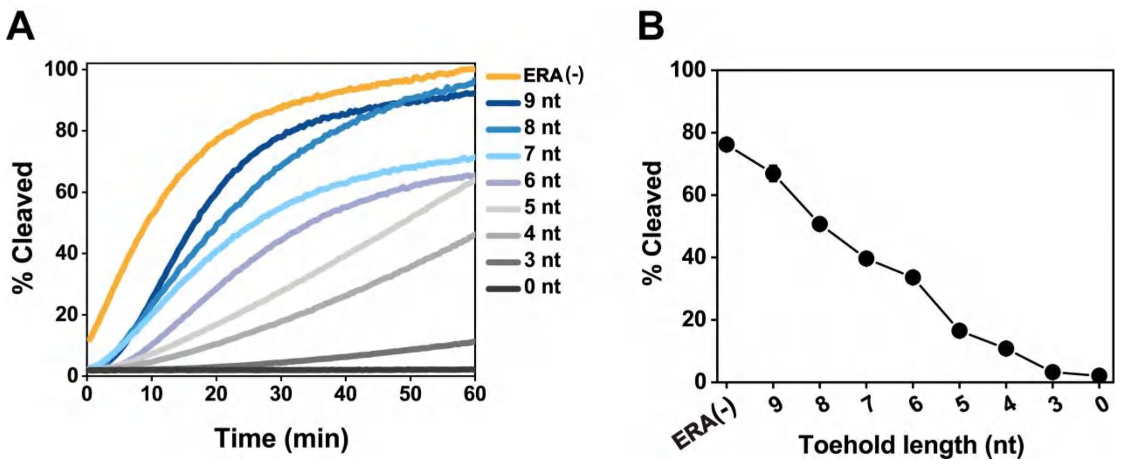

Figure S6. Real-time fluorescence curve (A) and fluorescence cleavage rate at 20 min (B) of ssDNA-activated ERA-β-Cas12a with different length of 5' toehold. ERA (-) means no ERA is added. Error bars represented the standard deviation calculated from three independent experiments.

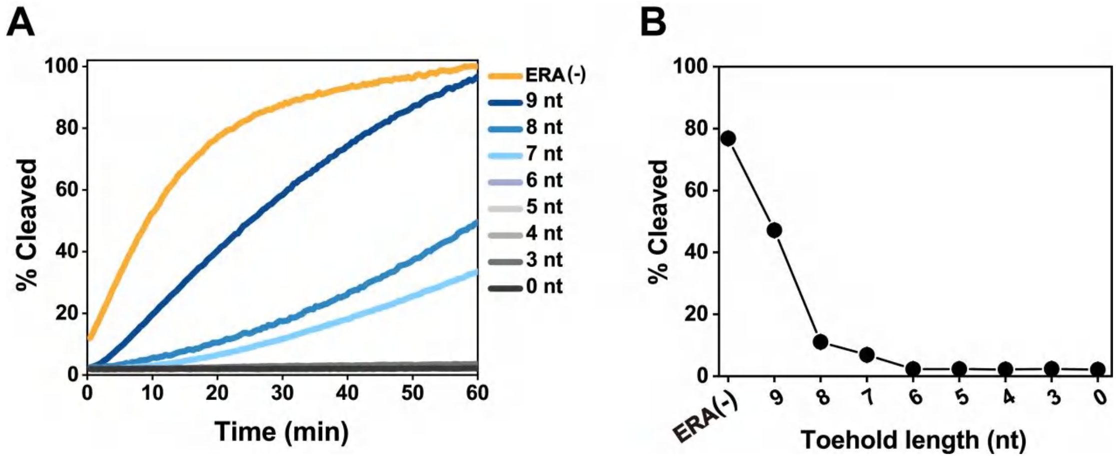

Figure S7. Real-time fluorescence curve (A) and fluorescence cleavage rate at 20 min (B) of ssDNA-activated ERA-Cas12a-β with different length of 3' toehold. ERA (-) means no ERA is added. Error bars represented the standard deviation calculated from three independent experiments.

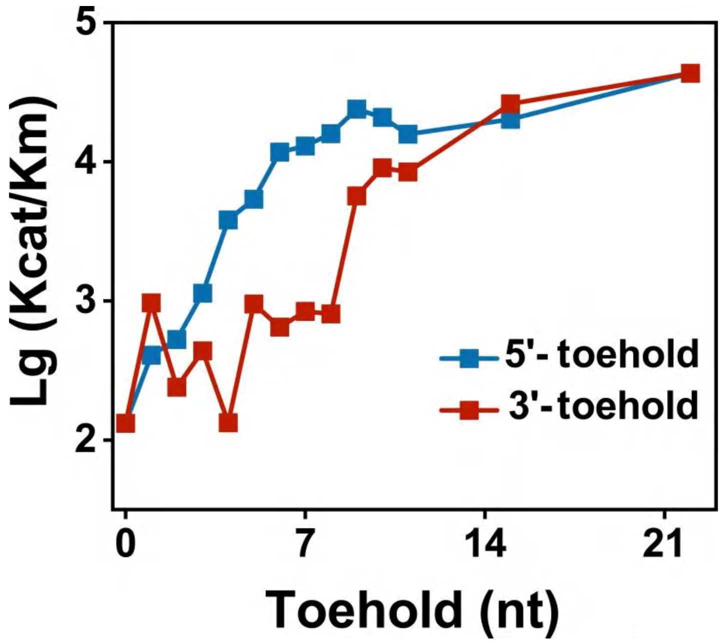

Figure S8. Relationship between toehold length and direction on the ssDNA-activated ERA-β-Cas12a catalytic efficiency (k_{cat}/K_M). Error bars represented the standard deviation calculated from three independent experiments.

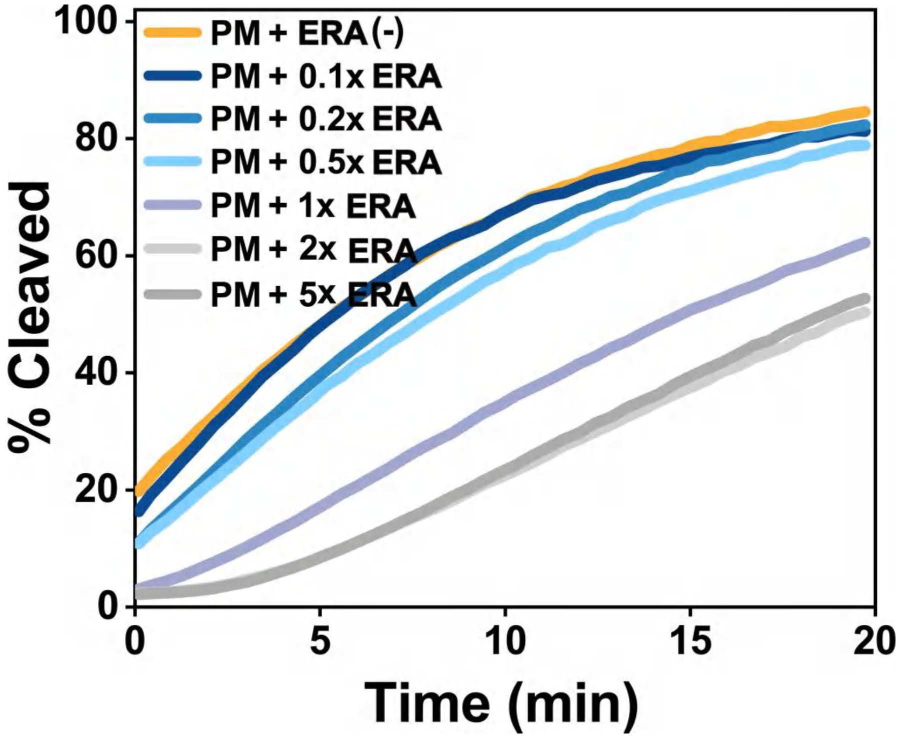

Figure S9. Cleavage efficiency of ssDNA-PM activated Cas12a with different addition ratio of 5' toehold-7 nt ERA-β. ERA (-) means no ERA is added.

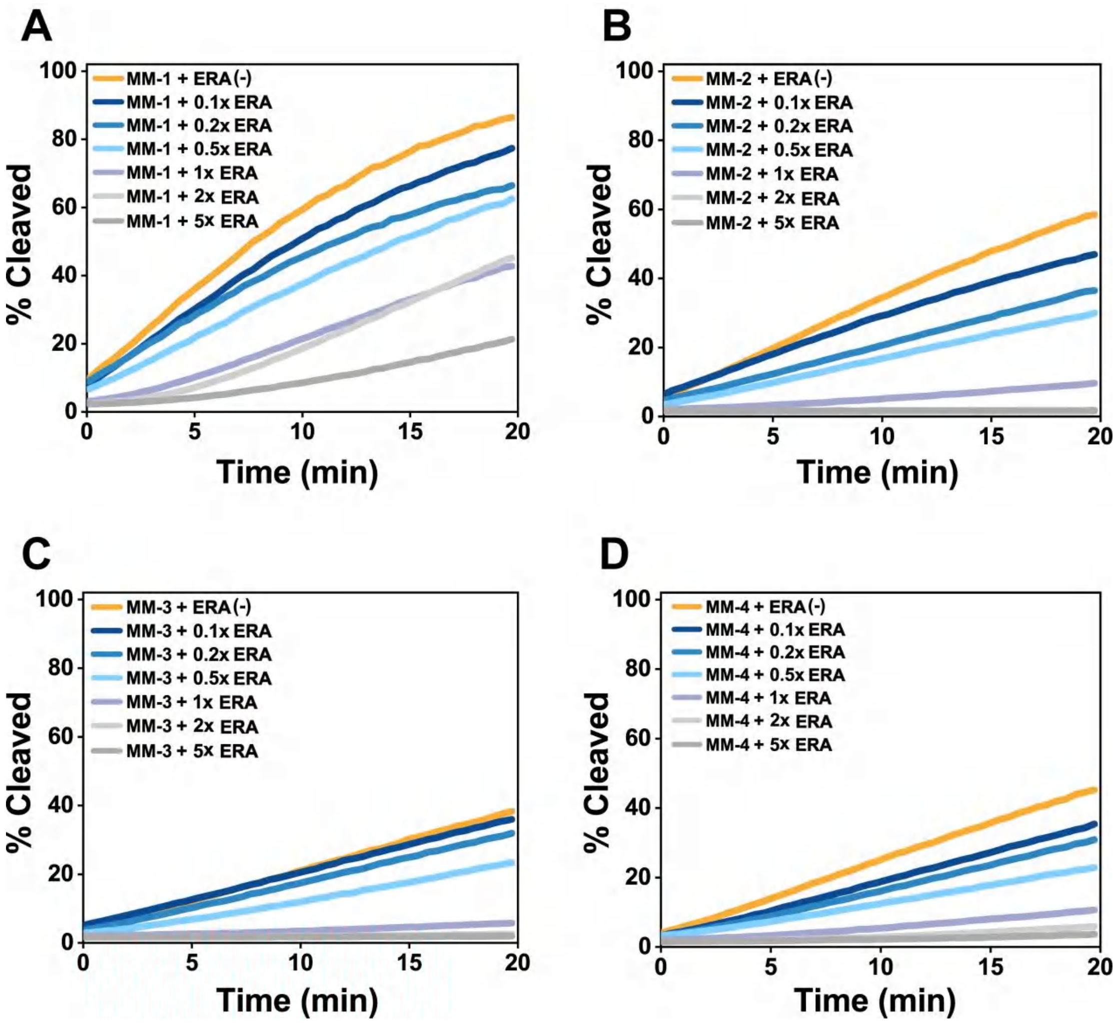

Figure S10. Cleavage efficiency of Cas12a activated by ssDNA-MM-1 (A), MM-2 (B), MM-3 (C) and MM-4 (D) at different addition ratios of 5' toehold-7 nt ERA-β. ERA (-) means no ERA is added.

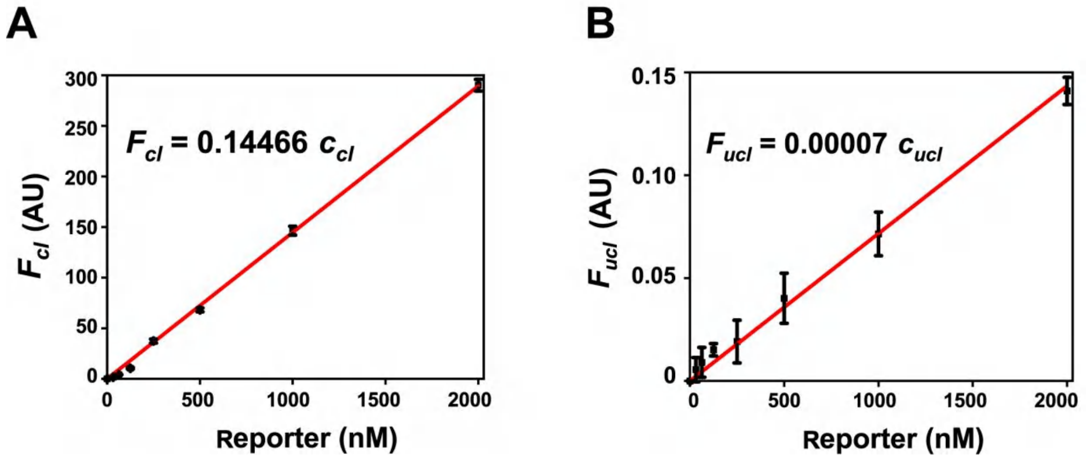

Figure S11. (A) Background-subtracted fluorescence F_{cl} versus concentration of cleaved reporter c_{cl}. (B) Background-subtracted fluorescence F_{ucl} versus concentration of uncleaved reporters c_{ucl}. Error bars represented the standard deviation calculated from three independent experiments.

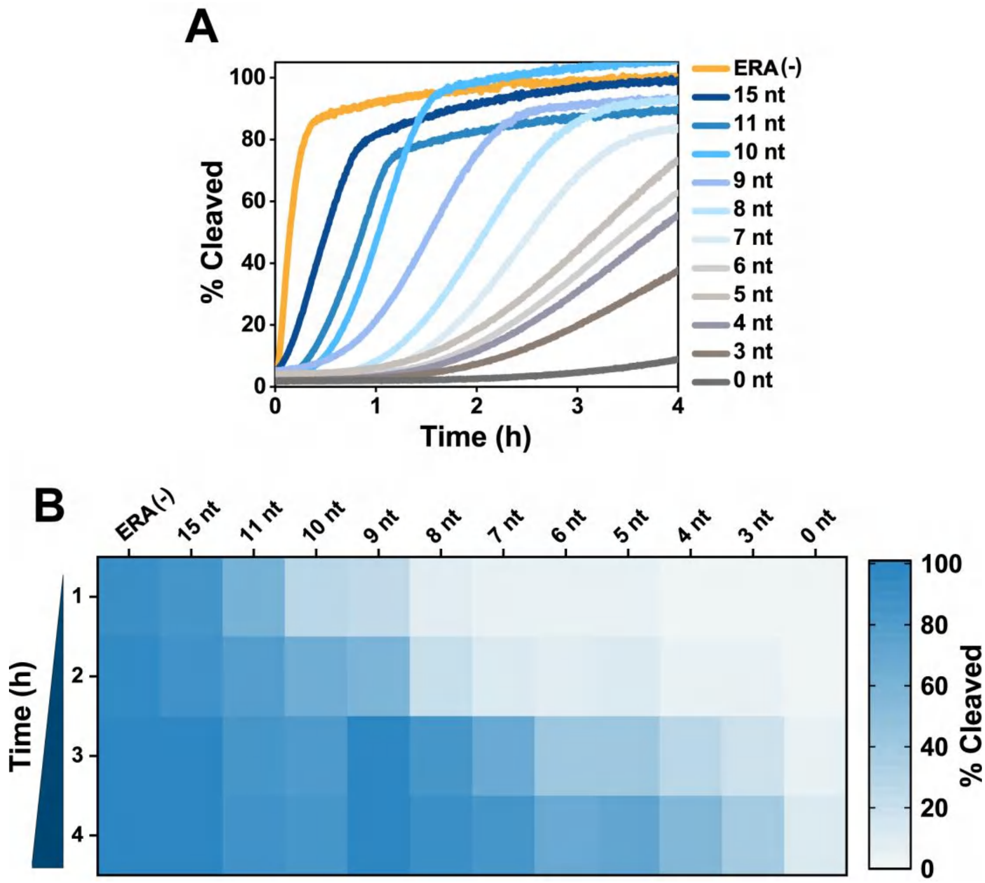

Figure S12. Real-time fluorescence curves (A) and %Cleaved-time heat map (B) of ERA-Cas12a with different lengths of 5' toehold activated by dsDNA. ERA (-) means no ERA is added.

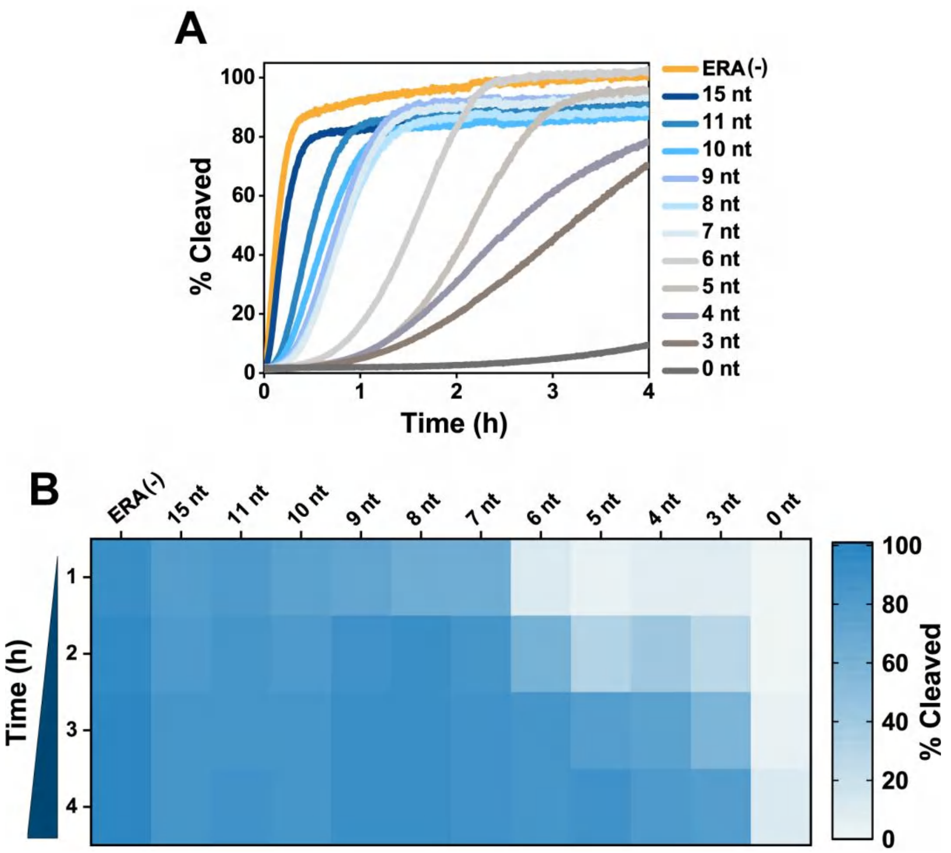

Figure S13. Real-time fluorescence curves (A) and %Cleaved-time heat map (B) of ERA-Cas12a with different lengths of 3' toehold activated by dsDNA. ERA (-) means no ERA is added.

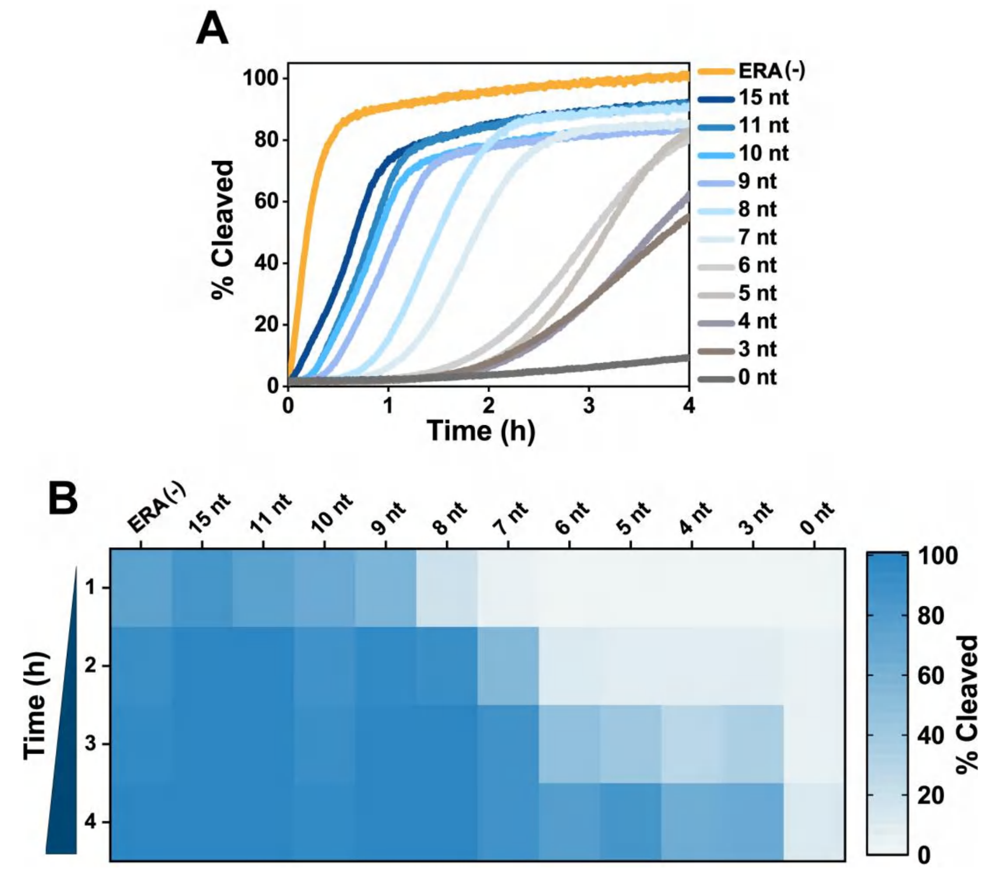

Figure S14. Real-time fluorescence curves (A) and %Cleaved-time heat map (B) of ERA-Cas12a with different lengths of 5' toehold-β activated by dsDNA. ERA (-) means no ERA is added.

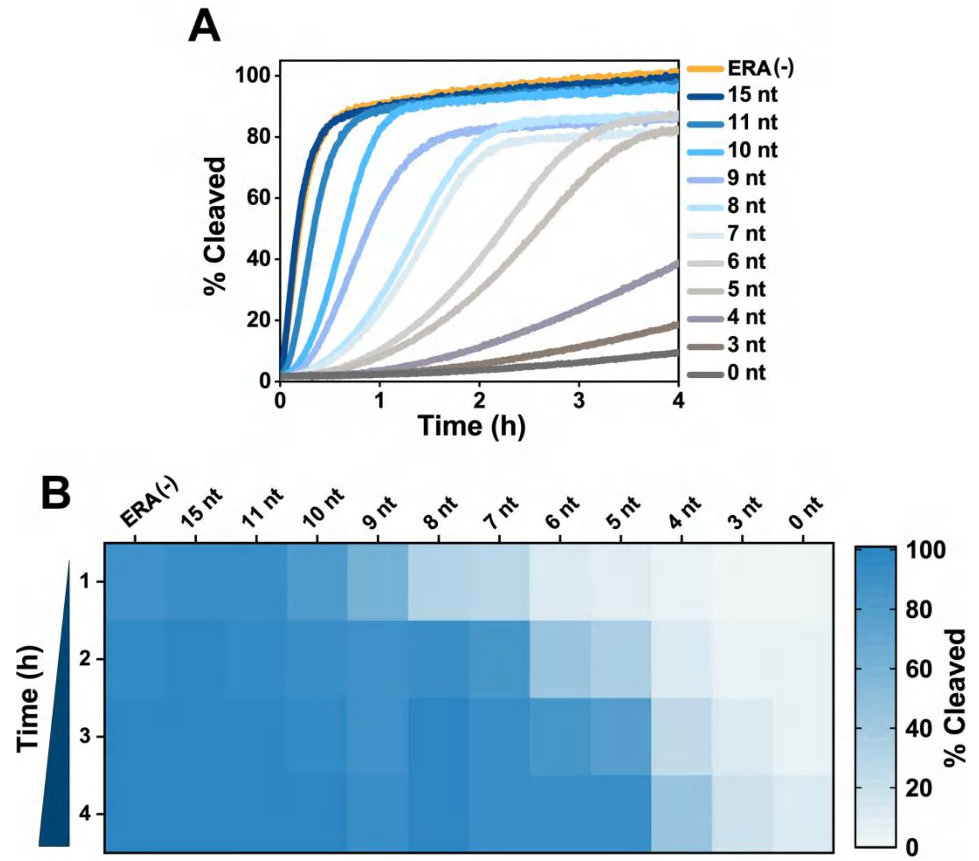

Figure S15. Real-time fluorescence curves (A) and %Cleaved-time heat map (B) of ERA-Cas12a with different lengths of 3' toehold-β activated by dsDNA. ERA (-) means no ERA is added.

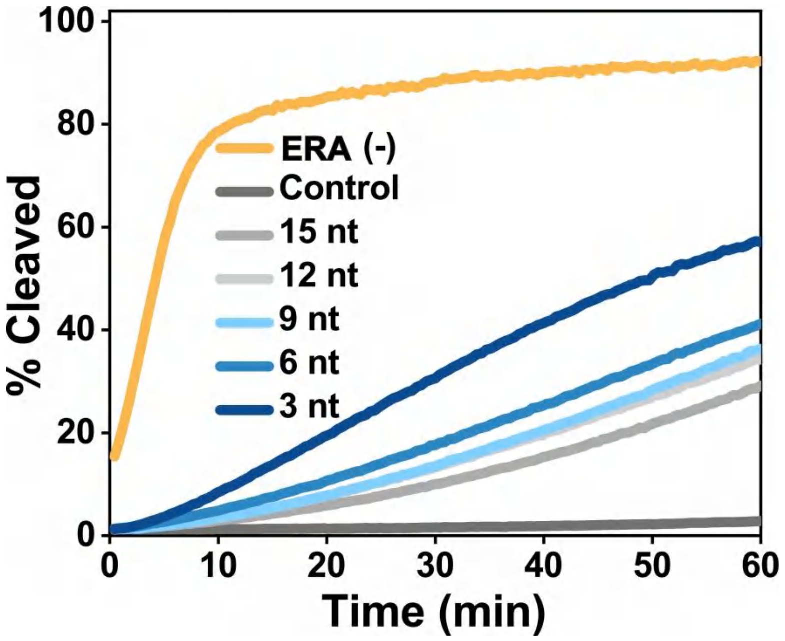

Figure S16. Cleavage rate kinetics of ssDNA-activated ERA-Cas12a with 3' toehold-6 nt (different ERA mismatched sites). ERA (-) means no ERA is added.

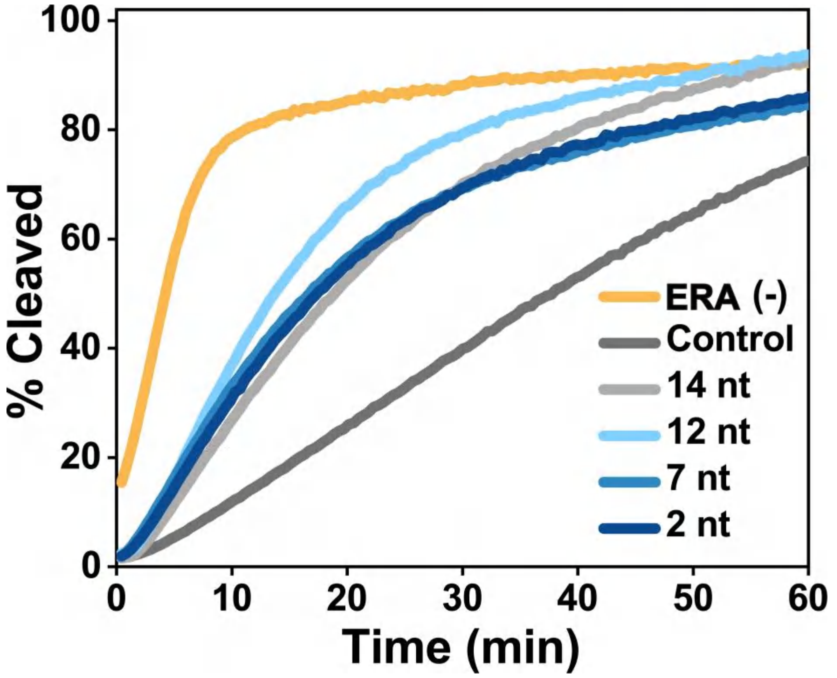

Figure S17. Cleavage rate kinetics of ssDNA-activated ERA-Cas12a with 3' toehold-7 nt (different ERA mismatched sites). ERA (-) means no ERA is added.

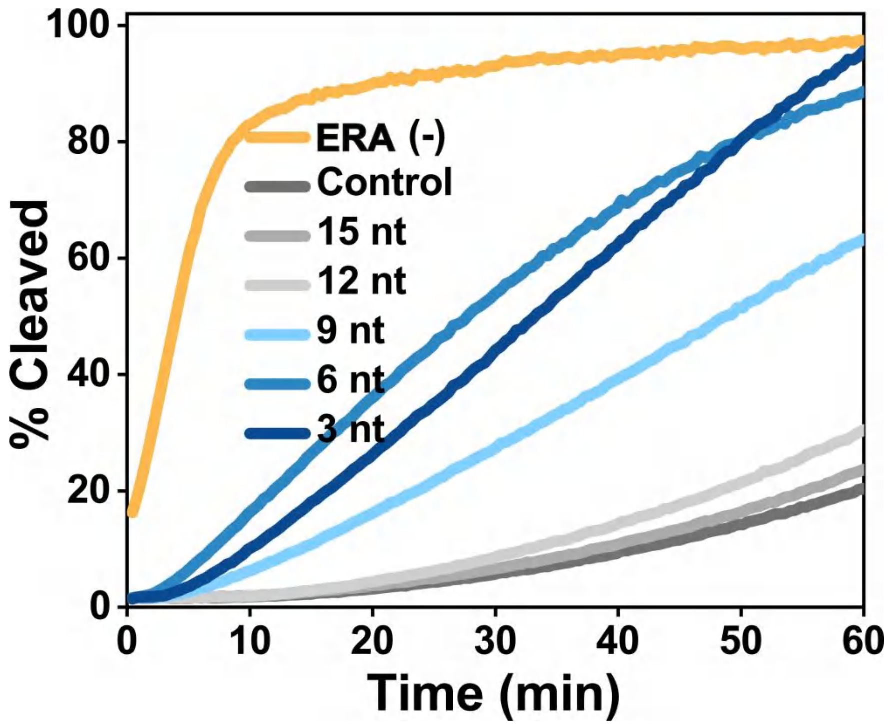

Figure S18. Cleavage rate kinetics of ssDNA-activated ERA-Cas12a with 5' toehold-4 nt (different ERA mismatched sites). ERA (-) means no ERA is added.

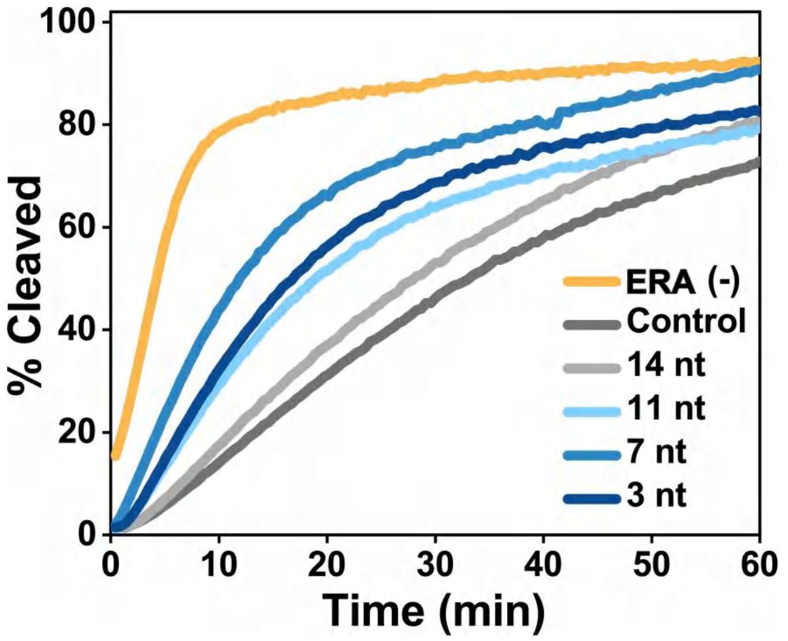

Figure S19. Cleavage rate kinetics of ssDNA-activated ERA-Cas12a with 3' toehold-7 nt (different ERA mismatched sites). ERA (-) means no ERA is added.

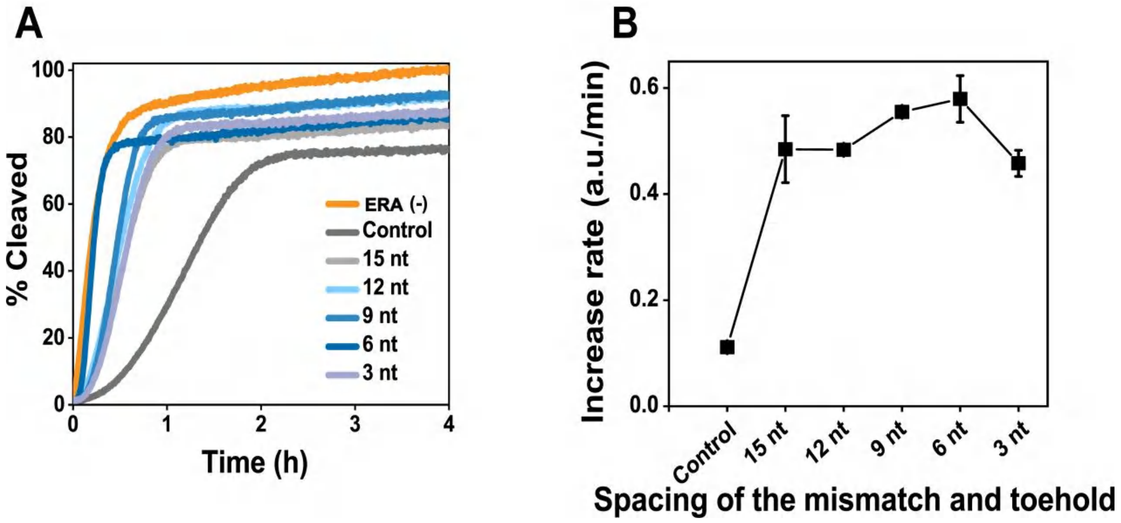

Figure S20. Cleavage rate kinetics (A) and fluorescence increase rate (B) of dsDNA-activated ERA-Cas12a with 3' toehold-6 nt (different ERA mismatched sites). ERA (-) means no ERA is added. Error bars represented the standard deviation calculated from three independent experiments.

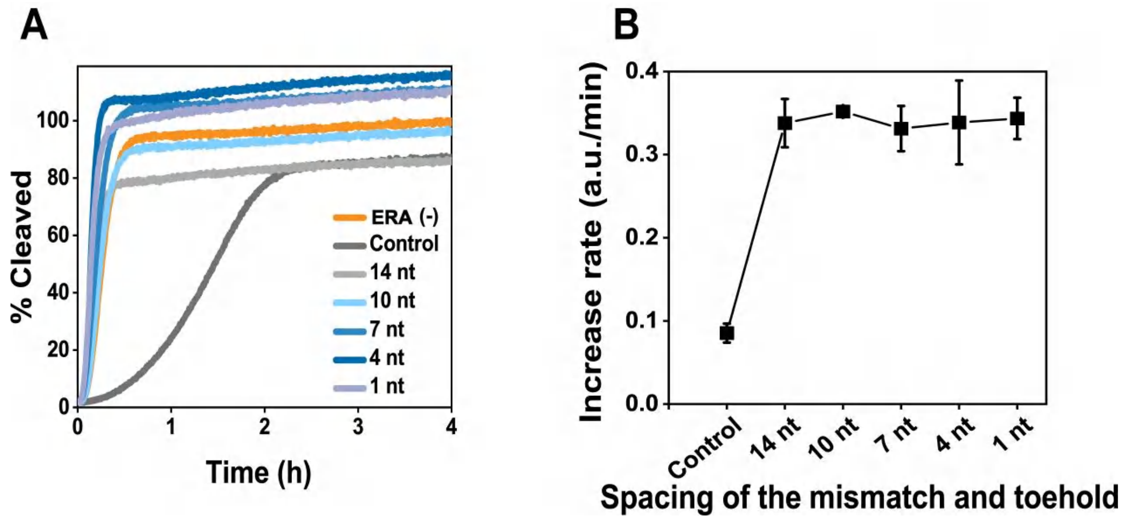

Figure S21. Cleavage rate kinetics (A) and fluorescence increase rate (B) of dsDNA-activated ERA-Cas12a with 3' toehold-7 nt (different ERA mismatched sites). ERA (-) means no ERA is added. Error bars represented the standard deviation calculated from three independent experiments.

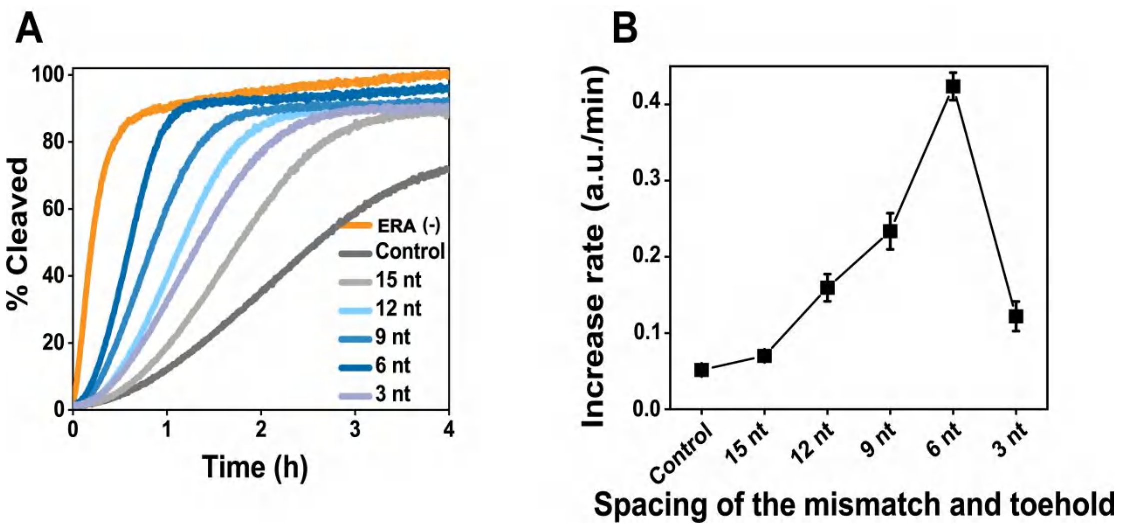

Figure S22. Cleavage rate kinetics (A) and fluorescence increase rate (B) of dsDNA-activated ERA-Cas12a with 5' toehold-4 nt (different ERA mismatched sites). ERA (-) means no ERA is added. Error bars represented the standard deviation calculated from three independent experiments.

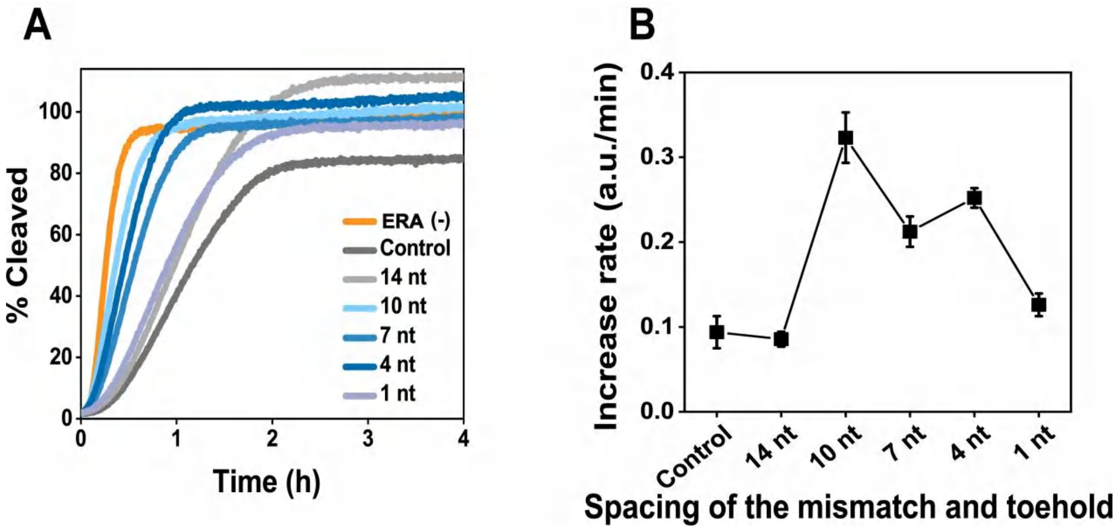

Figure S23. Cleavage rate kinetics (A) and fluorescence increase rate (B) of dsDNA-activated ERA-Cas12a with 5' toehold-7 nt (different ERA mismatched sites). ERA (-) means no ERA is added. Error bars represented the standard deviation calculated from three independent experiments.

Kinetics-driven control of activation specificity

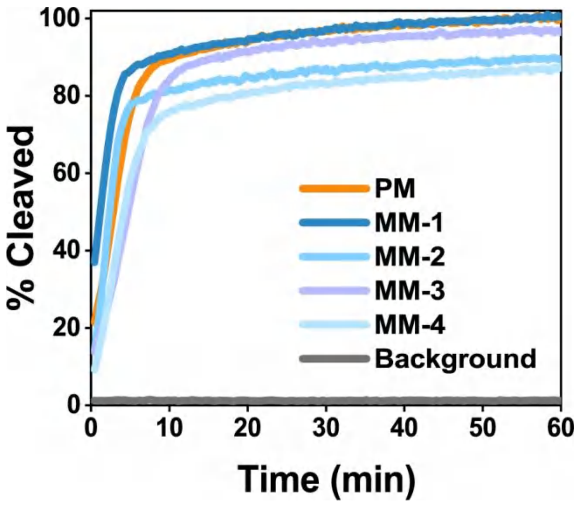

Figure S24. Real-time fluorescence kinetics of the activation efficiency of Cas12a without ERA by matched or mismatched ssDNA-activators.

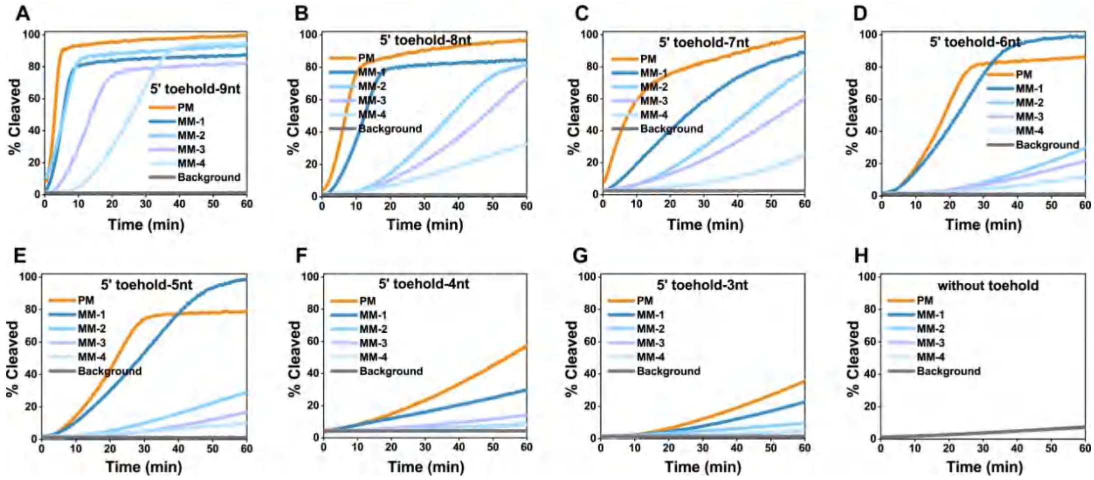

Figure S25. Real-time fluorescence kinetics of Cas12a activation by matched or mismatched ssDNA-activators, with ERAs of 5' toehold-9 nt (A), -8 nt (B), -7 nt (C), -6 nt (D), -5 nt (E), -4 nt (F), -3 nt (G)， and without toehold (H).

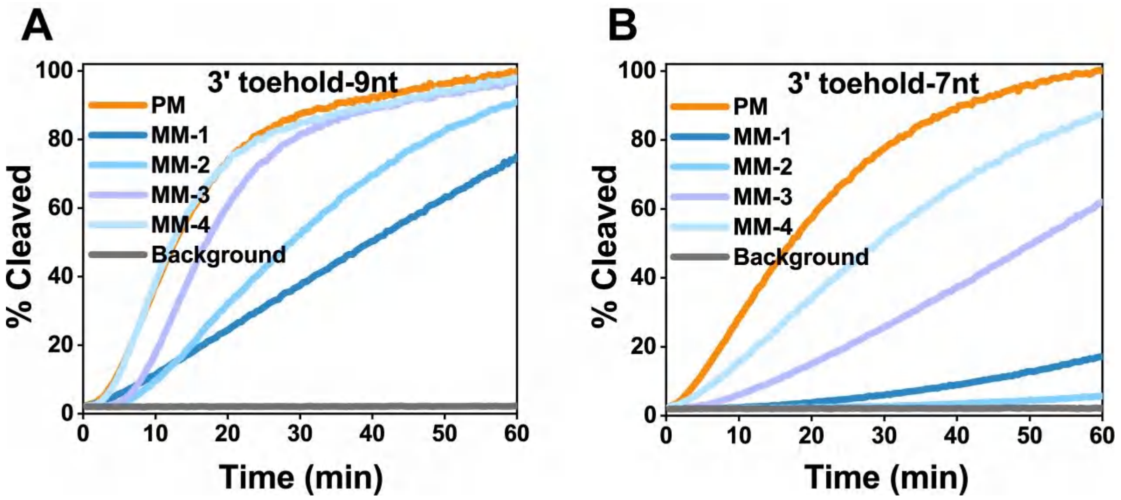

Figure S26. Real-time fluorescence kinetics of Cas12a activation by matched or mismatched ssDNA-activators, with ERAs of 3' toehold-9 nt (A) or -7 nt (B).

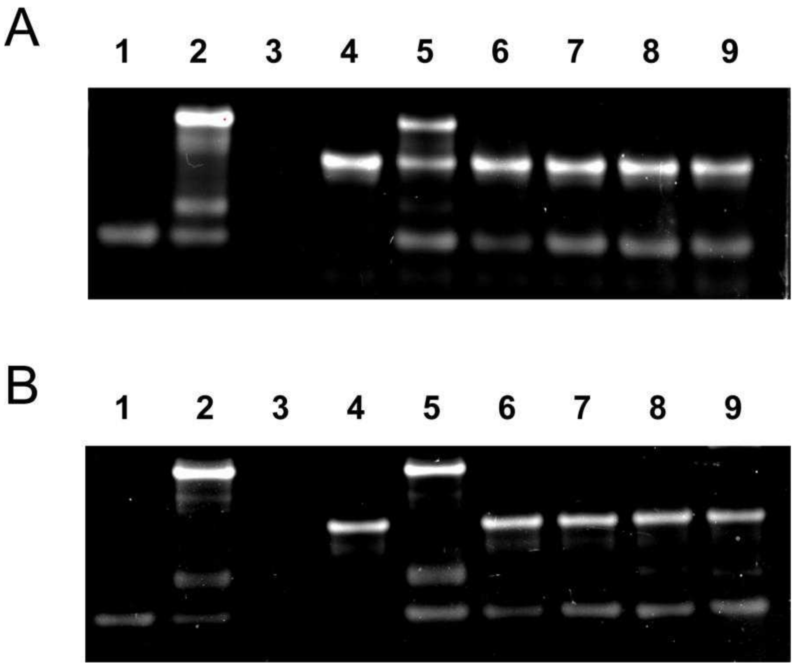

Figure S27. PAGE characterization of TMSD between ssDNA-activator and ERA/crRNA. (A) ERA is selected as 5' toehold-7 nt: (1) crRNA, (2) crRNA + PM, (3) ERA (5' toehold-7 nt), (4) crRNA + ERA, (5) crRNA/ERA+PM, (6) crRNA/ERA+MM-1, (7) crRNA/ERA+MM-2, (8) crRNA/ERA+MM-3, (9) crRNA/ERA+MM-4. (B) ERA is selected as 3' toehold-7 nt: all components are the same as (A) except for the ERA substitution. Since the difference in the bases number between ssDNA-activator and ERA is too small, in order to make a large difference in the migration position of the reaction product ssDNA-activator/crRNA to the substrate ERA/crRNA, we added a 5 nt-poly A on each side of ssDNA-activator, which does not interfere with the reaction.

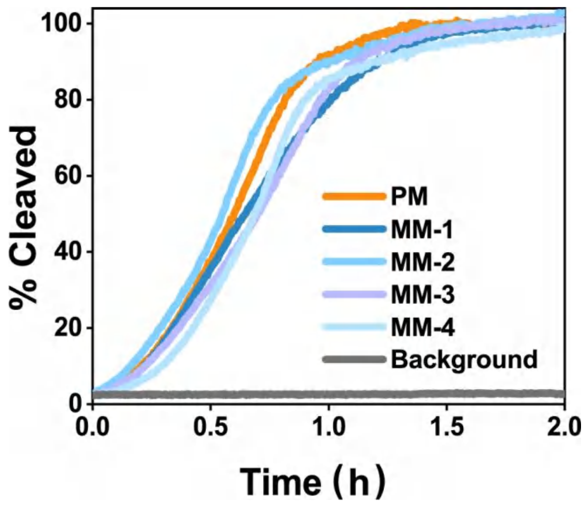

Figure S28. Real-time fluorescence kinetics of the activation efficiency of Cas12a without ERA by matched or mismatched dsDNA-activators.

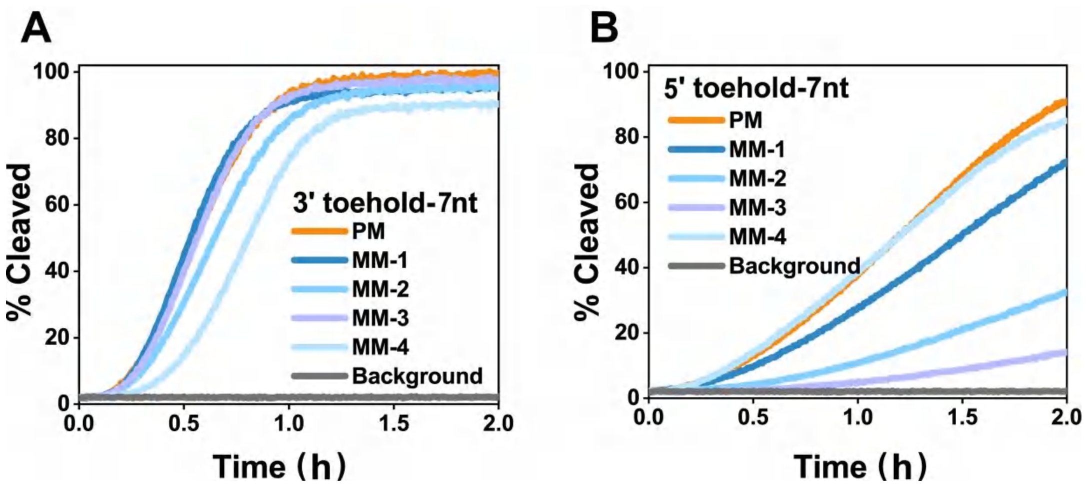

Figure S29. Real-time fluorescence kinetics of Cas12a activation by matched or mismatched dsDNA-activators, with 7 nt -ERAs of 3' toehold (A) or 5' toehold (B).

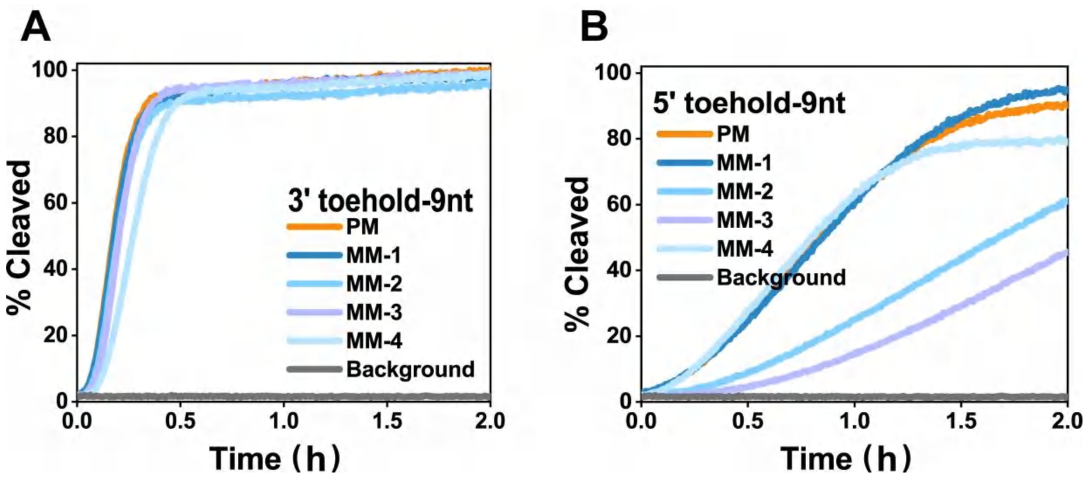

Figure S30. Real-time fluorescence kinetics of Cas12a activation by matched or mismatched dsDNA-activators, with 9 nt-ERAs of 3' toehold (A) or 5' toehold (B).

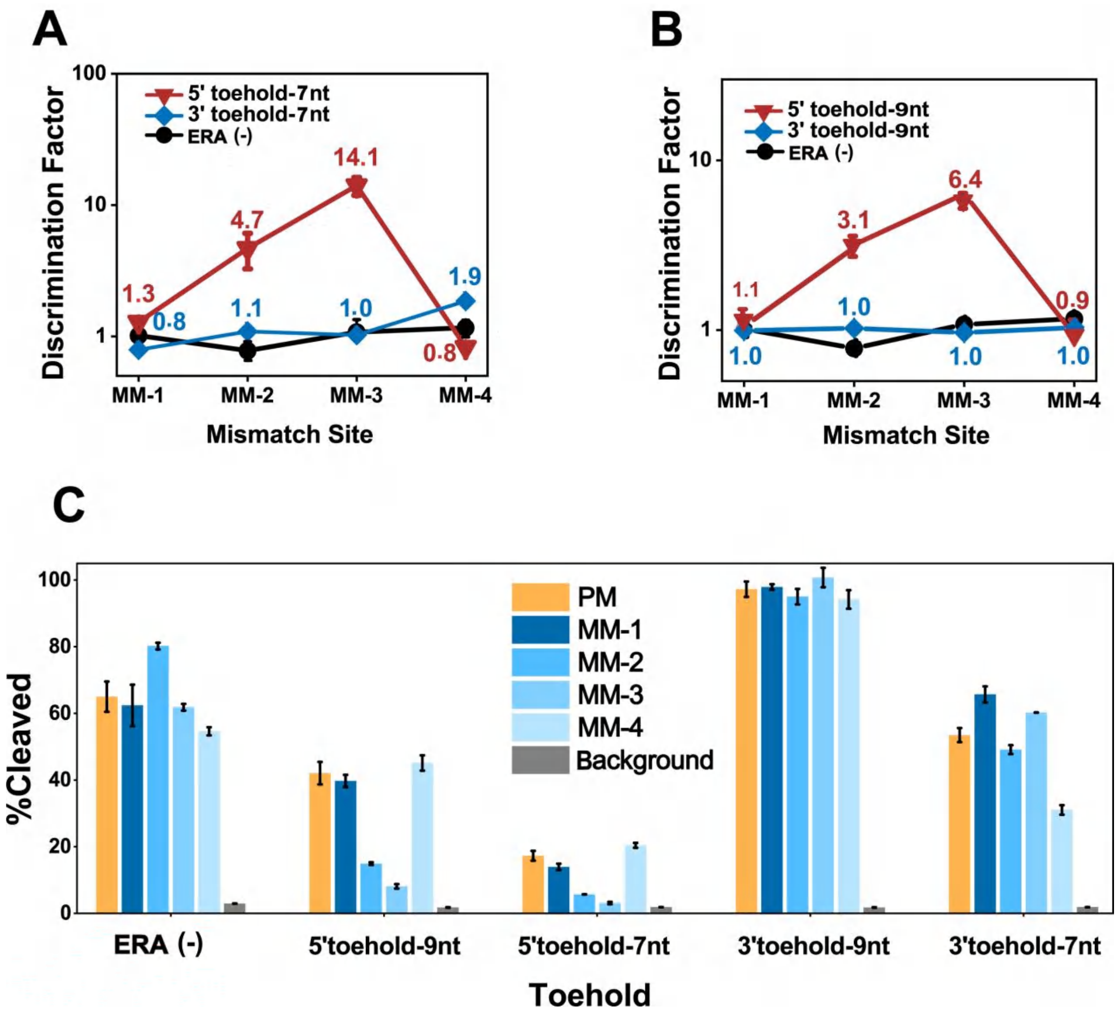

Figure S31. (A, B) Relationship between DF and mismatch site, toehold length and direction. (C) Efficiency of ERA-Cas12a activation at 40 min by different mismatched dsDNA-activators when toehold direction is 5' or 3' and length is 7 nt or 9 nt. ERA (-) means no ERA is added. Error bars represented the standard deviation calculated from three independent experiments.

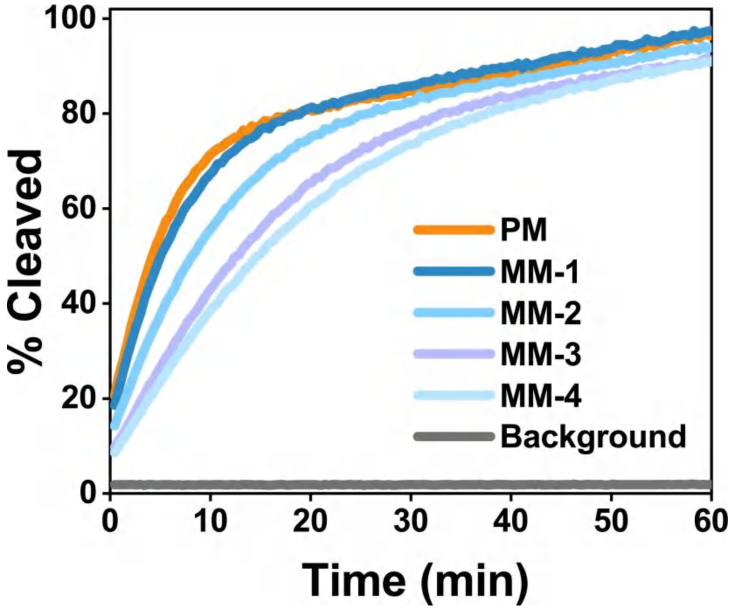

Figure S32. Real-time fluorescence kinetics of the activation efficiency of Cas12a without ERA-β by matched or mismatched ssDNA-activators.

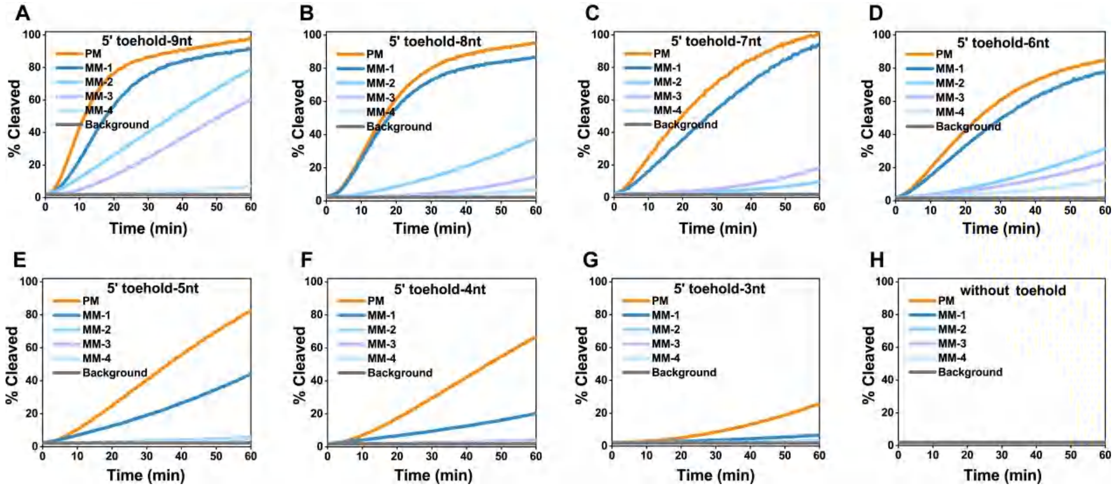

Figure S33. Real-time fluorescence kinetics of Cas12a activation by matched or mismatched ssDNA-activators, with ERAs-β of 5' toehold-9 nt (A), -8 nt (B), -7 nt (C), -6 nt (D), -5 nt (E), -4 nt (F), -3 nt (G), and without toehold (H).

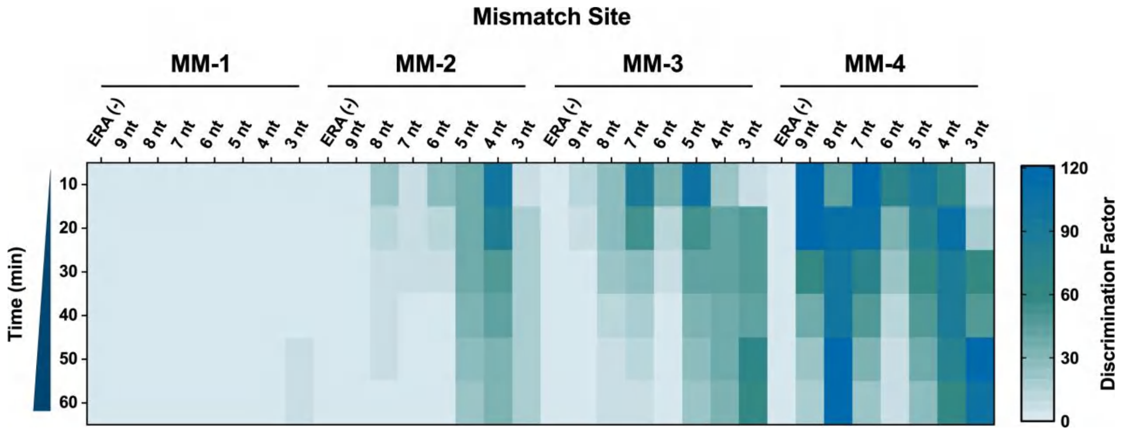

Figure S34. DF-time heat map of ERA-β-Cas12a on ssDNA activators.

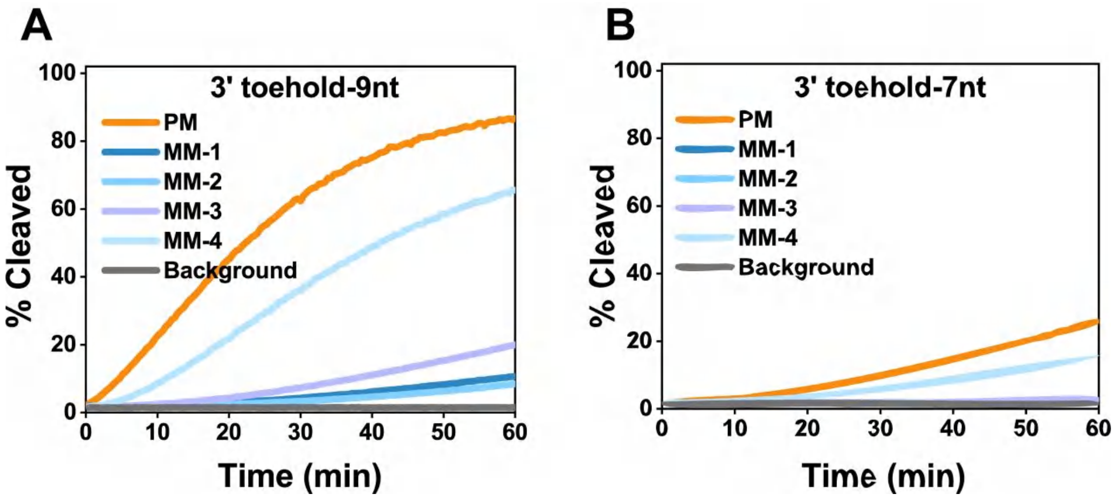

Figure S35. Real-time fluorescence kinetics of Cas12a activation by matched or mismatched ssDNA-activators, with ERAs-β of 3' toehold-9 nt (A) or -7 nt (B).

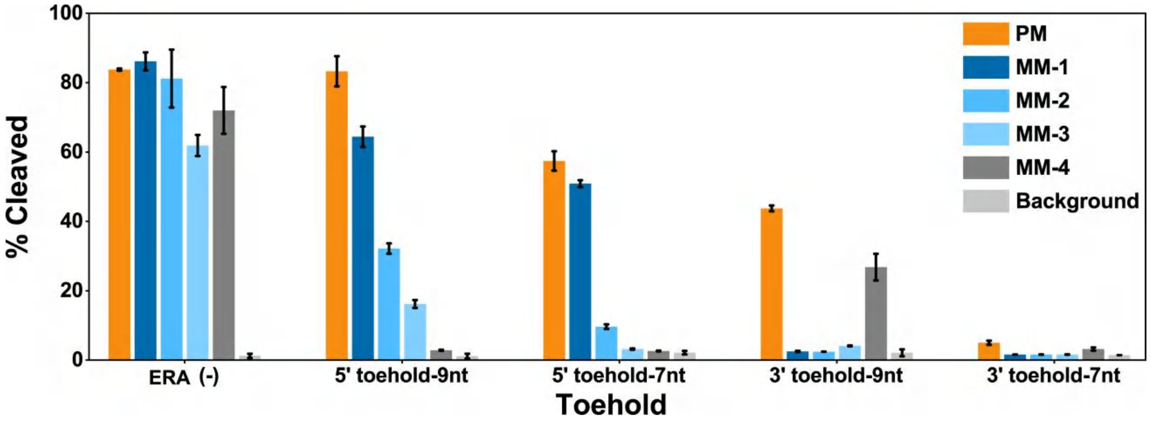

Figure S36. Efficiency of ERA-β-Cas12a activation at 40 min by different mismatched ds-activators when toehold-β direction is 5' or 3' and length is 7 nt or 9 nt. Error bars represented the standard deviation calculated from three independent experiments.

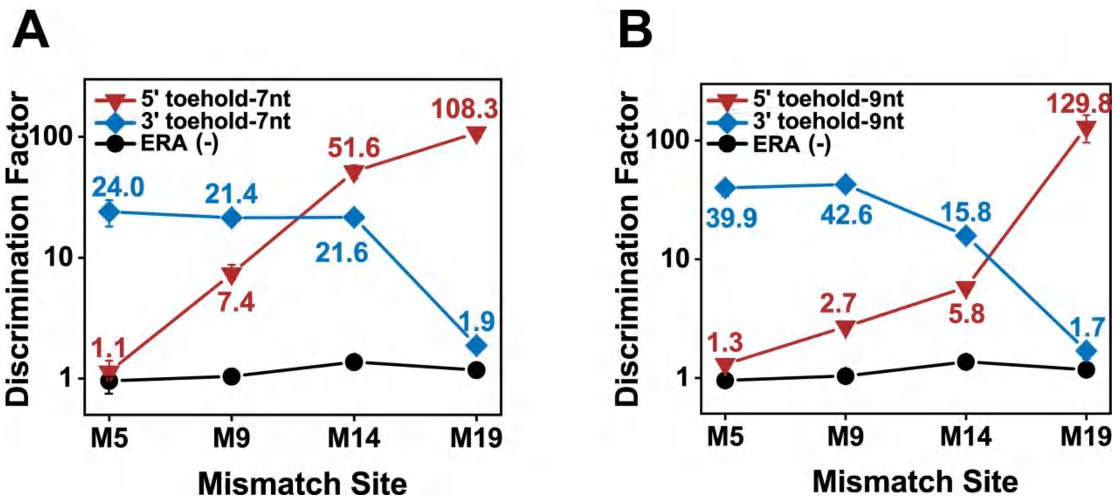

Figure S37. Relationship between DF of the mismatch site of sequence-β, toehold length and direction at 20 min. Error bars represented the standard deviation calculated from three independent experiments.

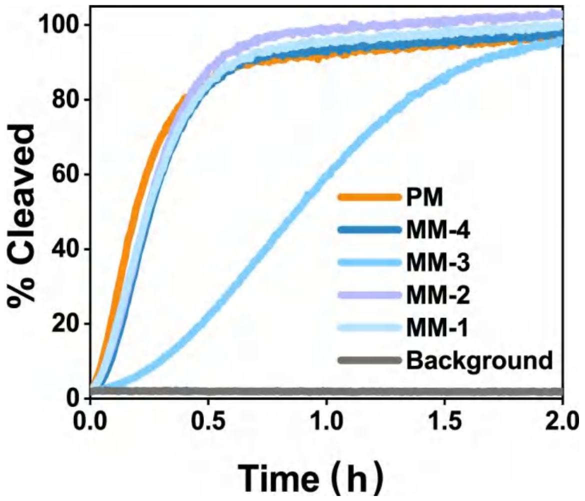

Figure S38. Real-time fluorescence kinetics of the activation efficiency of Cas12a without ERA-β by matched or mismatched dsDNA-activators.

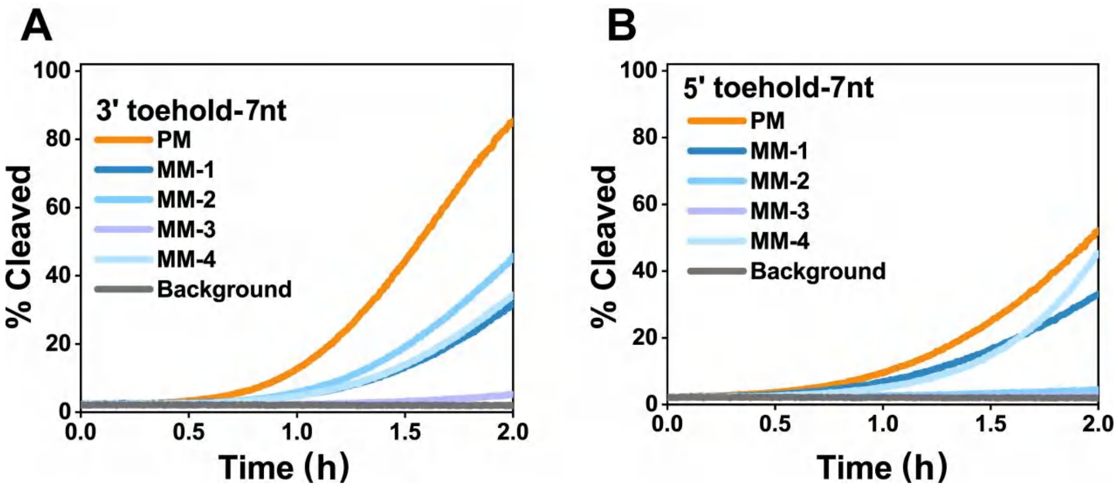

Figure S39. Real-time fluorescence kinetics of Cas12a activation by matched or mismatched dsDNA-activators, with ERAs-β of 3' toehold-7 nt (A) or 5' toehold-7 nt (B).

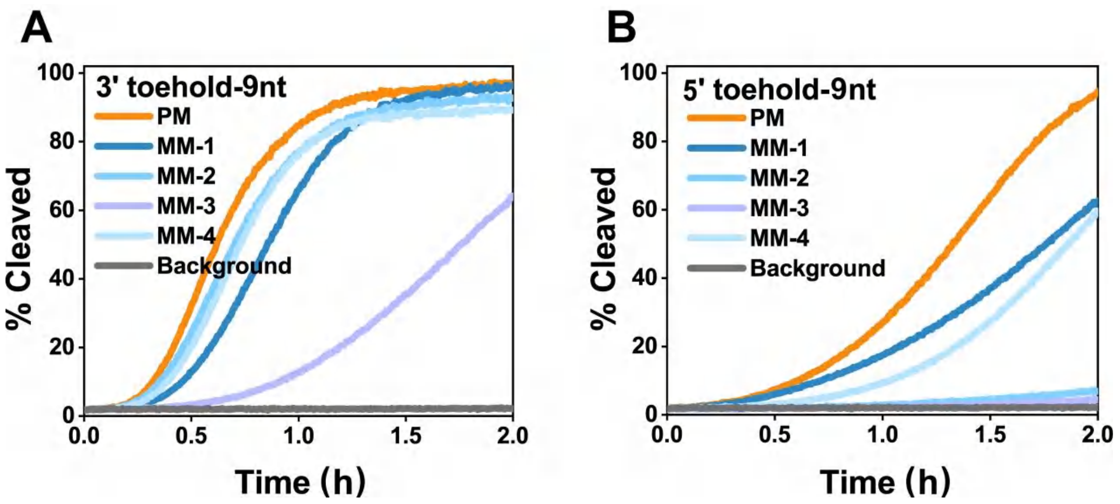

Figure S40. Real-time fluorescence kinetics of Cas12a activation by matched or mismatched dsDNA-activators, with ERAs-β of 3' toehold-9 nt (A) or 5' toehold-9 nt (B).

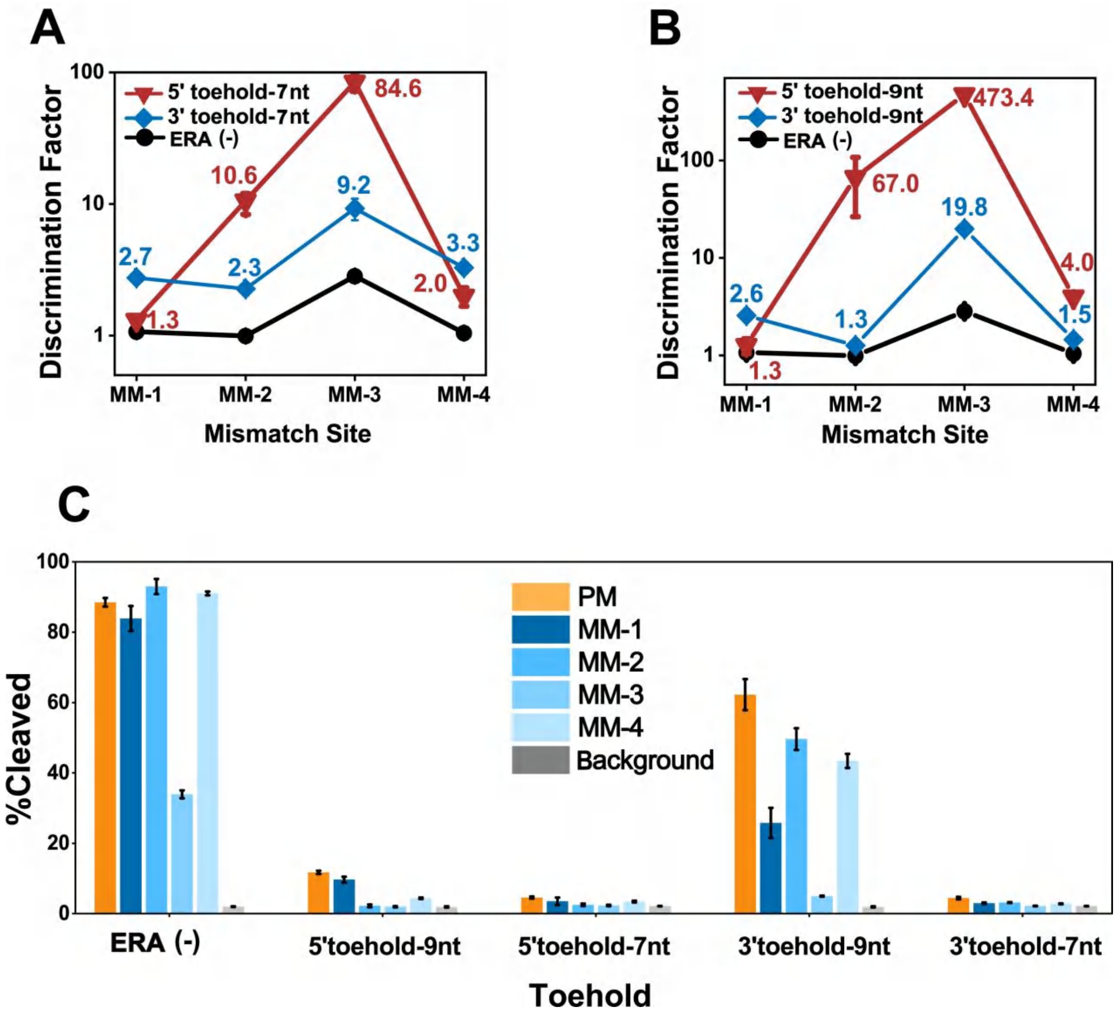

Figure S41. (A, B) Relationship between DF and mismatch site of sequence-β, toehold length and direction at 40 min. (C) Efficiency of ERA-β-Cas12a activation by different mismatched dsDNA-activators at 40 min when toehold direction is 5' or 3' and length is 7 nt or 9 nt. Error bars represented the standard deviation calculated from three independent experiments.

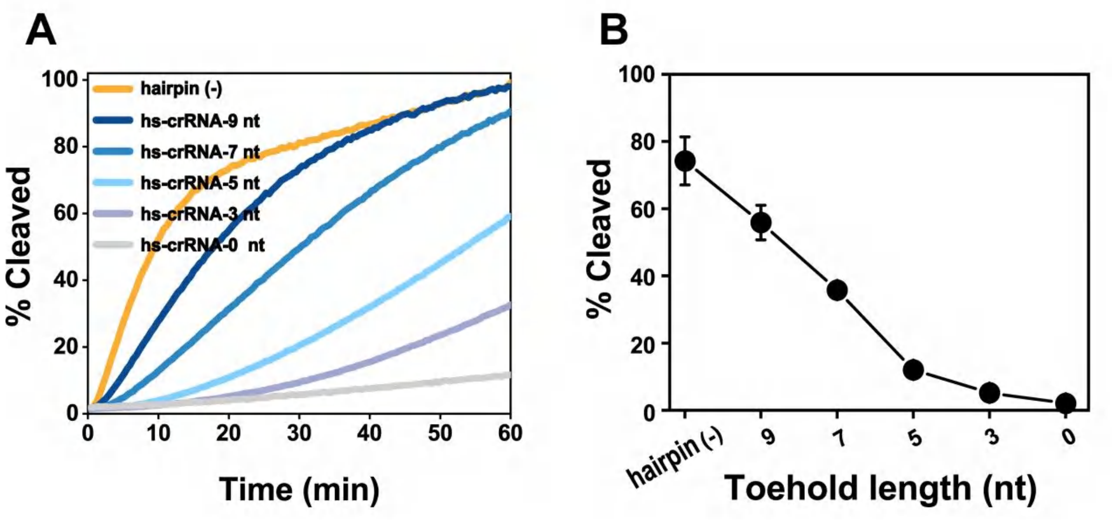

Figure S42. Real-time fluorescence curve (A) and fluorescence cleavage rate at 20 min (B) of hs-crRNA- Cas12a with different toehold length. Hairpin (-) means that there is no hairpin structure added to the crRNA. Error bars represented the standard deviation calculated from three independent experiments.

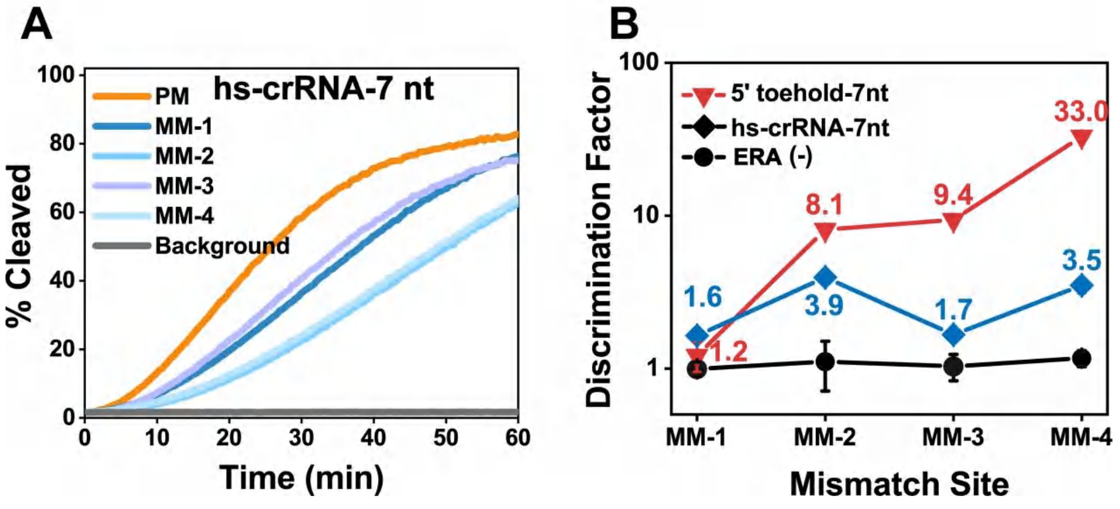

Figure S43. (A) Real-time fluorescence curve of hs-crRNA-toehold/7 nt controlled Cas12a. (B) Comparison of the discrimination ability of hairpin-controlled with ERA-controlled Cas12a for the same toehold length (7nt) at 20 min. ERA (-) means no ERA is added. Error bars represented the standard deviation calculated from three independent experiments.

Scalability based on DNA nanotechnology

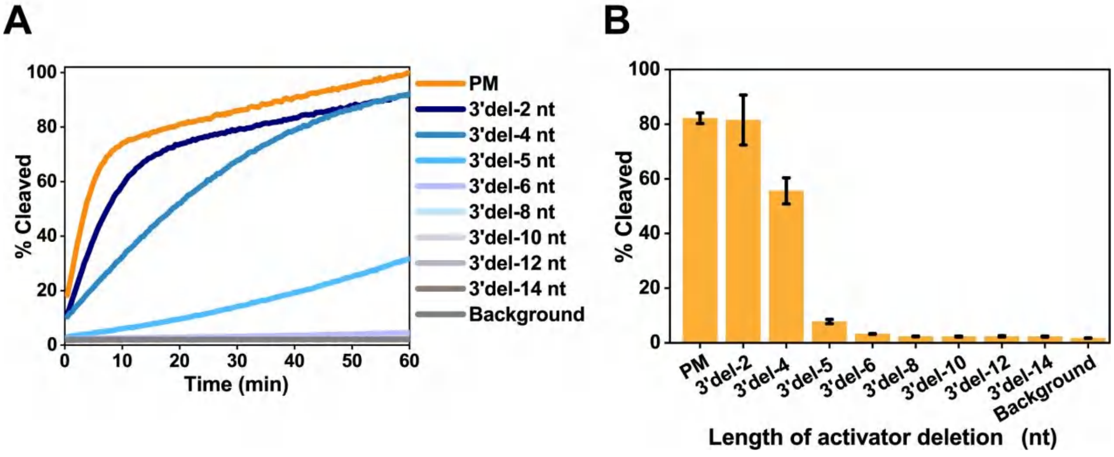

Figure S44. Effect of 3' end deletion of the ssDNA-activator on cleavage rate when the spacer of crRNA is 22 nt, fluorescence kinetic curve (A) and cleavage rate at 20 min (B). Error bars represented the standard deviation calculated from three independent experiments.

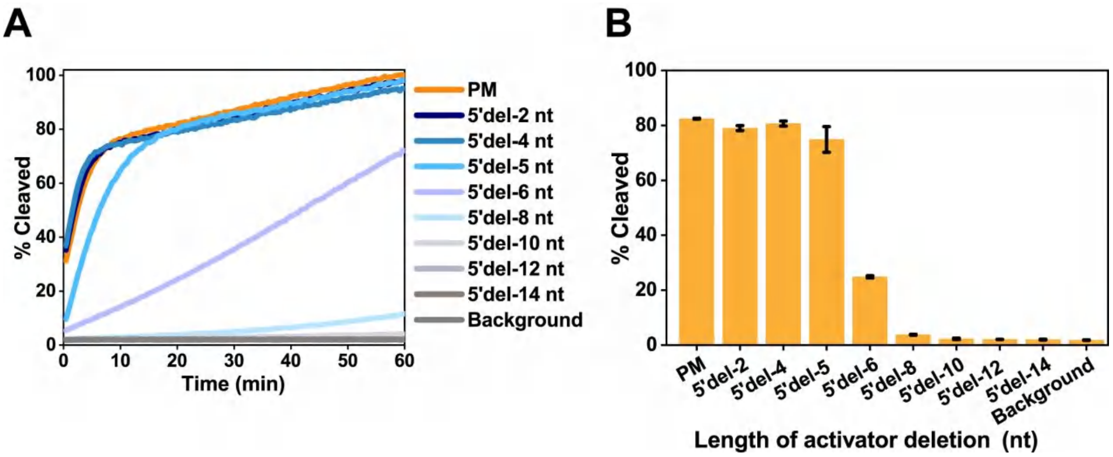

Figure S45. Effect of 5' end deletion of the ssDNA-activator on cleavage rate when the spacer of crRNA was 22 nt, fluorescence kinetic curve (A) and cleavage rate at 20 min (B). Error bars represented the standard deviation calculated from three independent experiments.

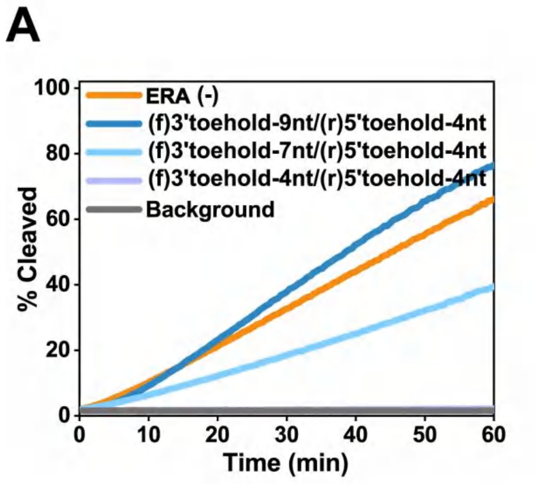

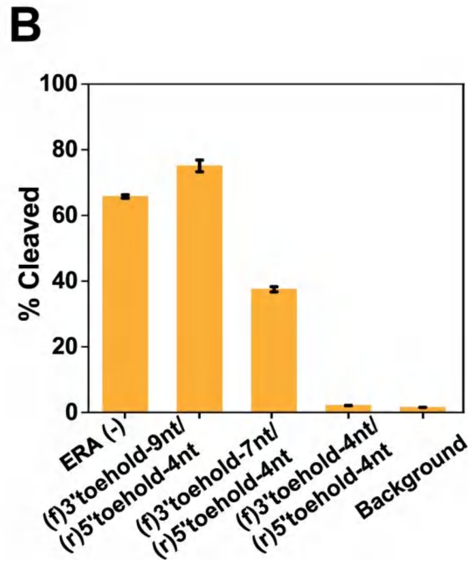

Figure S46. Fluorescence kinetic graph (A) and cleavage rate at 20 min (B) When the crRNA spacer was 22 nt, the 5' toehold(f) was fixed at 4 nt to find the optimal 3' toehold(r) length. Error bars represented the standard deviation calculated from three independent experiments.

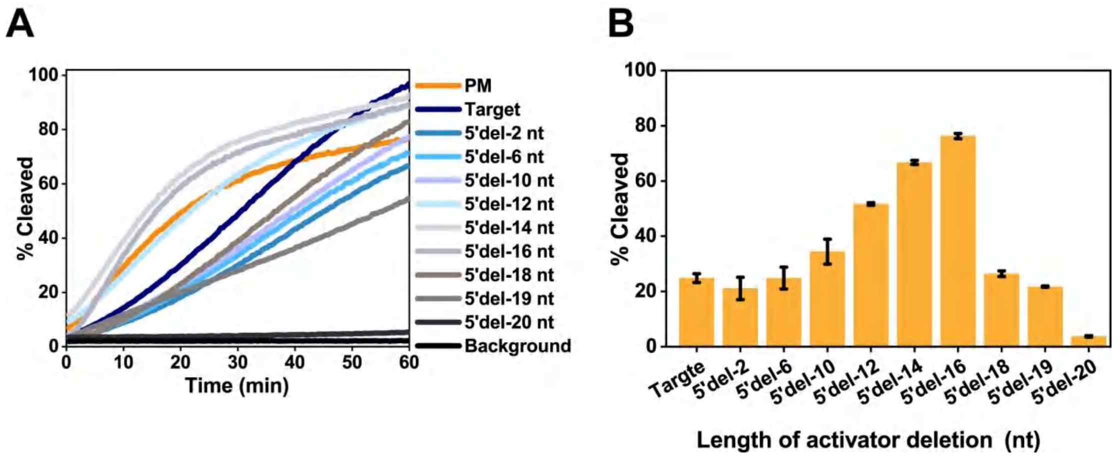

Figure S47. Effect of activator-δ 3' end deletion on cleavage rate by extending spacer to 35nt (crRNA-δ). Fluorescence kinetic graph (A) and cleavage rate at 20 min (B). Error bars represented the standard deviation calculated from three independent experiments.

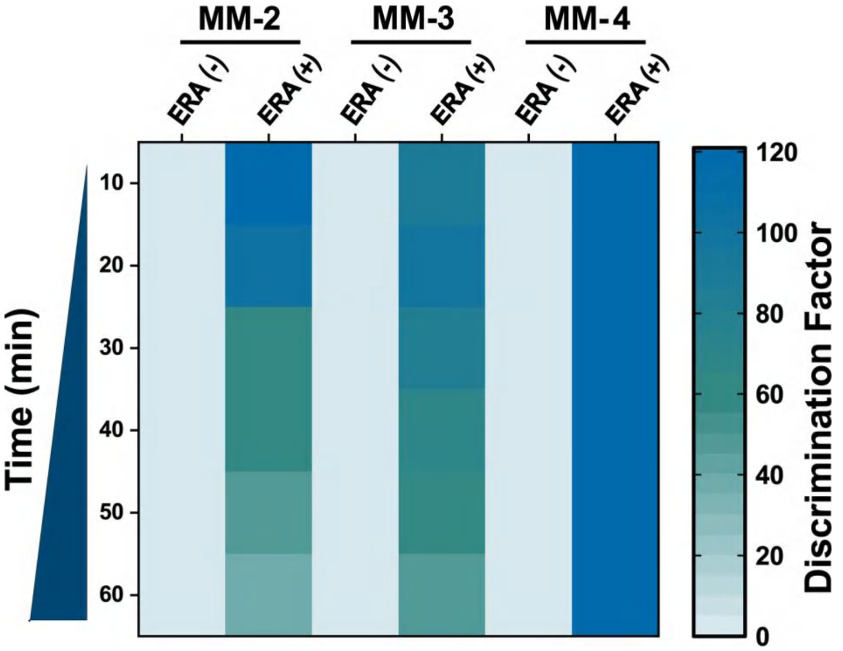

Figure S48. DF-time heat map of (f) 5'-5 nt/(r) 5'-5 nt TE-based ERA-Cas12a dentifying different mismatched ssDNA-activators at fixed spacer=22 nt. ERA (-) means no ERA is added, while ERA (+) means ERA is added.

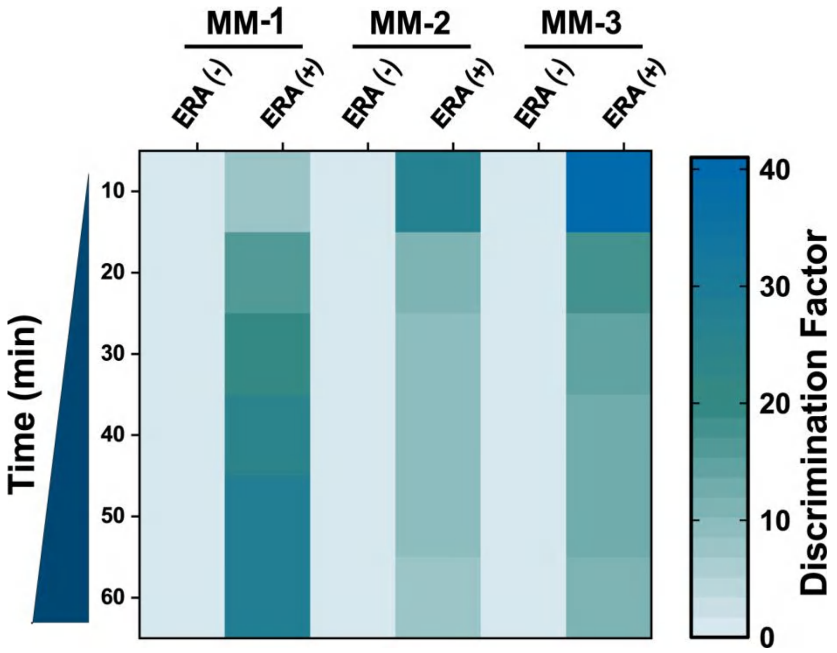

Figure S49. DF-time heat map of (f) 3'-4 nt/(r) 5'-9 nt TE-based ERA-Cas12a dentifying different mismatched ssDNA-activators at fixed spacer=22 nt. ERA (-) means no ERA is added, while ERA (+) means ERA is added.

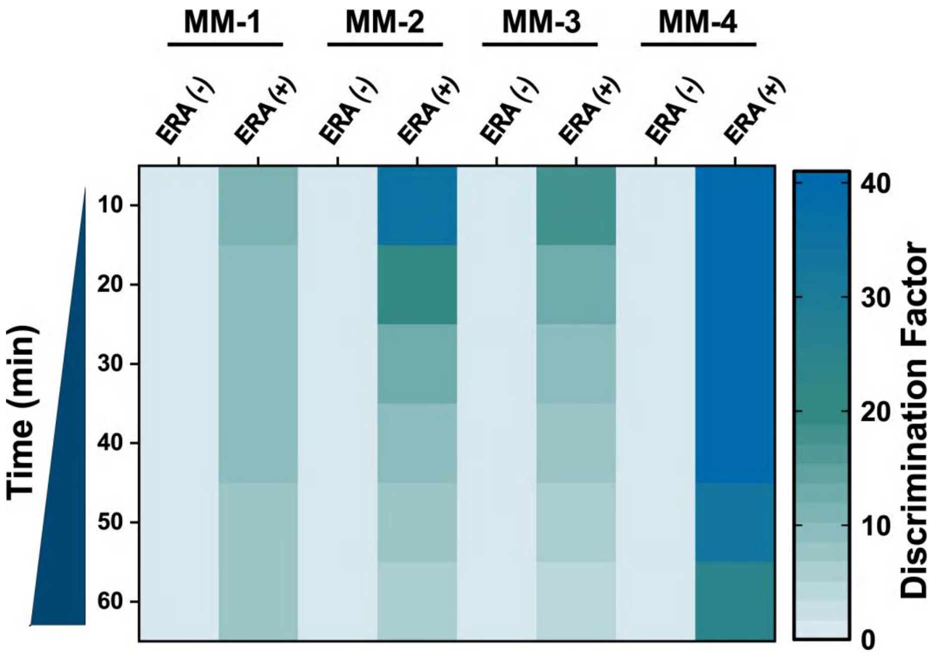

Figure S50. DF-time heat map of (f) 5'-7 nt/(r) 3'-7 nt TE-based ERA-Cas12a dentifying different mismatched ssDNA-activators at extended spacer=29 nt. ERA (-) means no ERA is added, while ERA (+) means ERA is added.

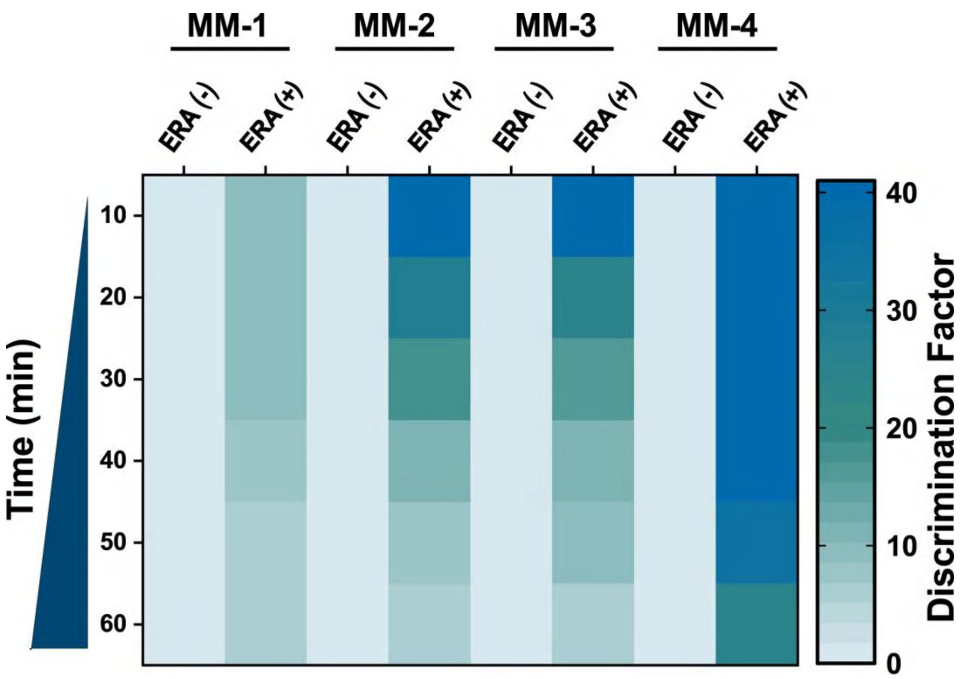

Figure S51. DF-time heat map of (f) 5'-7 nt/(r) 3'-13 nt TE-based ERA-Cas12a dentifying different mismatched ssDNA-activators at extended spacer=35 nt. ERA (-) means no ERA is added, while ERA (+) means ERA is added.

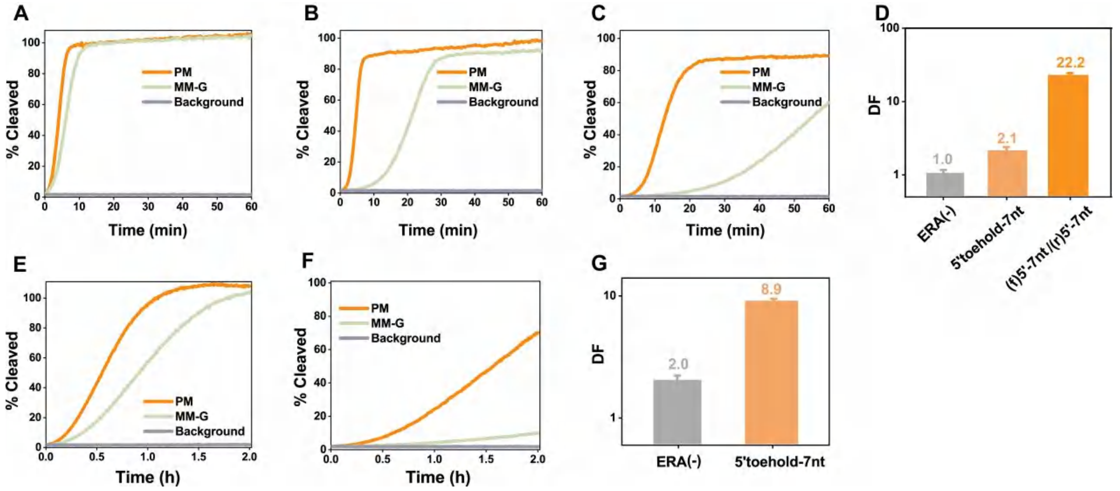

Figure S52. G-U mismatches (wobble) identification. Real-time fluorescence kinetics of Cas12a without ERA-control (A), controlled by 5' toehold-7 nt (B) or (f)5'-7 nt/ (r)3'-7 nt (C) when activated by ss-PM and MM-G . (D) Discrimination factors at 20min in (A), (B) and (C). Real-time fluorescence kinetics of Cas12a without ERA-control (E) or controlled by 5' toehold-7 nt (F) when activated ds-PM and MM-G. (G) Discrimination factors at 40min in (E) and (F). Error bars represented the standard deviation calculated from three independent experiments.

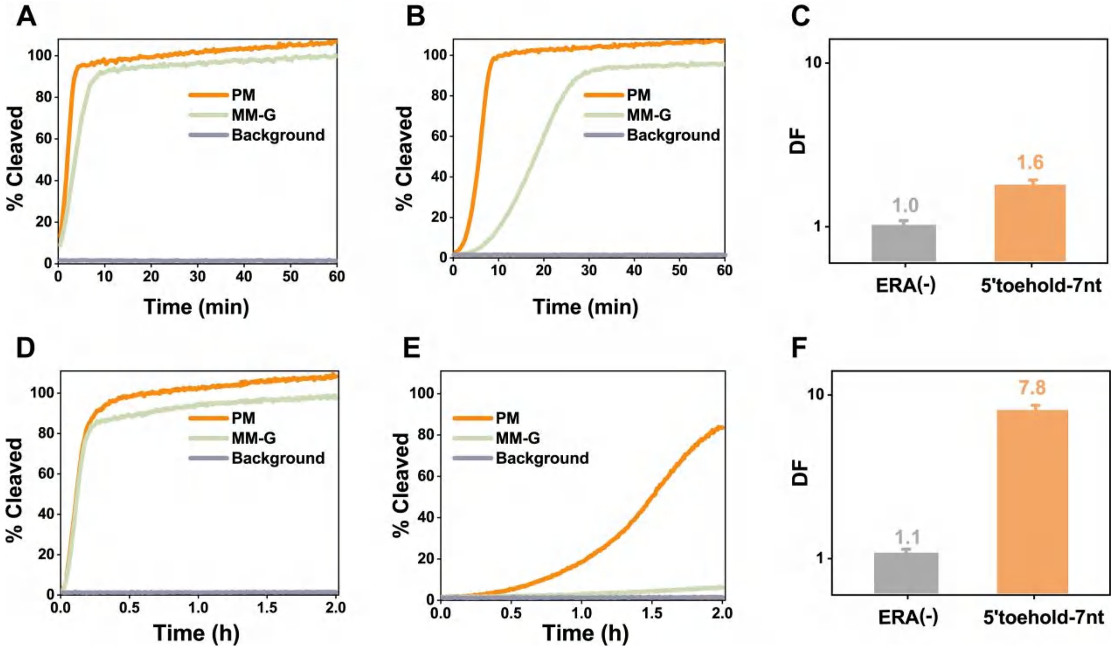

Figure S53. G-U mismatches (wobble) identification. Real-time fluorescence kinetics of Cas12a without ERA-β-control (A), or controlled by 5' toehold-7 nt (B) when activated by ss-PM-β and MM-G-β. (C) Discrimination factors at 20min in (A) and (B). Real-time fluorescence kinetics of Cas12a without ERA-β-control (D) or controlled by 5' toehold-7 nt (E) when activated by ds-PM-β and ds-MM-G-β. (F) Discrimination factors at 40min in (E) and (F). Error bars represented the standard deviation calculated from three independent experiments.

Spatio-temporal continuity control in isothermal one-pot assay

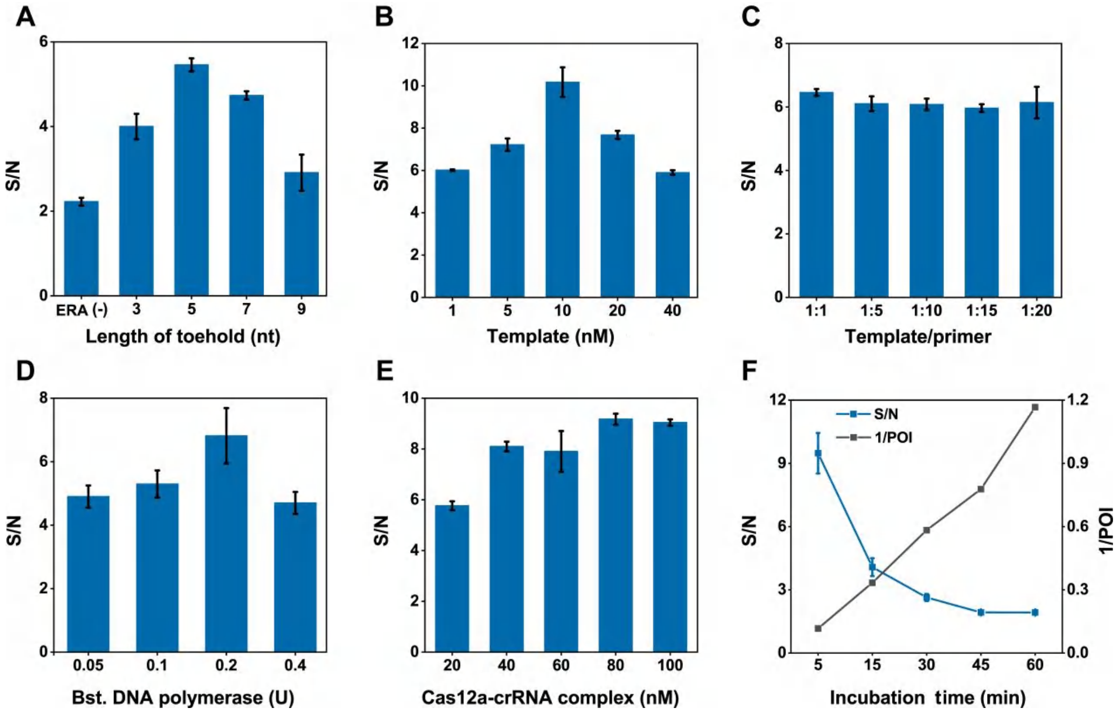

Figure S54. Optimized experimental conditions for PER reaction. (A) Optimization of toehold length, 5nt is optimal. (B) Optimization of template concentration, 10 nM is optimal. (C) Optimization of template/primer ratio, 1:20 is optimal.(D) Optimization of Bst.DNA polymerase concentration, 0.2 U is optimal. (E) Optimization of Cas12a-crRNA complex concentration, 80 nM is chosen. (F) PER incubation time optimization for two-step assay, 30 min is chosen. ERA (-) means no ERA is added. Error bars represented the standard deviation calculated from three independent experiments.

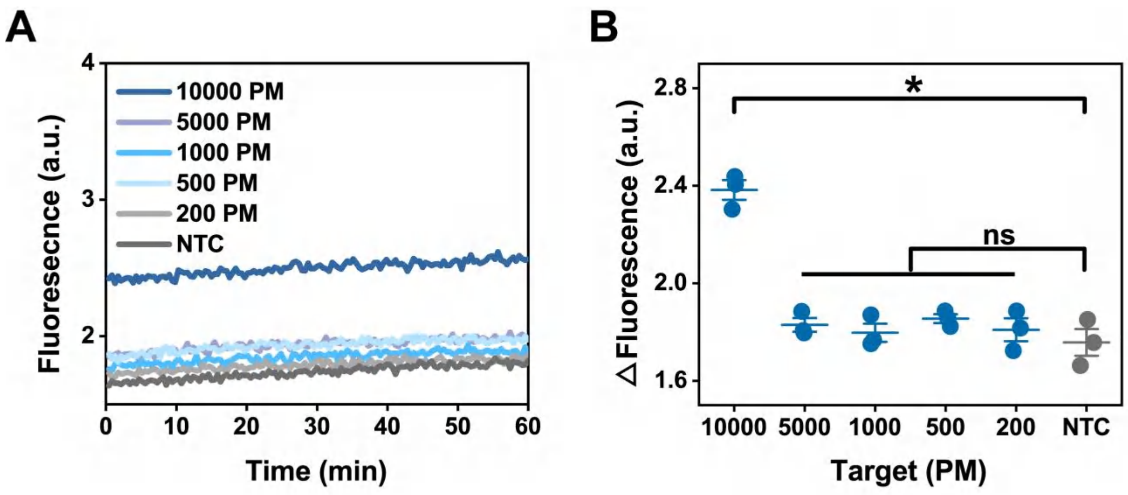

Figure S55. Sensitivity of the PER alone. NTC represents no target control. *, $P < 0.05$; **, $P < 0.01$; ***, $P < 0.001$; ****, $P < 0.0001$; ns, not significant.

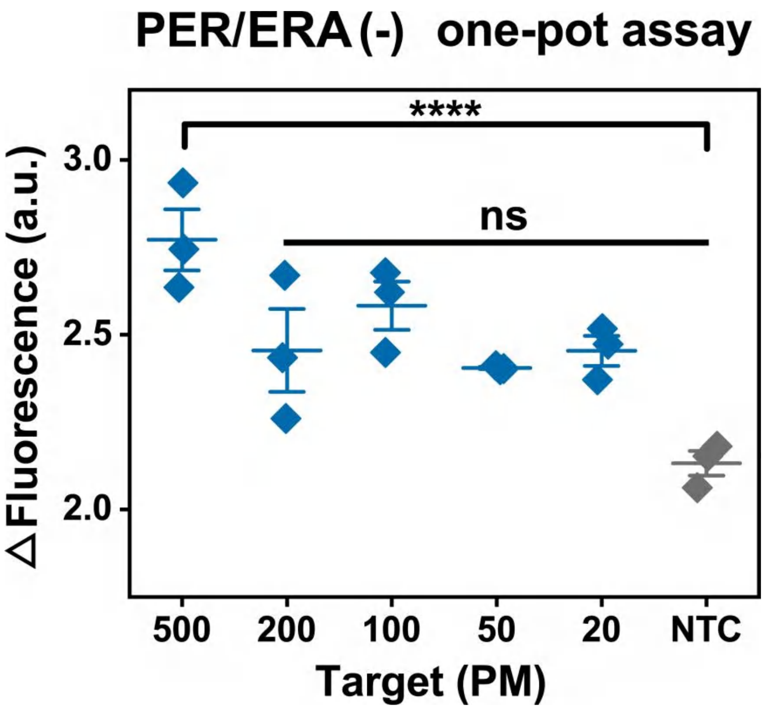

Figure S56. Sensitivity of one-pot assay without ERA support. NTC represents no target control. *, $P < 0.05$; **, $P < 0.01$; ***, $P < 0.001$; ****, $P < 0.0001$; ns, not significant.

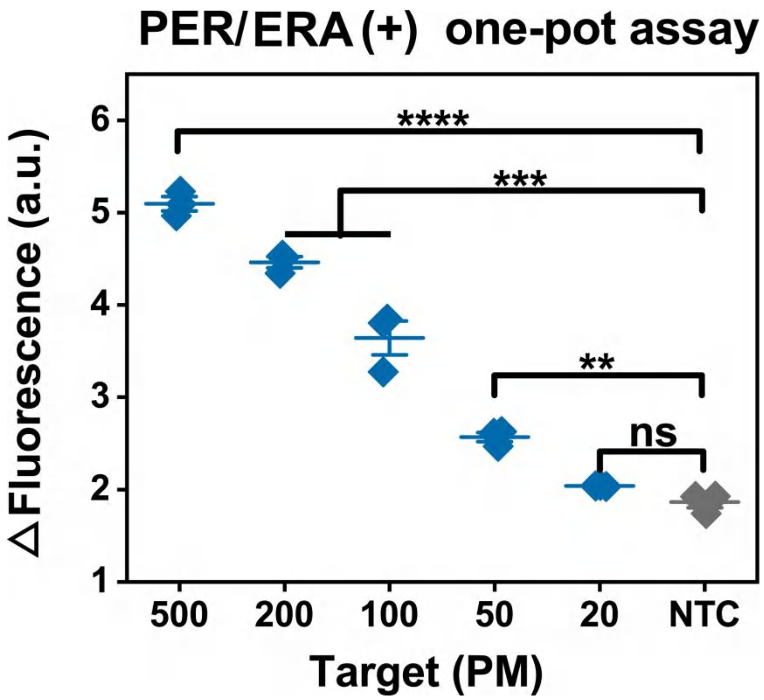

Figure S57. Sensitivity of one-pot assay with ERA support. NTC represents no target control. *, $P < 0.05$; **, $P < 0.01$; ***, $P < 0.001$; ****, $P < 0.0001$; ns, not significant.

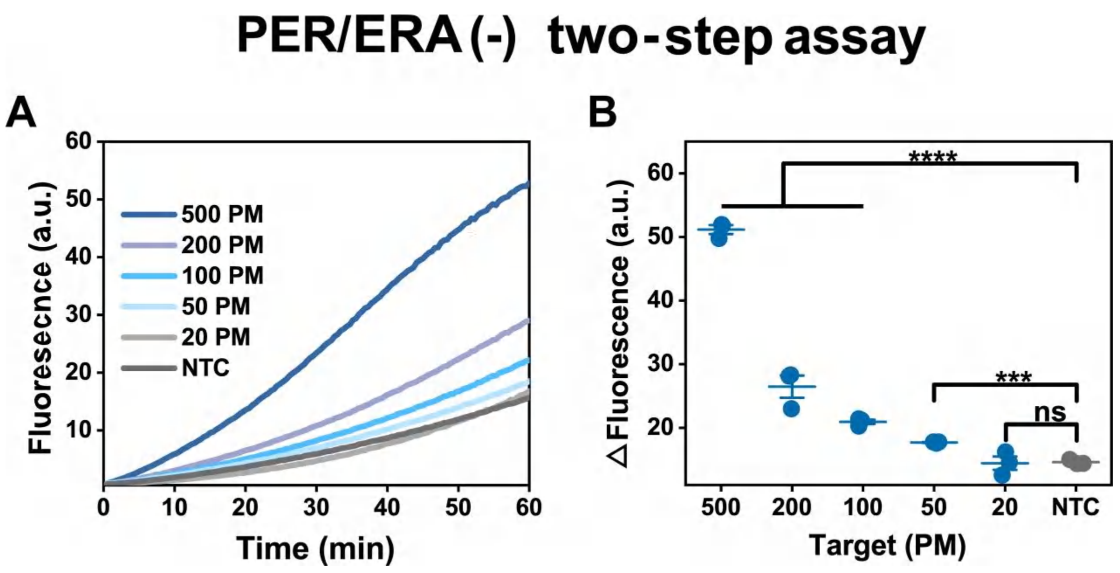

Figure S58. Sensitivity of traditonal two-step assay. NTC represents no target control.*, $P < 0.05$; **, $P < 0.01$; ***, $P < 0.001$; ****, $P < 0.0001$; ns, not significant.

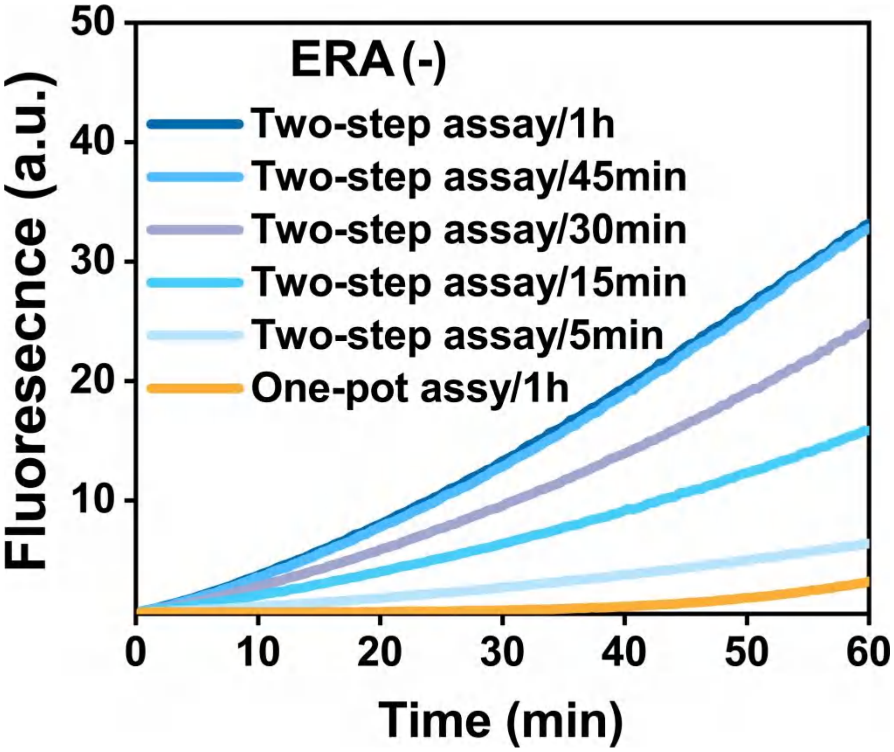

Figure S59. Leakage-time comparison of the one-pot and two-step assay without ERA support.

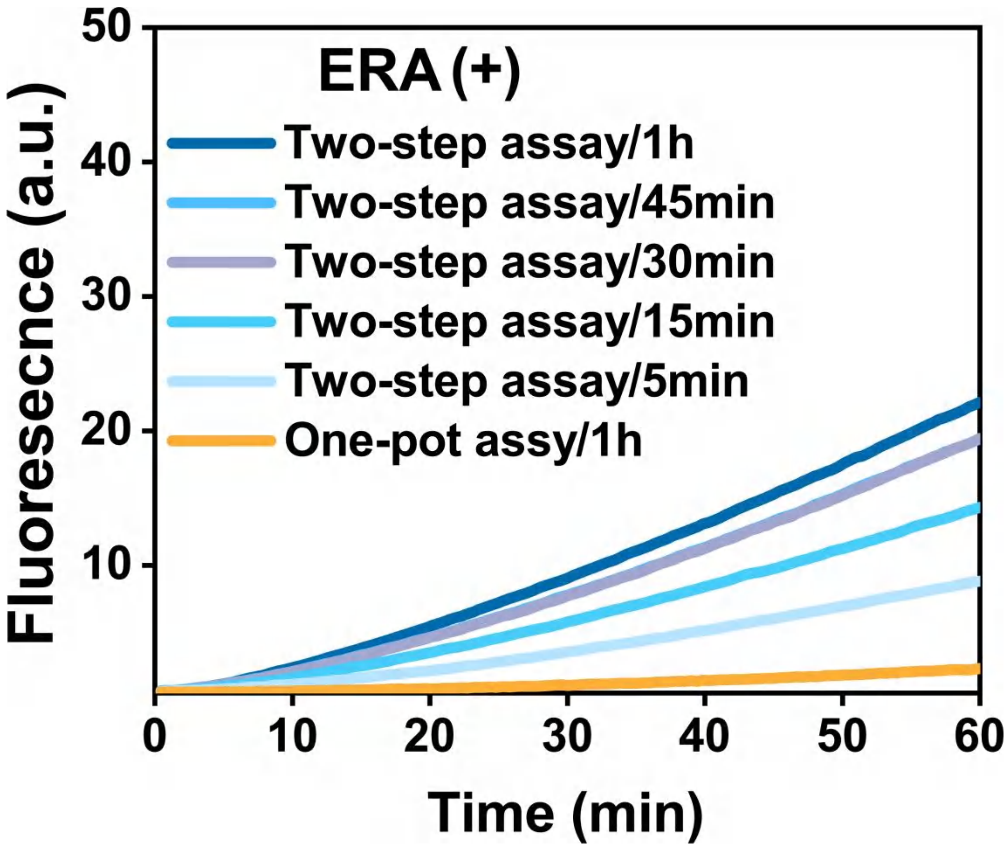

Figure S60. Leakage-time comparison of the one-pot and two-step assay with ERA support.
